# Secondary Metabolites and Their Biological Evaluation from the Aerial Parts of *Staehelina uniflosculosa* Sibth. & Sm. (Asteraceae)

**DOI:** 10.3390/ijms251910586

**Published:** 2024-10-01

**Authors:** Maria Lazanaki, George Tsikalas, Olga S. Tsiftsoglou, Haralambos Katerinopoulos, Dimitra Hadjipavlou-Litina, Diamanto Lazari

**Affiliations:** 1 Laboratory of Pharmacognosy, Faculty of Health Sciences, School of Pharmacy, Aristotle University of Thessaloniki, 54124 Thessaloniki, Greece; mlazanak@yahoo.gr (M.L.); olga@tsiftsoglou.gr (O.S.T.); 2 Department of Chemistry, Division of Organic Chemistry, University of Crete, 71003 Heraklion, Greece; gtsikalas@chemistry.uoc.gr (G.T.); kater@chemistry.uoc.gr (H.K.); 3 Department of Pharmaceutical Chemistry, Faculty of Health Sciences, School of Pharmacy, Aristotle University of Thessaloniki, 54124 Thessaloniki, Greece; hadjipav@pharm.auth.gr

**Keywords:** sesquiterpene lactones, flavonoids, nuclear receptor ligand

## Abstract

Phytochemical investigation of *Staehelina uniflosculosa* Sibth. & Sm. resulted in the isolation of twenty-two natural products: eleven sesquiterpene lactones, artemorin (**1**), tamirin (**2**), tanachin (**3**), reynosin (**4**), baynol C (**5**), desacetyl-β-cyclopyrethrosin (**6**), 1β-hydroxy-4α-methoxy-5α,7α,6β-eudesm-11(13)-en-6,12-olide (**7**), 1β,4α,6α-trihydroxyeudesm-11-en-8α,12-olide (**8**), 1β-hydroxy-arbusculin A (**9**), methyl-1β,4α,6α-trihydroxy-5α,7αH-eudesm-11(13)-en-12-oate (**10**) and methyl-1β,6α,8α-trihydroxy-5α,7αH-eudesma-4(15),11(13)-dien-12-oate (**11**); one lignan, pinoresinol (**12**); one norisoprenoid, loliolide (**13**); six flavonoids (four genins and two glycosides), hispidulin (**14**), nepetin (**15**), jaceosidin (**16**), eriodictyol (**17**), eriodictyol-3′-O-β-D-glucoside (**18**) and eriodictyol-7-O-β-D-glucuronide (**19**); and three phenolic derivatives (one phenolic acid and two phenolic glucosides), protocatechuic acid (**20**), arbutin (**21**) and nebrodenside A (**22**). From the isolated compounds, only nepetin (**15**) has been reported previously from the *Staehelina* genus and, to the best of our knowledge, it is the first time that compound (**18**) has been identified in Asteraceae. A number of these substances were tested for (a) inhibition of lipoxygenase and acetylocholinesterase, (b) their antioxidant activity using the DPPH (1,1-Diphenyl-2-picrylhydrazyl) method or/and (c) inhibition of lipid peroxidation. The tested components exhibited low antioxidant activity with the exception of **5** and **22**, while the effectiveness of these compounds in the inhibition of acetylocholinesterase is limited. Furthermore, Molinspiration, an online computer tool, was used to determine the bioactivity ratings of the isolated secondary metabolites. The compounds’ bioactivity ratings for potential therapeutic targets were very promising.

## 1. Introduction

The genus *Staehelina* (Asteraceae, tribe Cardueae) is very limited and consists of seven or eight species, which grow almost exclusively in the Mediterranean area (Albania, Algeria, Baleares, Corse, France, Greece, Italy, Morocco, Portugal, Spain, Tunisia and Yugoslavia) [1,2]. This study is a part of our ongoing phytochemical studies on plants from the Asteraceae family. Components isolated from these plants are mainly monoterpenes, diterpenes, sesquiterpene lactones [3,4] and flavonoids [5]. Plants species belonging to the Asteraceae family are used in folk medicine and as herbal remedies [6,7].

**Figure 1:**
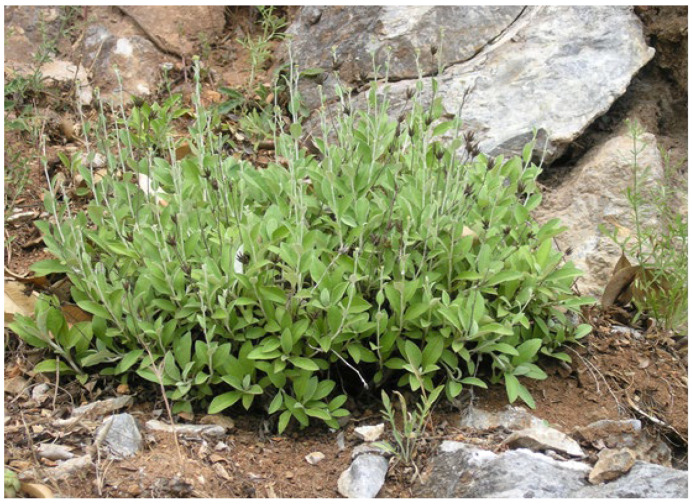
Wild-growing *Staehelina uniflosculosa* (photo by D. Lazari).

*Staehelina uniflosculosa* Sibth. & Sm. is a Balkan endemic species and is distributed in Albania, Greece and Yugoslavia (https://powo.science.kew.org/taxon/urn:lsid:ipni.org:names:250965-1 (accessed on 12 May 2024)). In Greece, it grows mainly in the mainland, in Euboea and in the North Peloponnese [8]. Aerial parts of *S. uniflosculosa* were collected from Prionia (Mountain Olympus, NC Greece) in June 2006. The plant was identified by Dr. N. Krigas, and a voucher specimen has been deposited at the Herbarium of Laboratory of Pharmacognosy of School of Pharmacy of AUTH under the code “Lazari 41” (Figure 1).

Only two species of *Staehelina* have been studied previously: *S. dubia* L. [9,10] and *S. fruticosa* L. [11]. Both are reported to contain mainly sesquiterpene lactones. To date, the chemical composition of *S. uniflosculosa* has not been studied.

Some members of the Asteraceae family have been used for their culinary and medicinal properties for over 3000 years. The majority of these plants have therapeutic benefits and a lengthy history in traditional medicine. Members of the Asteraceae family exhibit a variety of hepatoprotective, antioxidant, antibacterial and anti-inflammatory properties [12]. Terpenoids such as sesquiterpenoids, alkaloids, phenolics, flavonoids and polyacetylenes are just a few of the many bioactive substances that make the Asteraceae family so important from a biochemical and pharmacological standpoint [3]. Due to the lack of both ethnopharmacological and pharmacological data about the species of genus *Staehelina*, in the present study we examine the acetylocholinesterase, antioxidant and anti-inflammatory activities of the isolated secondary metabolites that are mentioned here.

Recent analysis of several traditional medicinal plants using more advanced techniques has produced a number of intriguing chemicals. These substances originating from plants can be utilized to create whole new medications or to modify ones that already exist [12]. A varied and fascinating source for the identification of novel lead structures is natural products. In the 1990s, numerous pharmaceutical corporations withdrew their investments in natural product-driven medication development, following an era of successful natural product research [13]. In this report, we elucidate the isolation process of 22 pure compounds derived from *S. uniflosculosa* and we examine their potent relation with target receptors (e.g., protease inhibitors, nuclear receptors and ion channel modulators) according to the Molinspiration cheminformatics program, assessing their physicochemical properties.

## 2. Results

### 2.1. Isolated Compounds

**Figure 2:**
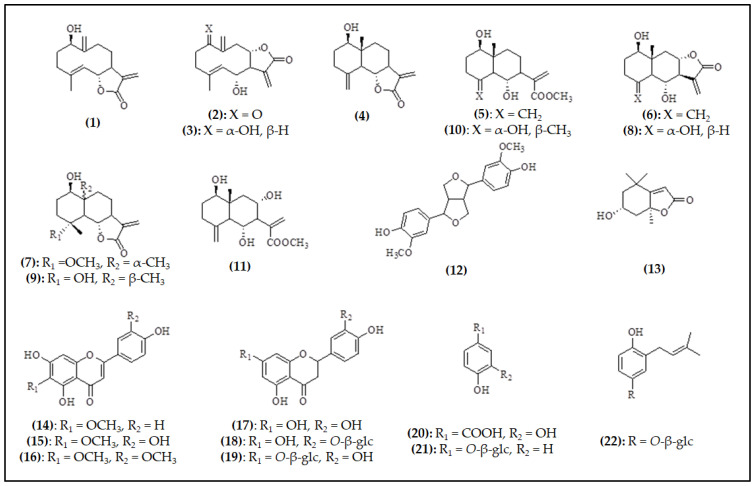
Chemical structures of the compounds isolated from *Staehelina uniflosculosa*.

A total of twenty-two compounds were isolated and identified as sesquiterpene lactones (**1**–**11**); lignans (**12**); norisoprenoids (**13**); as well as flavonoids, flavones (**14**–**16**) and flavanones (**17**–**19**); and simple phenolics: phenolic acid (**20**) and phenolic glucosides (**21**, **22**) (Figure 2). From the aforementioned compounds, only nepetin (6-methoxyluteolin) (**15**), has been reported previously from the *Staehelina* genus [9].

Compounds **1**–**22** were identified as follows: artemorin (**1**) [14], tamirin (**2**) [15], tanachin (**3**) [15], reynosin (**4**) [16], baynol C (**5**) [17], desacetyl-β-cyclopyrethrosin (**6**) [18], 1β-hydroxy-4α-methoxy-5α,7α,6β-eudesm-11(13)-en-6,12-olide (**7**) [19], 1β,4α,6α-trihydroxyeudesm-11-en-8α,12-olide (**8**) [20], 1β-hydroxy-arbusculin A (**9**) [21], methyl-1β,4α,6α-trihydroxy-5α,7αH-eudesm-11(13)-en-12-oate (**10**) [22], methyl-1β,6α,8α-trihydroxy-5α,7αH-eudesma-4(15),11(13)-dien-12-oate (**11**) [23], pinoresinol (**12**) [24], loliolide (**13**) [25], hispidulin (**14**) [26], nepetin (**15**) [27], jaceosidin (**16**) [28], eriodictyol (**17**) [29], eriodictyol-3′-O-glucopyranoside (**18**) [30], eriodictyol-7-O-β-D-glucuronide (**19**) [31], protocatechuic acid (**20**) [32], arbutin (**21**) [33] and nebrodenside A (**22**) [34].

### 2.2. Biological Activities

The isolated natural products were tested in different bioassays to investigate the inhibition of acetylocholinesterase, that of soybean LOX (Lipoxygenase) and their antioxidant activities using the following: (i) the DPPH scavenging activity, with respect to the standard nordihydroguaiaretic acid (NDGA), vitamin C and Trolox and spectrophotometry, and (ii) the interaction with the water-soluble azo compound 2,2′-azobis (2-amidinopropane) dihydrochloride (AAPH), used as a source of peroxyl radicals, and 6-hydroxy-2,5,7,8-tetramethylchroman-2-carboxylic acid (Trolox) as a reference compound (Table 1).

The isolated compounds were tested in vitro as acetylocholinesterase and soybean lipoxygenase inhibitors as well as lipid peroxidation inhibitors (Table 2).

### 2.3. In Silico Analysis of the Secondary Metabolites Isolated from S. uniflosculosa

Virtual or in silico screening is the process of searching through large chemical databases using computational chemistry techniques to identify possible new drug candidates. There are many different virtual screening techniques available, ranging from simple methods that check for the presence or absence of specific substructures or matches in calculated chemical characteristics to intricate virtual docking procedures meant to fit potential ligand molecules into the target receptor site [35]. Table 3 lists the outcomes of the secondary metabolites that were isolated from *S. uniflosculosa*.

## 3. Discussion

### 3.1. Chemotaxonomic Significance of Isolation of Compounds

To the best of our knowledge, this is the first phytochemical study report on *S. uniflosculosa*. Most of the isolated flavonoids (**14**–**19**) and phenolics (**20** and **22**) from *S. uniflosculosa* are very common secondary metabolites. Some of these substances have been isolated from a wide variety of different plants and different families. The flavanone glucoside (**18**) is an exception. This is the first report of its presence in Asteraceae and the fifth instance of its isolation as natural product. It has been previously detected from four different families: Pinaceae [30], Lauraceae [36], Asclepiadaceae [37] and Rosaceae [38].

In general, from the category of sesquiterpene lactones, substances **7**, 1**0** and **11** have been isolated only once, while substance **9** has been isolated three times. Compound **9**’s first report as natural product was in 1973 from Samek et al. [39], and it was isolated from *Tanacetum vulgare* L. It has also been isolated from *Saussurea lappa* Clarke [21,40] and from *Laurus novocanariensis* Rivas Mart., Lousã, Fern. Prieto, E. Días, J.C. Costa and C. Aguiar [41]. Sesquiterpene lactones have been isolated mainly from the family Asteraceae [4]. Interestingly, compounds **2**, **3**, **6**, **7**, **8** and **11** have been detected only in the plant family of Asteraceae. Furthermore, genera from which the above compounds have been mainly isolated include *Tanacetum* L. [42], *Artemisia* L. [43], *Anthemis* L. [44], *Achillea* L. [45] and *Gonospermum* Less. [46], which belong to the tribe Anthemideae. It is noteworthy that compounds **2**, **3** and **4** have also been isolated according to the bibliography from the zoanthid *Palythoa* aff. *clavata*. Sphenopidae (animalian kingdom) [47,48]. Moreover, sesquiterpenoid lactones **1** and **4** have been isolated from the genus *Magnolia* Plum. ex L. (Magnoliaceae) and *Laurus* L. (Lauraceae) [49,50,51,52].

Previous studies on the genus *Staehelina* indicated the predominance of sesquiterpene lactones, either C-6 or C-8 lactonized, all of them belonging to the subcategory of germacranolides. Specifically, four sesquiterpene lactones were isolated from *S. dubia*, all C-8 lactonized [10], whereas four C-6 lactonized sesquiterpene lactones were found in *S. fruticosa* [11]. According to our research, both lactone types can be isolated from *S. uniflosculosa*.

Regarding compound **22**, it is a rare natural product. Until now, nebrodenside A has been isolated from *Ephedra sinica* Stapf. and *E. nebrodensis* Tineo (Ephedraceae) [34,53], *Phagnalon sordidum* (L.) Rchb. (Asteraceae) [54], *Leontopodium leontopodioides* (Willd.) Beauverd (Asteraceae) [55] and *Dodonaea viscosa* Jacq. (Sapindaceae) [56].

The literature data show that sesquiterpene lactones could be used as chemotaxonomic markers for the species *S. dubia* and *S. fruticosa*. However, the present findings suggest that flavonoids, phenolics or lignans could also be used as markers for the genus *Staehelina*. Additionally, it seems that the two previously investigated species have yielded only germacranolides, a single sesquiterpene lactone class. It is noteworthy that this is the first time that eudesmanolides have been isolated from the genus *Staehelina*. This suggests that the presence of both germacranolides and eudesmanolides could be of taxonomic importance.

The combination of present data with previous investigations suggests that the sesquiterpene lactone moiety is a characteristic of the genus *Staehelina* in spite of the fact that the major components of the species *S. uniflosculosa* are mainly phenolic derivatives.

### 3.2. Biological Activitiy of Isolated Compounds

The compounds exhibited very low antioxidant activity. This low ability might be influenced by the stereochemistry of the molecules (Table 1). Only two compounds, **5** and **22**, interacted strongly with the free radical DPPH and appeared to be equipotent to the standard agent NDGA as well as to Trolox and vitamin C. To the best of our knowledge, there are no available literature data about the antioxidant activity of baynol C (**5**). The result of the antioxidant activity of nebrodenside A (**22**) is in accordance with that reported by Cherchar et al. [54]. In the DPPH assay, the dominant chemical reaction involved is the reduction of the DPPH radical by an electron transfer (ET) from the antioxidant. Particularly effective, such antioxidants are the phenoxide anions from phenolic compounds like catechol and derivatives, such as NDGA. Moreover, the compounds did not present any significant antilipid peroxidation activity on the inhibitor Trolox (Table 2). The isolated compounds did not inhibit significantly the soybean LOX. Eriodictyol 3′-O-β-D-glucoside (**18**) was the only compound with distinct activity (90%) (Table 2).

### 3.3. In Silico Studies of Isolated Compounds

The sesquiterpene lactone bigelovin has been extracted from *Inula hupehensis* (Y. Ling) Y. Ling (Asteraceae) flowers. A panel of cancer cells, including A549 (lung cancer), SGC-7901 (gastric cancer), BEL-7402 (liver cancer), U251 (glioma), B16 (murine melanoma), K562 (leukemia) and U937 (leukemia), were shown to be effectively inhibited in their proliferation by this natural substance. The above-mentioned natural product bigelovin was identified as a selective nuclear receptor retinoid X receptor (RXRα) agonist [57]. Novel RXR modulator chemotypes were inspired by the discovery of the natural substance valerenic acid (sesquterpenoids), which was identified using virtual screening as an RXRβ agonist with a functional preference over RXRα and RXRγ [58]. The above-mentioned sesquiterpenoids are reported as nuclear receptor ligands according to the literature. The results listed in Table 3 indicate that almost all the isolated sesquiterpene lactones from *S. uniflosculosa* showed promising bioactivity scores as nuclear receptor ligands for drug targets using Molinspiration software (compounds **1**–**2** and **5**–**11**).

## 4. Materials and Methods

### 4.1. Plant Material and Isolation of Secondary Metabolites

#### 4.1.1. Plant Material

The air-dried aerial parts of *S. uniflosculosa* (0.65 kg) were finely ground and extracted repeatedly at room temperature with petroleum ether/diethyl ether/methanol (1:1:1). The extract was washed with brine, the aqueous layer was re-extracted with ethyl acetate (EtOAc) and then the organic layer was dried over Na_2_SO_4_ and concentrated under reduced pressure to obtain a viscous mass (~14.8 g).

#### 4.1.2. Isolation of Compounds

The ethyl acetate extract was prefractionated by Vacuum Liquid Chromatography (VLC) on silica gel (10.0 × 7.0 cm), using n-hexane-EtOAc-Me_2_CO-MeOH mixtures of increasing polarity as eluents to give twelve fractions of 300 mL each (A-M). Fractions D (1.23 g), E (504.6 mg), F (561.8 mg), G (193.8 mg), H (279.4 mg), I (3.1 g) and K (5.55 g) were subjected to further chromatographic separations as described below.

Fraction D (eluted with n-hexane/EtOAc 25:75) was subjected to CC on silica gel with n-hexane-CH_2_Cl_2_-MeOH to give eleven fractions (DA-DL). Fraction DK (eluted with CH_2_Cl_2_-MeOH 80:20, 28.7 mg) was refractionated over Sephadex LH-20 using MeOH as the eluent and afforded two fractions (DKA and DKB). Fraction DKB yielded compound (**14**) (1.0 mg).

Fraction E (eluted with EtOAc 100%) was subjected to CC on silica gel (n-hexane–CH_2_Cl_2_-MeOH) to give eleven fractions (EA-EL). Fraction EB (eluted with CH_2_Cl_2_-MeOH 99:1, 63.5 mg) was further fractionated by semipreparative HPLC (MeOH-H_2_O, 1:1) and allowed for the isolation of compounds (**1**) (t_R_ = 29.45 min, 14.6 mg) and (**4**) (t_R_ = 39.55 min, 18.7 mg). Fraction ED (eluted with CH_2_Cl_2_-MeOH 98.5:1.5, 26.1 mg) was further fractionated by semipreparative HPLC (MeOH-H_2_O, 3:2) and allowed for the isolation of compounds (**12**) (t_R_ = 12.64 min, 1.0 mg), (**1**) (t_R_ = 13.43 min, 4.0 mg), (**4** and **5**) (t_R_ = 15.86 min, 4.1 mg) and (**16**) (t_R_ = 17.81 min, 1.5 mg). Fraction EE (eluted with CH_2_Cl_2_-MeOH 98:2, 43.2 mg) was further fractionated by semipreparative HPLC (MeOH-H_2_O, 1:1), giving compounds (**2**) (t_R_ = 17.17 min, 3.8 mg), (**13**) (t_R_ = 18.63 min, 3.5 mg), (**5**) (t_R_ = 27.54 min, 3.1 mg) and (**1**) (t_R_ = 30.13 min, 3.3 mg). Fraction EI (eluted with CH_2_Cl_2_-MeOH 95:5, 27.0 mg) was further separated by preparative TLC (CH_3_COOH 15%, Cellulose plates Merck, Art. 5716), yielding compounds (**15**) (Rf = 0.06, 22.3 mg) and (**17**) (Rf = 0.29, 2.8 mg).

Fraction F (eluted with EtOAc-Me_2_CO, 90:10) was submitted to CC on silica gel (n-hexane–CH_2_Cl_2_-MeOH) and yielded thirteen fractions (FA-FM). Fraction FD (eluted with CH_2_Cl_2_-MeOH 98:2, 30.2 mg) was further fractionated by semipreparative HPLC (MeOH-H_2_O, 1:1) and allowed for the isolation of compounds (**2**) (t_R_ = 16.83 min, 1.4 mg) and (**1**) (t_R_ = 28.78 min, 6.0 mg). Fraction FF (eluted with CH_2_Cl_2_-MeOH 97:3, 81.5 mg) was further fractionated by semipreparative HPLC (MeOH-H_2_O, 1:1), yielding compounds (**3**) (t_R_ = 14.08 min, 6.0 mg), (**6**) (t_R_ = 15.72 min, 20.1 mg) and (**7**) (t_R_ = 25.65 min, 4.2 mg). Fraction FG (eluted with CH_2_Cl_2_-MeOH 96:4, 125.9 mg) was further fractionated by semipreparative HPLC (MeOH-H_2_O, 1:1) and allowed for the isolation of compounds (**3**) (t_R_ = 13.37 min, 29.5 mg) and (**9**) (t_R_ = 18.21 min, 4.1 mg). Fractions FK (eluted with CH_2_Cl_2_-MeOH 80:20, 15.6 mg) and FL (eluted with CH_2_Cl_2_-MeOH 50:50, 8.9 mg) were further separated by preparative TLC (CH_3_COOH 15%, Cellulose plates Merck, Art. 5716), yielding compounds (**15**) (Rf = 0.04, 1.6 mg) and (**20**) (Rf = 0.58, 2.0 mg).

Fraction G (eluted with EtOAc-Me_2_CO 75:25) was submitted to CC on silica gel (n-hexane–CH_2_Cl_2_-MeOH) and yielded fourteen fractions (GA-GO). Fraction GD (eluted with CH_2_Cl_2_-MeOH 98:2, 18.4 mg) was further fractionated by semipreparative HPLC (MeOH-H_2_O, 1:1) and allowed the isolation of compound (**7**) (t_R_ = 24.18 min, 4.2 mg). Fraction GE (eluted with CH_2_Cl_2_-MeOH 97:3, 23.2 mg) was further fractionated by semipreparative HPLC (MeOH-H_2_O, 1:1) and allowed for the isolation of compound (**10**) (t_R_ = 17.29 min, 7.1 mg). Fraction GG (eluted with CH_2_Cl_2_-MeOH 94:6, 51.8 mg) was further fractionated by semipreparative HPLC (MeOH-H_2_O, 1:1) and allowed for the isolation of compound (**8**) (t_R_ = 13.56 min, 5.2 mg).

Fraction I (eluted with MeOH 100%) was submitted to CC on silica gel (n-hexane–CH_2_Cl_2_-MeOH-H_2_O) and yielded twenty-three fractions (IA-IX). Fraction IP (eluted with CH_2_Cl_2_-MeOH 82:18) was identified as compound (**21**) (1.07 g). Fraction II (eluted with CH_2_Cl_2_-MeOH 93:7, 34.9 mg) was further fractionated by semipreparative HPLC (MeOH-H_2_O, 1:1) and allowed the isolation of compound (**11**) (t_R_ = 12.53 min, 2.2 mg). Further purification of IO by CC on silica gel (504.6 mg, eluted with CH_2_Cl_2_-MeOH 88:12 to 86:14) yielded twelve fractions. Fractions IOF and IOH were identified as compounds (**18**) (eluted with CH_2_Cl_2_-MeOH 90:10, 81.2 mg) and (**21**) (eluted with CH_2_Cl_2_-MeOH 85:15, 30.5 mg), respectively. Fraction IL (eluted with CH_2_Cl_2_-MeOH 90:10, 76.2 mg) was further fractionated by semipreparative HPLC (MeOH-H_2_O, 1:1) and allowed the isolation of compound (**22**) (t_R_ = 15.97 min, 15.4 mg).

Fraction K (eluted with MeOH 100%) was submitted to CC on silica gel (n-hexane–CH_2_C_l2_-MeOH-H_2_O) and yielded eleven fractions (KA-KL). Fraction KG (eluted with CH_2_Cl_2_-MeOH 60:40, 448.8 mg) was further fractionated by CC on Sephadex LH-20 using MeOH as eluent. Fraction KGF (24.7 mg) was further separated by preparative TLC (CH_2_Cl_2_-MeOH-H_2_O, 80:20:2, Kieselgel plates Merck, Art. 5715) and allowed the isolation of compound (**21**) (Rf = 0.29, 6.6 mg).

All isolated compounds were analyzed by spectroscopic methods (1D and 2D NMR) and their data were compared with those of samples from our collection and/or by a comparison with reported data in the literature.

#### 4.1.3. Chromatographic Techniques

Vacuum Liquid Chromatography (VLC) was carried out on silica gel 60H (Merck Art. 7736) (Merck & Co., Inc., Rahway, NJ, USA), gradient elution with the solvent mixtures indicated in each case; Column chromatography (CC) was carried out on silica gel 60 (Merck Art. 9385), gradient elution with the solvent mixtures indicated in each case and/or Sephadex LH-20 (Cytiva, Morrison, Colorado, USA) using MeOH as eluent; TLC: silica gel (Kieselgel F254, Merck, Art. 5554 and Art. 5715); Cellulose plates, Merck, Art. 5716. Detection on TLC plates: UV light (absorbance: 254 and 366 nm), vanillin–H_2_SO_4_ spray reagent on silica gel.

High-Performance Liquid Chromatography (HPLC): Lab Alliance Series III (LabAlliance, Scientific Systems, Inc., 349 N Science Park Rd., State College PA 16803) equipped with software Clarity (version 9.0.) and Shodex RI Detector (Kawasaki, Japan), using C18 ODS1 Spherisorb 10 μm column, 250 mm × 10 mm, Waters (Milford, Massachusetts, USA).

#### 4.1.4. Spectroscopic Data

NMR: The ^1^H NMR and ^13^C NMR spectra were recorded in CDCl_3_ and CD_3_OD using the following: Bruker DPX 300 (Billerica, Massachusetts, USA) and Varian V300 (Palo Alto, California, USA) (300.13 MHz for ^1^H-NMR and 75.5 MHz for ^13^C-ΝMR), Bruker AMX 500 (500.1 MHz for ^1^H-NMR and 125.5 MHz for ^13^C-NMR) and Varian V600 (599.833 MHz for ^1^H-NMR) spectrometers. Chemical shifts are reported in δ (ppm) values relative to TMS (7.26 ppm for ^1^H-NMR and 77.0 ppm for ^13^C-NMR for CDCl_3_, and 3.31 ppm for ^1^H-NMR and 49.05 ppm for ^13^C-NMR for CD_3_OD) (Tables S1–S27 and Figures S1–S92).

A Perkin Elmer Lambda 20 UV–Vis spectrophotometer (New York, NY, USA) was used for the radical scavenging activity experiments.

### 4.2. Biological Activity Studies

#### 4.2.1. Chemicals

1,1-Diphenyl-2-picrylhydrazyl (DPPH), Lipoxygenase (1.13.11.12) type I-B (Soybean), linoleic acid (sodium salt), 99% purity and acetylcholinesterase (AChE) Type VI-S were purchased from Sigma (St. Louis, MO, USA). Nordihydroguaiaretic acid (NDGA), vitamin C and Trolox were purchased from Merck. All other chemicals were of analytical grade.

#### 4.2.2. Determination of the Antioxidant Activity (Reducing Activity) of the Tested Compounds’ (%) Interaction with the Stable Radical 1,1-Diphenyl-picrylhydrazyl

To a solution (1 mL) of DPPH in absolute ethanol (0.1 mM), 0.98 mL absolute ethanol and 20 µL of the compounds (10 mM stock solutions, 100 μM final concentrations) dissolved in DMSO were added. The absorbance was recorded at 517 nm after 20 and 60 min at room temperature. The experiments were repeated at least in triplicate and the standard deviation of absorbance was less than 10% of the mean. NDGA was used under the same experimental conditions as a reference compound [59]. Vitamin C and Trolox were also used for comparison reasons.

#### 4.2.3. Soybean LOX Inhibition Study In Vitro

A LOX inhibitory assay in vitro was accomplished as described previously [60]). The assays were repeated at least in triplicate and the standard deviation of absorbance was less than 10% of the mean. NDGA was used as a standard reference.

#### 4.2.4. Inhibition of Linoleic Acid Peroxidation

For initiating the lipid peroxidation, the free radical AAPH was used. The antilipid peroxidation was accomplished as described previously [59]. The oxidation of linoleic acid sodium salt was monitored at 234 nm. The assays were repeated at least in triplicate. Trolox was used under the same experimental conditions as a reference compound.

#### 4.2.5. Inhibition of Acetylocholinesterase In Vitro

The inhibitory activity was accomplished as described by Liargkova et al. [61]. Several dilutions were made from a 10 mM stock solution for the determination of IC_50_ values. Physostigmine was used as a standard inhibitor.

### 4.3. In Silico Study

SMILES notations of all the isolated secondary metabolites were fed into the online Molinspiration software, version 2011.06 (access on 13 June 2024) (www.molinspiration.com), for the prediction of bioactivity scores for drug targets (GPCR ligands, kinase inhibitors, ion channel modulators, enzymes and nuclear receptors).

## 5. Conclusions

The phytochemical investigation of *Staehelina uniflosculosa* proved that this species is a rich source of sesquiterpene lactones and flavonoids. We extracted and characterized twenty-two known compounds from the aerial parts of *S. uniflosculosa*. In conclusion, a number of compounds belonging to different classes of natural products were isolated from *S. uniflosculosa*. This is the first study leading to the isolation of secondary metabolites from the aerial parts of this species of the genus *Staehelina.* It is noteworthy that this is the first time that eight eudesmanolides have been isolated from the genus *Staehelina*. A number of components exhibited moderate activity in a variety of in vitro bioassays. In light of the present results, an extension of this investigation to the isolation and activity studies on components from the rest of the extracts may lead to the discovery of unknown components as well as new bioactive compounds. In silico analysis of the isolated secondary metabolites of *S. uniflosculosa* shows that compounds **1**–**2** and **5**–**11** exhibit promising bioactivity scores as nuclear receptor ligands for drug targets using Molinspiration software.

## Figures and Tables

**Table 1:** Percentage (%) interaction of compounds with DPPH and their % Inhibition of lipid peroxidation.

Compounds	% DPPH Radical Scavenging Capacity [%] at 100 μM Concentration ^1^	% Inhibition of AAPH ^1^
(**1**) Artemorin	20.0 ± 0.1	No
(**2**) Tamirin	10.2 ± 0.4	No
(**3**) Tanachin	20.0 ± 1.0	No
(**4**) Reynosin	No	No
(**5**) Baynol C	92.0 ± 3.2	32.0 ± 1.2
(**7**)	No	No
(**9**)	30.0 ± 1.4	37.0 ± 1.8
(**10**)	No	No
(**11**)	27.0 ± 0.7	No
(**13**) Loliolide	7.4 ± 0.2	No
(**22**) Nebrodenside A	100.0 ± 2.5	12.0 ± 0.6
**NDGA**	95 ± 2.1	-
**Trolox**	91 ± 1.1	76.0 ± 2.3
**Vitamin C**	94.5 ± 3.6	-

^1^ The values measured were stable after 20 min reaction. Data are means of at least three measurements +/− SD; No: no activity.

**Table 2:** Percentage (%) soybean LOX inhibitory activity and % Inhibition of acetylcholinesterase (AChE) of isolated compounds.

Compounds	% Inhibition of LOX ^1^	% Inhibition of AChE ^1^
(**1**) Artemorin	33.0 ± 0.4	20.0 ± 0.5
(**2**) Tamirin	29.0 ± 0.3	21.0 ± 0.7
(**3**) Tanachin	31.0 ± 0.4	28.0 ± 0.3
(**4**) Reynosin	6.0 ± 0.1	-
(**5**) Baynol C	18.5 ± 0.1	-
(**7**) 1β,4α,6α-trihydroxyeudesm-11-en-8α,12-olide	14.0 ± 0.1	-
(**9**)	1.0 ± 00	-
(**10**)	10.0 ± 0.1	-
(**11**)	42.0 ± 0.8	-
(**12**) Pinoresinol	No	14.0 ± 0.1
(**13**) Loliolide	38.0 ± 1.00	-
(**14**) Hispidulin	8.0 ± 00	14.0 ± 0.5
(**15**) Nepetin	26.0 ± 1.2	32.0 ± 0.7
(**17**) Eriodictyol	22.0 ± 0.6	7.0 ± 0.9
(**18**) Eriodictyol-3′-O-β-D-glc	90.0 ± 2.2	20.0 ± 0.4
(**20**) Protocatechuic acid	15.0 ± 0.7	No
(**21**) Arbutin	18.0 ± 0.2	20.0 ± 1.3
(**22**) Nebrodenside A	10.5 ± 0.2	-
**NDGA**	83 ± 1.1	-
**Physostigmine**	-	76 ± 2.1

^1^ (-): not tested; No: no activity.

**Table 3:** Bioactivity scores for drug targets according to Molinspiration software.

Compound	GPCR Receptor	Ion Channel Modulator	Kinase Inhibitor	Nuclear Receptor Ligand	Protease Inhibitor
(**1**) Artemorin	0.42	0.20	−0.37	1.30	−0.11
(**2**) Tamirin	0.03	−0.10	−0.93	1.08	−0.23
(**3**) Tanachin	0.32	0.14	1.37	−0.43	−0.17
(**4**) Reynosin	−0.04	0.34	0.74	−0.64	−0.03
(**5**) BaynolC	0.05	0.18	−0.56	0.71	−0.08
(**6**)	0.20	0.03	−0.38	1.23	0
(**7**)	0.40	0.28	−0.19	1.06	0.16
(**8**) 1β-hydroxy-arbusculin	0.22	0.03	−0.38	1.09	0.06
(**9**)	0.35	0.33	−0.34	1.15	0.04
(**10**)	0.12	0.22	−0.58	0.70	−0.03
(**11**)	0.08	0.14	−0.46	0.72	0.06
(**12**) Pinoresinol	0.01	−0.26	−0.21	0.02	−0.17
(**13**) Loliolide	−0.45	−0.39	−0.91	−0.04	−0.33
(**14**) Hispidulin	−0.07	−0.22	0.21	0.20	−0.33
(**15**) Nepetin	−0.08	−0.23	0.22	0.17	−0.31
(**16**) Jaceosidin	−0.09	−0.24	0.21	0.14	−0.31
(**17**) Eriodictyol	0.07	−0.20	−0.22	0.46	−0.09
(**18**) Eriodictyol-3′-O-β-D-glc *	0.21	−0.12	−0.07	0.39	0.24
(**19**) Eriodictyol-7-O-β-D-glr *	0.21	−0.09	−0.20	0.45	0.28
(**20**) Protocatechuic acid	−0.88	−0.35	−1.10	−0.58	−1.09
(**21**) Arbutin	0.13	0.06	−0.06	0.16	0.21
(**22**) Nebrodenside A	0.28	0.09	0.02	0.53	0.30

* glc: glucoside; glr: glucuronide.

## Data Availability

All data supporting the results of this study are included in the manuscript, and the datasets are available upon request.

## Supplementary Materials

The following supporting information can be downloaded at: https://www.mdpi.com/article/10.3390/ijms251910586/s1.
