# Supplementary material for: Secondary Metabolites and Their Biological Evaluation from the Aerial Parts of Staehelina uniflosculosa Sibth. & Sm. (Asteraceae)
Source: Int J Mol Sci. 2024 Oct 1;25(19):10586. doi: 10.3390/ijms251910586 (PMC11476517; doi:10.3390/ijms251910586)
Supplement: Supplementary file 1 [file ijms-25-10586-s001.zip › ijms-3227187-supplementary.pdf]

SUPPLEMENTARY MATERIAL

Secondary metabolites and their biological evaluation from the aerial parts of *Staehelina uniflosculosa* Sibth. & Sm. (Asteraceae).

Maria Lazanaki[1,2], George Tsikalas[2], Olga S. Tsiftsoglou[1], Haralambos Katerinopoulos[2], Dimitra Hadjipavlou-Litina[3], Diamanto Lazari[1*]

[1] Laboratory of Pharmacognosy, School of Pharmacy, Faculty of Health Sciences, Aristotle University of Thessaloniki, 54124 Thessaloniki, Greece; dlazari@pharm.auth.gr; olga@tsiftsoglou.gr; mlazanak@yahoo.gr

[2] Department of Chemistry, Division of Organic Chemistry, University of Crete, Heraklion 71003, Crete, Greece; kater@chemistry.uoc.gr; gtsikalas@chemistry.uoc.gr

[3] Department of Pharmaceutical Chemistry, School of Pharmacy, Faculty of Health Sciences, Aristotle University of Thessaloniki, 54124 Thessaloniki, Greece; hadjipav@pharm.auth.gr

* Correspondence: dlazari@pharm.auth.gr

Abstract: Phytochemical investigation of *Staehelina uniflosculosa* Sibth. & Sm. resulted in the isolation of twenty-two natural products: eleven sesquiterpene lactones, artemorin (**1**), tamirin (**2**), tanachin (**3**), reynosin (**4**), baynol C (**5**), desacetyl-β-cyclopyrethrosin (**6**), 1β-hydroxy-4α-methoxy-5α,7α,6β-eudesm-11(13)-en-6,12-olide (**7**), 1β,4α,6α-trihydroxyeudesm-11-en-8α,12-olide (**8**), 1β-hydroxy-arbusculin A (**9**), me-thyl-1β,4α,6α-trihydroxy-5α,7αH-eudesm-11(13)-en-12-oate (**10**) and me-thyl-1β,6α,8α-trihydroxy-5α,7αH-eudesma-4(15),11(13)-dien-12-oate (**11**); one lignan, pinoresinol (**12**); one norisoprenoid, loliolide (**13**); six flavonoids (four genins and two glycosides), hispidulin (**14**), nepetin (**15**), jaceosidin (**16**), eriodictyol (**17**), eriodictyol-3′-O-β-D-glucoside (**18**) and eriodicty-ol-7-O-β-D-glucuronide (**19**); and three phenolic derivatives (one phenolic acid and two phenolic glucosides), protocatechuic acid (**20**), arbutin (**21**) and nebrodenside A (**22**). From the isolated compounds, only nepetin (**15**) has been reported previously from the *Staehelina* genus and, to the best of our knowledge, it is the first time that compound (**18**) has been identified in Asteraceae. A number of these substances were tested for (a) inhibition of lipoxygenase and acetylocholinesterase, (b) their antioxidant activity using the DPPH (1,1-Diphenyl-2-picrylhydrazyl) method or/and (c) inhibition of lipid peroxidation. The tested components exhibited low antioxidant activity with the exception of **5** and **22**, while the effectiveness of these compounds in the inhibition of acetylocholinesterase is limited. Furthermore, Molinspiration, an online computer tool, was used to determine the bioactivity ratings of the isolated secondary metabolites. The compounds' bioactivity ratings for potential therapeutic targets were very promising.

Keywords: Sesquiterpene lactones; flavonoids; nuclear receptor ligand

Table S1: ^{1}H and ^{13}C NMR of compound **1** (CD_3OD, 300MHz).

Position	δ_C (CD_3OD)	C	δ_H (CD_3OD)	Number H	Multiplicity J (Hz)
1	77.9	CH	3.92	1	*m*
2	33.9	CH_2	-	2	-
3	31.5	CH_2	2.33, 1.30	2	*m*
4	144.7	C	-	-	-
5	125.1	CH	5.29	1	*br d* (J=9.6)
6	83.4	CH	4.50	1	*t* (J=9.6)
7	-	CH	2.87	1	*m*
8	26.4	CH_2	2.03	2	*m*
9	38.0	CH_2	2.18	2	*m*
11	141.9	C	-	-	-
12	172.6	C=O	-	-	-
13	119.7	CH_2	6.08	2	*d* (J=3.6)
			5.55		*d* (J=3.3)
14	111.6	CH_2	5.18, 4.91	2	*s*
15	18.4	CH_3	1.73	3	*br s*

Table S2: ^{1}H and ^{13}C NMR of compound **1** ($CDCl_3$, 300MHz).

Position	δ_C ($CDCl_3$)	C	δ_H ($CDCl_3$)	Number H	Multiplicity J (Hz)
1	78.9	CH	3.97	1	*m*
2	32.7	CH_2	-	2	-
3	30.3	CH_2	2.30, 1.25	2	*m*
4	144.3	C	-	-	-
5	127.6	CH	5.25	1	*d* (J=9.8)
6	-	CH	4.40	1	*t* (J=9.8)
7	49.3	CH	2.81	1	*m*
8	-	CH_2	1.97	2	*d* (J=4.8)
9	36.4	CH_2	2.17	2	*d* (J=5.1)
11	140.9	C	-	-	-
12	171.1	C=O	-	-	-
13	118.8	CH_2	6.18	2	*d* (J=3.6)
			5.45		*d* (J=3.0)
14	-	CH_2	5.21, 4.87	2	*br s*
15	18.6	CH_3	1.74	3	*br s*

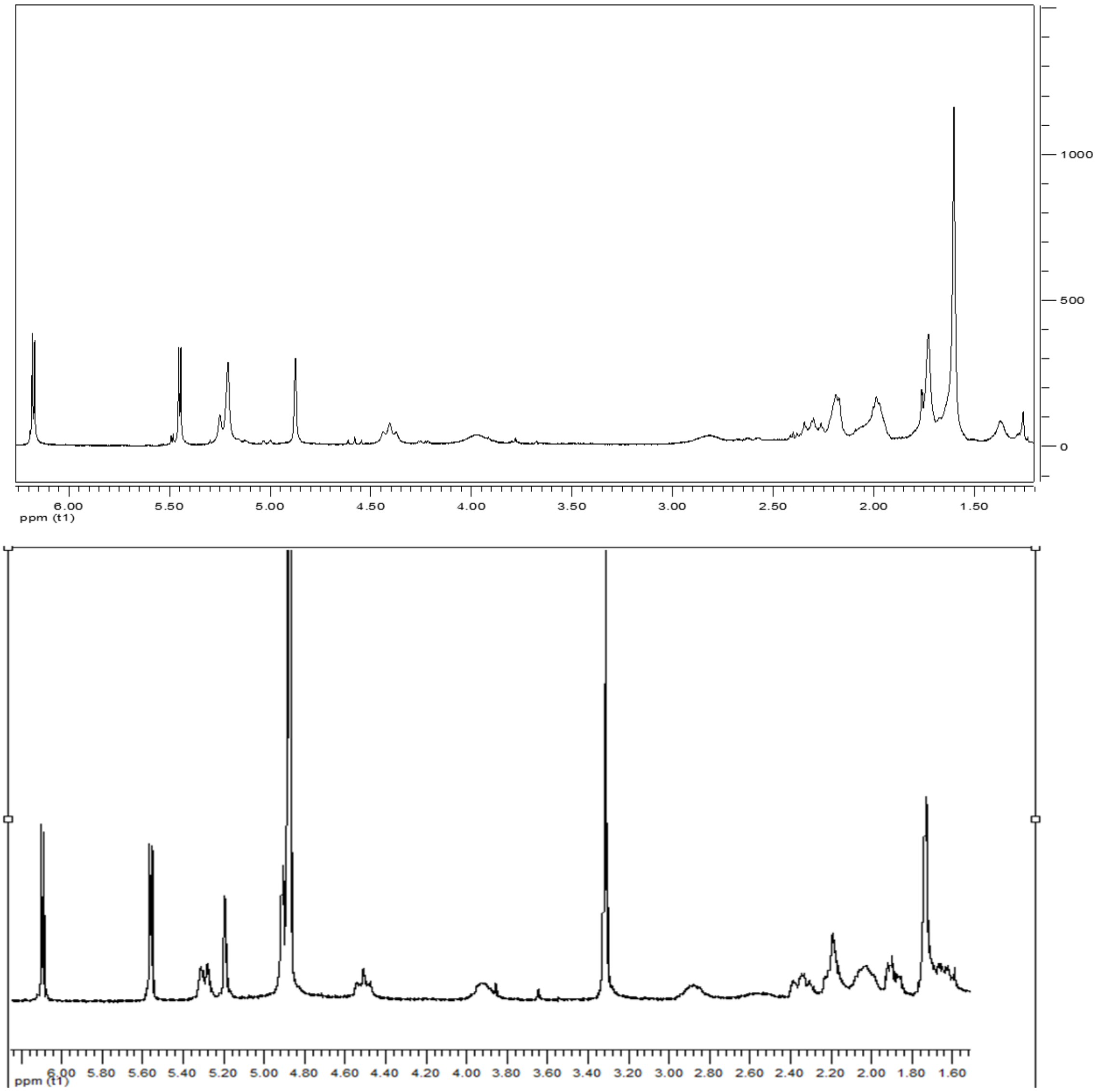

Figure S 1. ^{1}H–NMR spectrum of compound **1** ($CDCl_3$, 300MHz and CD_3OD, 300 MHz).

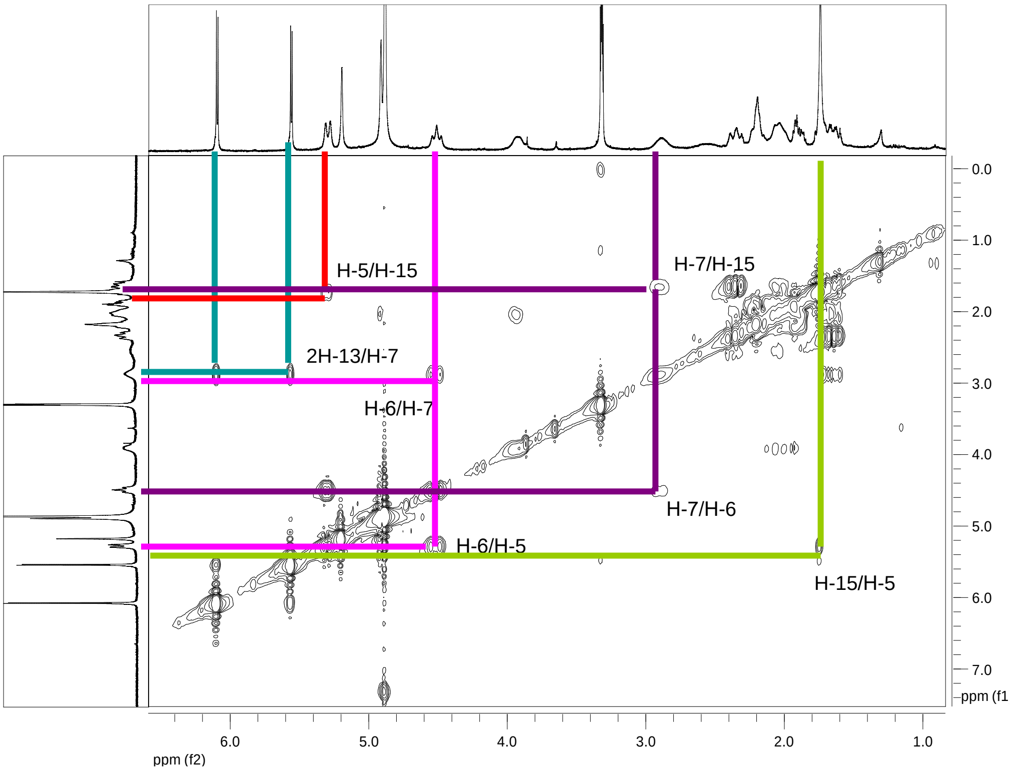

Figure S 2. COSY-NMR spectrum of compound **1** (CD_3OD, 300MHz).

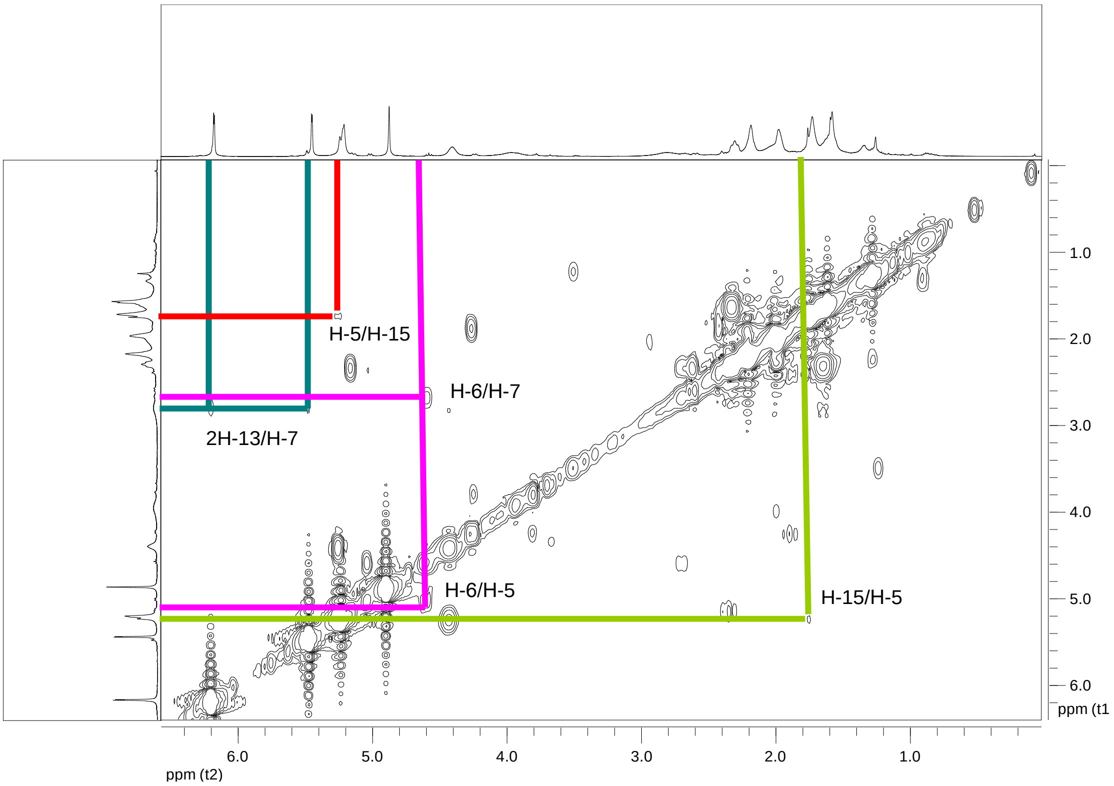

Figure S 3. COSY-NMR spectrum of compound **1** ($CDCl_3$, 300MHz).

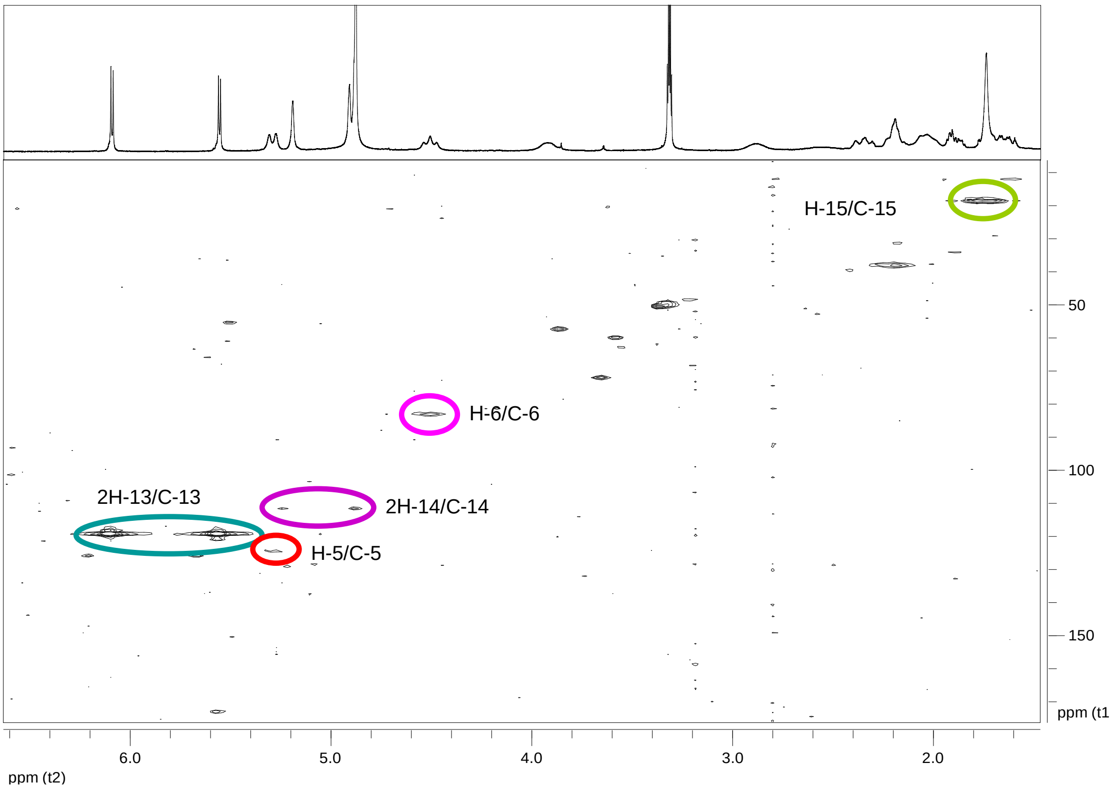

Figure S 4. HMQC-NMR spectrum of compound **1** (CD_3OD, 500MHz).

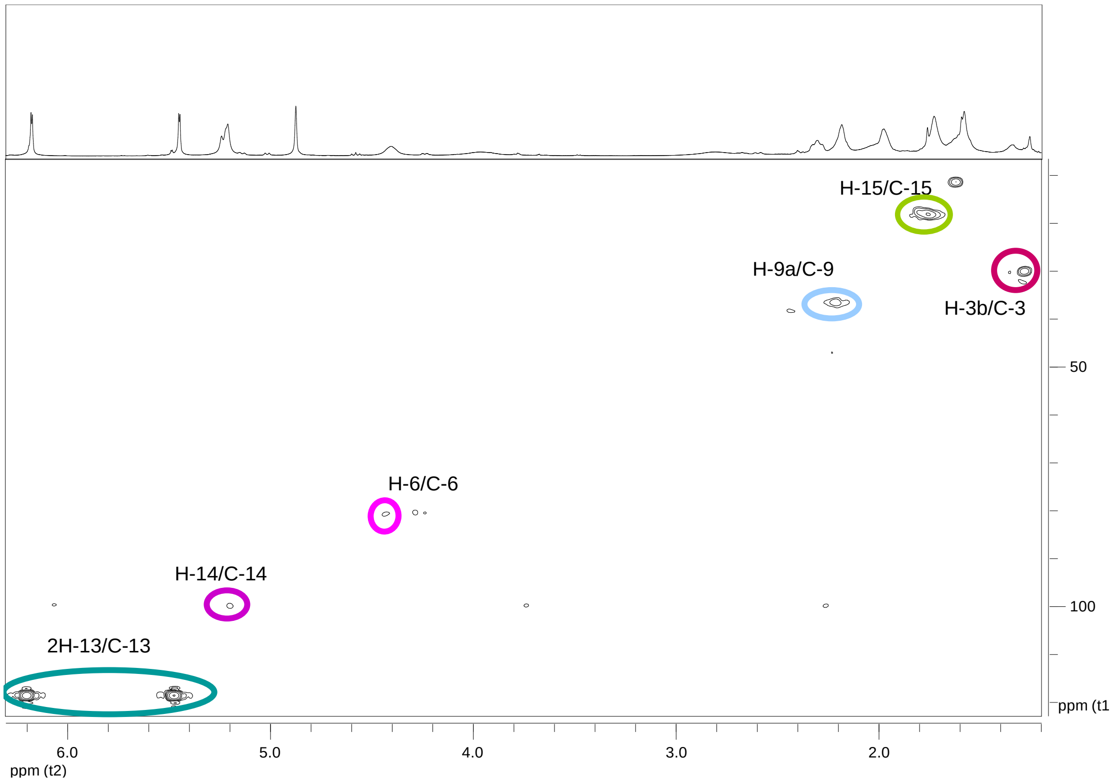

Figure S 5. HMQC-NMR spectrum of compound **1** ($CDCl_3$, 500MHz).

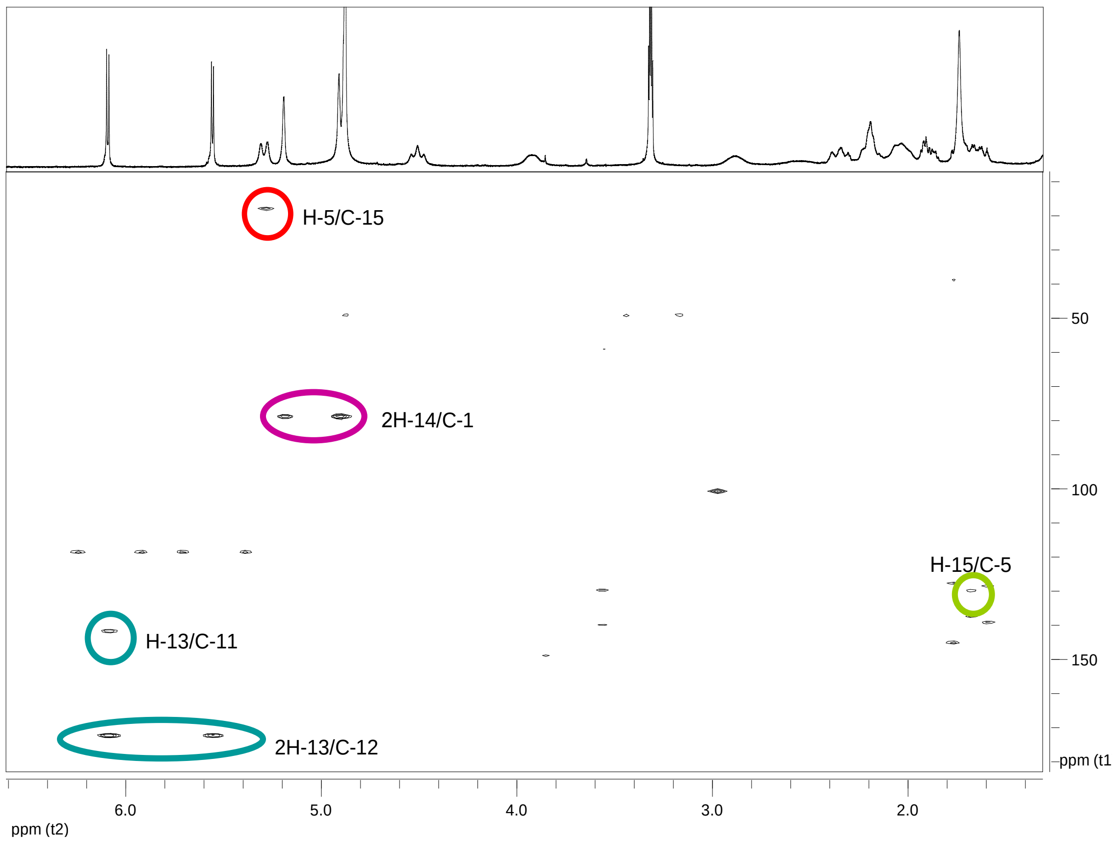

Figure S 6. HMBC-NMR spectrum of compound **1** (CD_3OD, 500MHz).

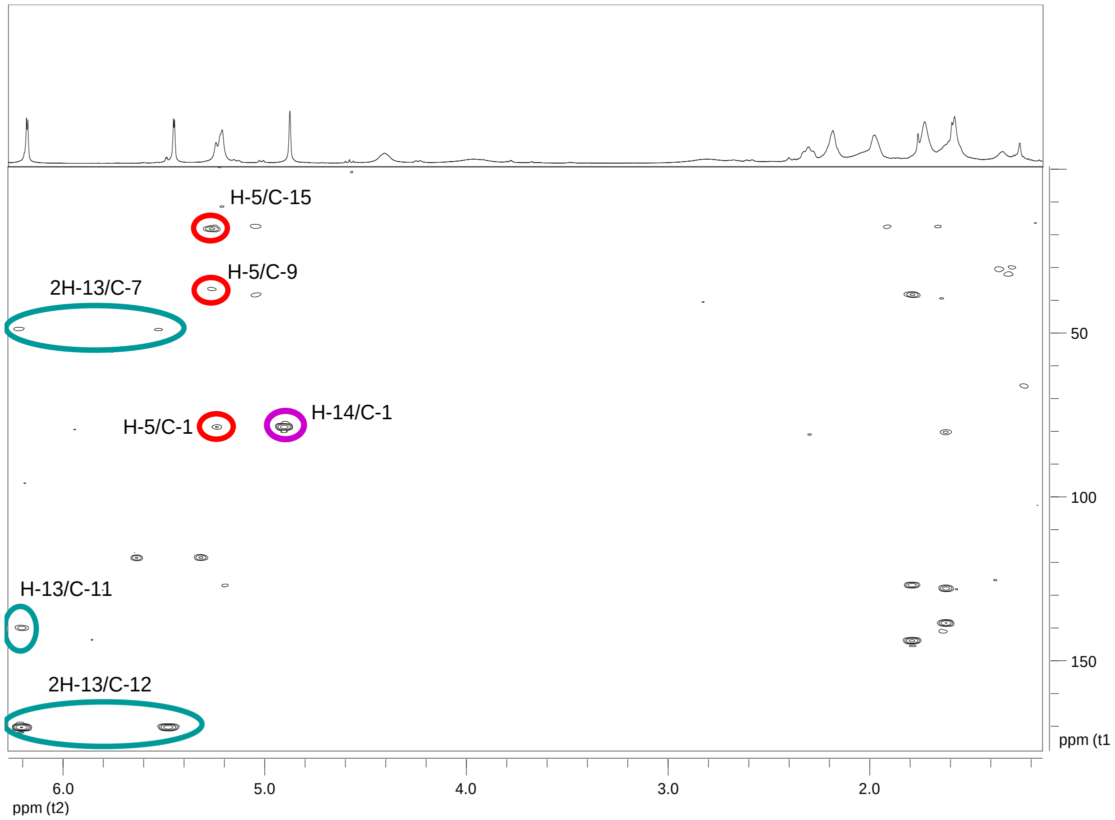

Figure S 7. HMBC-NMR spectrum of compound **1** ($CDCl_3$, 500MHz).

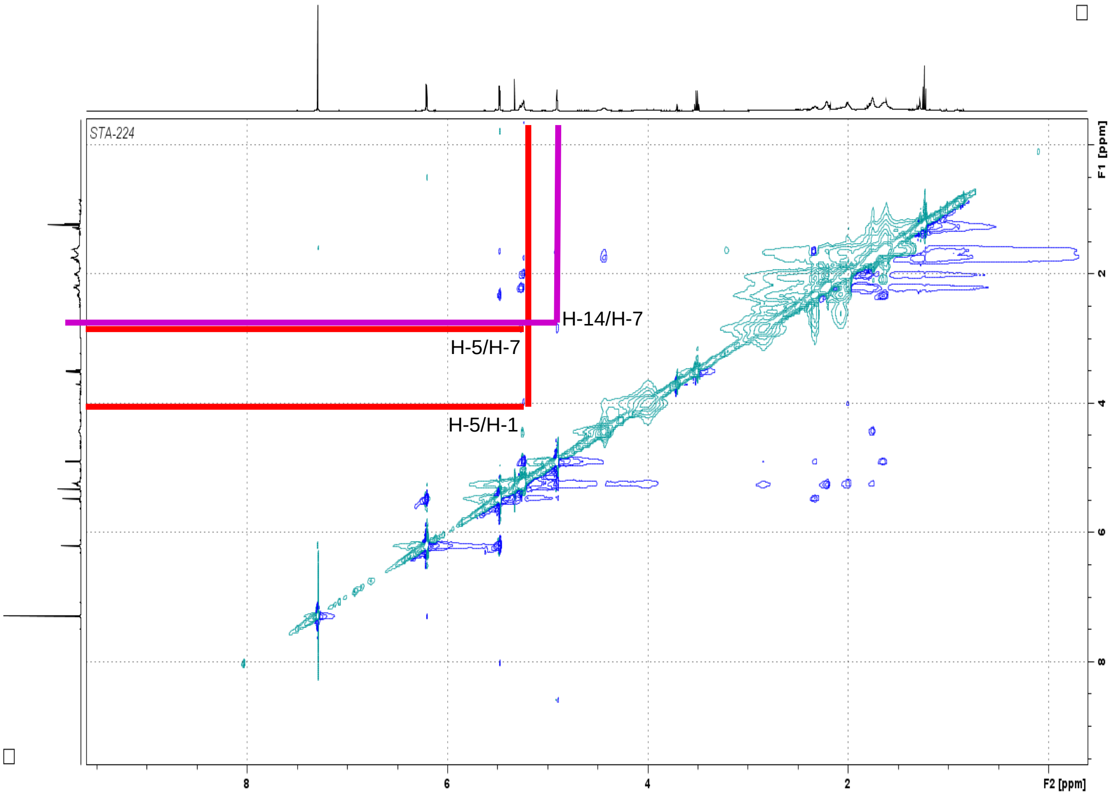

Figure S 8. NOESY-NMR spectrum of compound **1** ($CDCl_3$, 500MHz).

Table S3: ^{1}H and ^{13}C NMR of compound **2** ($CDCl_3$, 300MHz).

Position	δ_C	C	δ_H	Number H	Multiplicity J (Hz)
1	203.2	C	-	-	-
2	36.4	CH_2	3.28	2	*m*
3	35.4	CH_2	2.60-2.37	2	*m*
4	136.0	C	-	-	-
5	131.3	CH	5.07	1	*br d* (J=9.6)
6	70.1	CH	4.16	1	*t* (J=9.6)
7	50.1	CH	2.74	1	*m*
8	76.6	CH	3.95	1	*m*
9	40.1	CH_2	3.40	2	*br d* (J=12.6)
			2.15		*dd* (J=12.0, 12.6)
10	146.3	C	-	-	-
11	136.0	C	-	-	-
12	169.6	C=O	-	-	-
13	126.3	CH_2	6.37	2	*d* (J=3.0)
			6.16		*d* (J=2.4)
14	124.4	CH_2	5.83, 5.78	2	*br s*
15	17.3	CH_3	1.66	3	*br s*

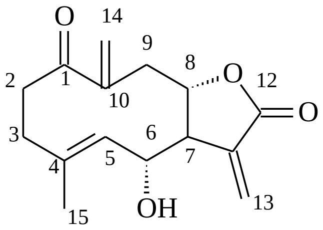

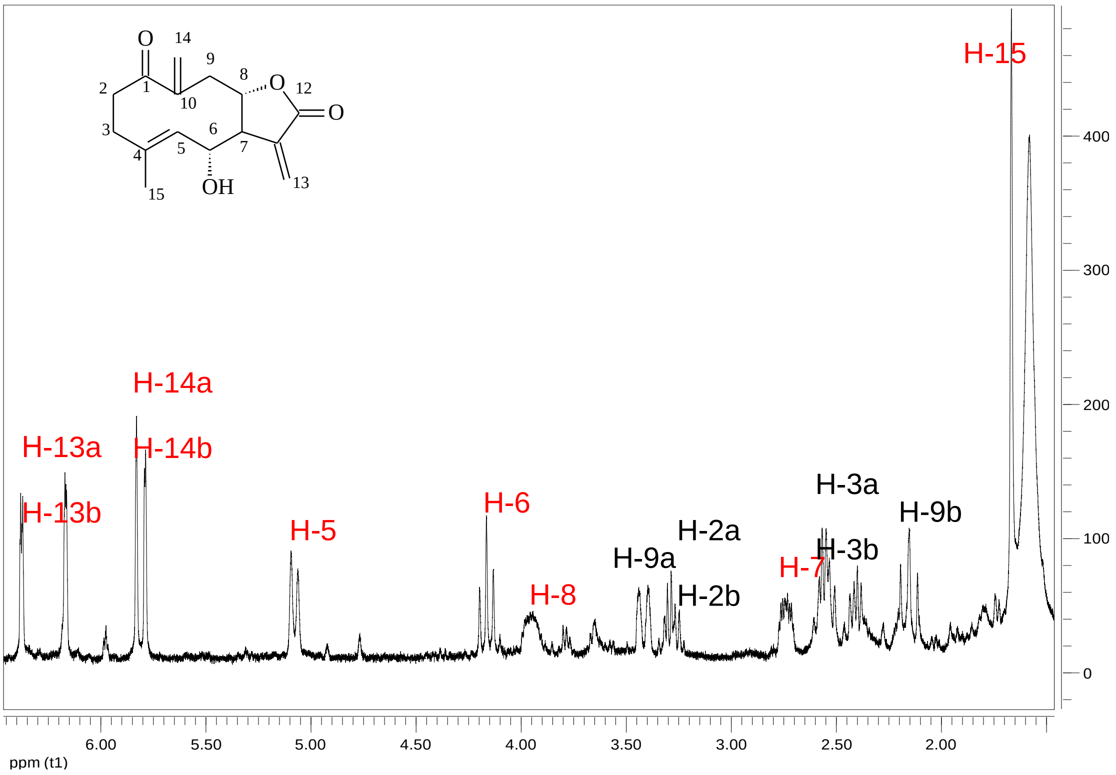

Figure S 9. ^{1}H-NMR spectrum of compound **2** ($CDCl_3$, 500MHz).

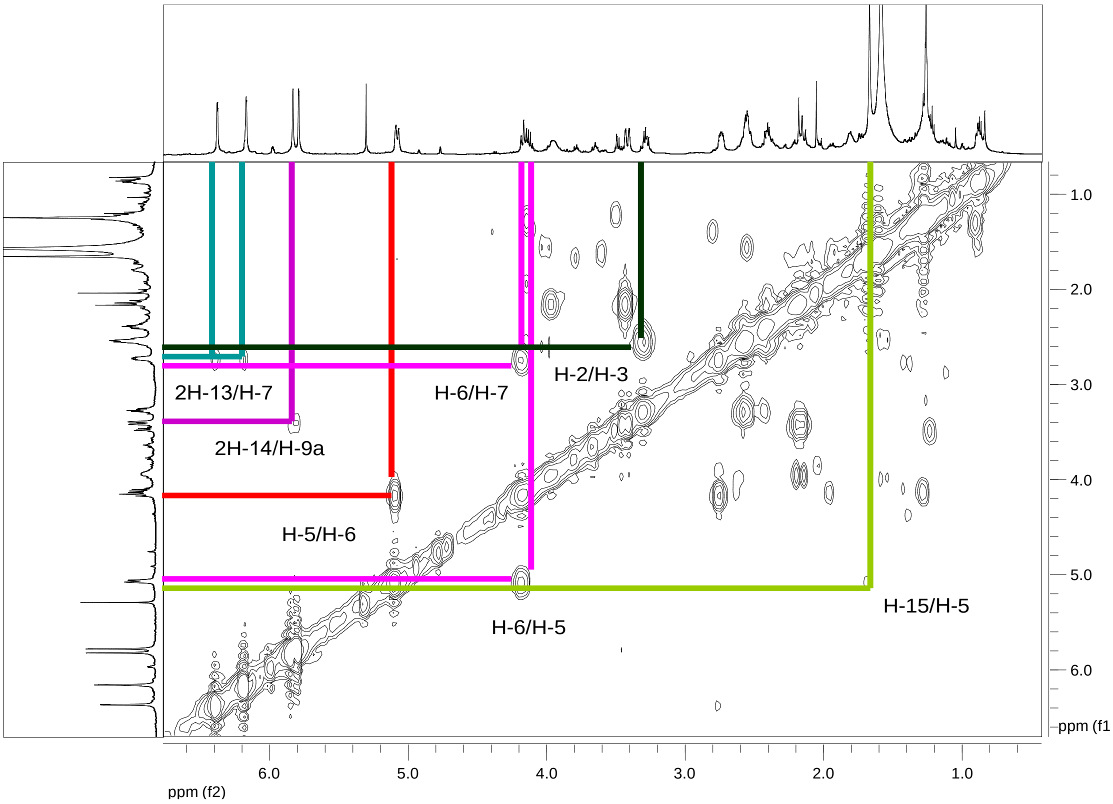

Figure S 10. COSY-NMR spectrum of compound **2** ($CDCl_3$, 300MHz).

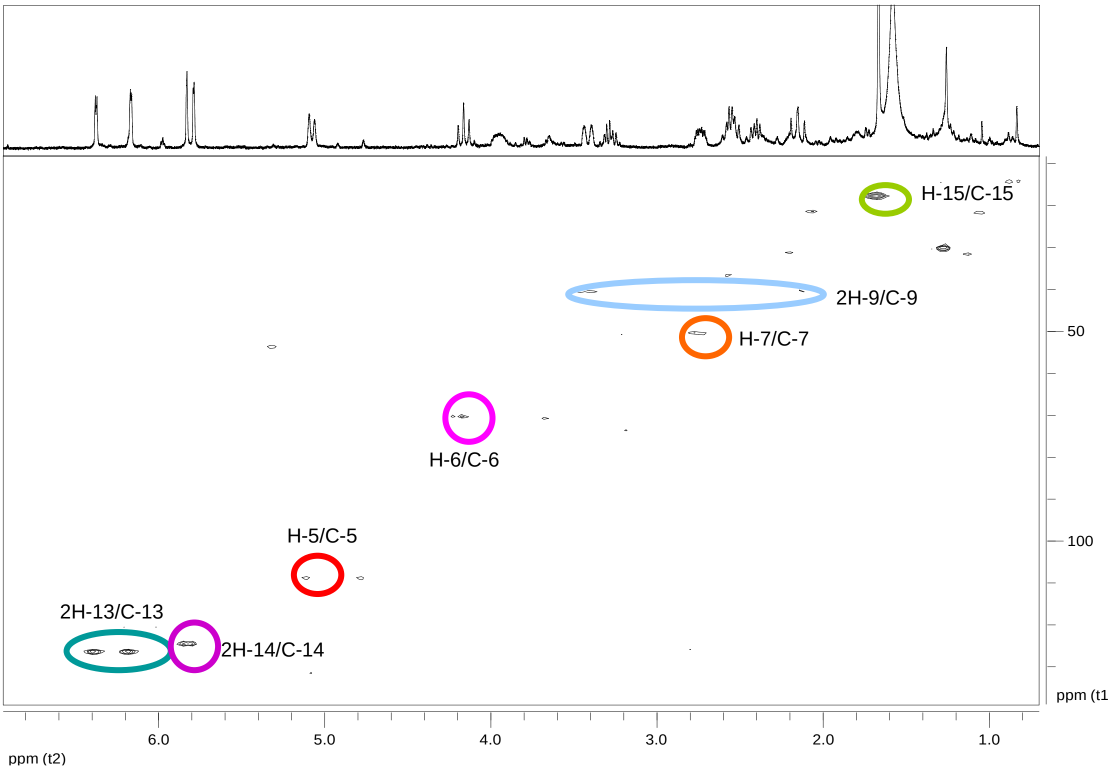

Figure S 11. HMQC-NMR spectrum of compound **2** ($CDCl_3$, 300MHz).

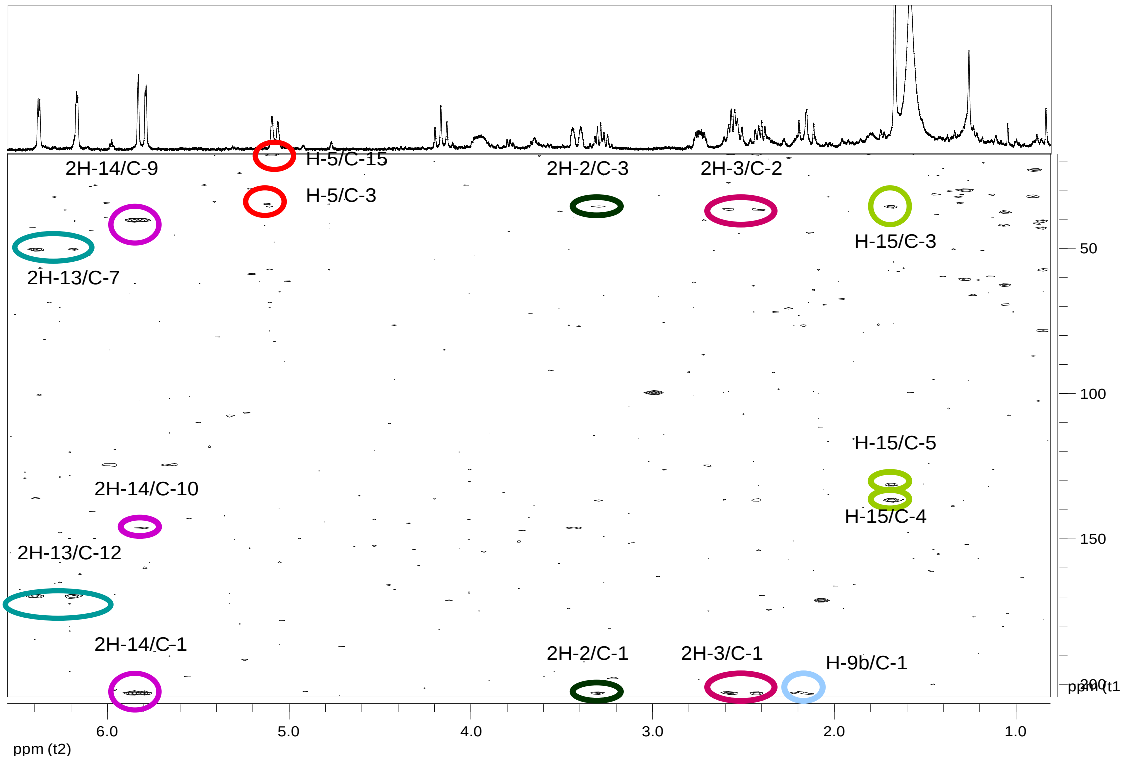

Figure S 12. HMBC-NMR spectrum of compound **2** ($CDCl_3$, 500MHz).

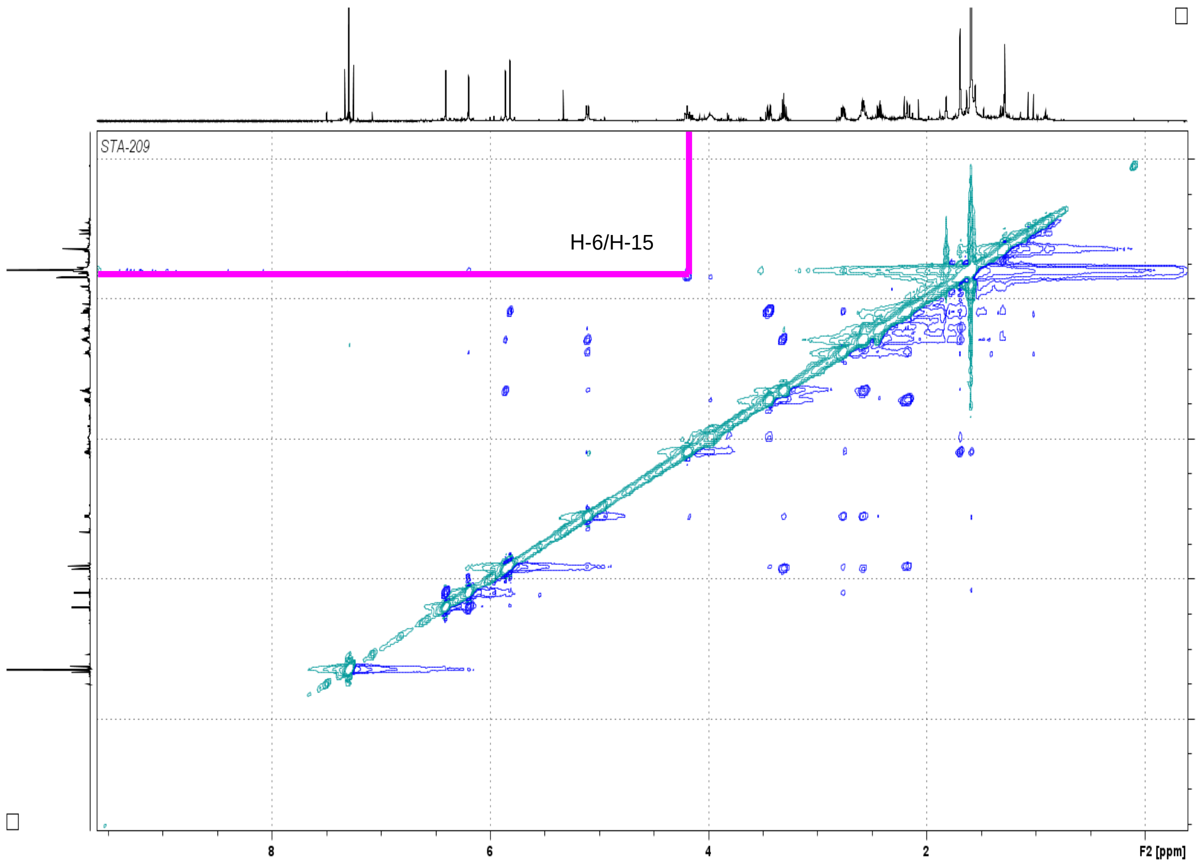

Figure S 13. NOESY-NMR spectrum of compound **2** ($CDCl_3$, 500MHz).

Position	δ_C (CD_3OD)	C	δ_H (CD_3OD)	Number H	Multiplicity J (Hz)
1	71.3	CH	3.84	1	*t* (J=7.5)
2	31.5	CH_2	2.06-2.25	2	*m*
3	35.4	CH_2	2.06-2.25	2	*m*
4	132.8	C	-	-	-
5	135.4	CH	5.04	1	*br d* (J=10.0)
6	70.7	CH	4.23	1	*t* (10.0)
7	53.1	CH	2.81	1	*dddd* (J=3.3, 6.5, 6.0)
8	80.7	CH	4.05	1	*m*
9	42.8	CH_2	2.86	2	*br d* (J=14.0)
			2.42		*dd* (J=10.0, 13.5)
10	147.4	C	-	-	-
11	138.8	C	-	-	-
12	172.1	C=O	-	-	-
13	125.4	CH_2	6.24	2	*d* (J=1.0, 3.0)
			6.19		*d* (J=1.2, 2.5)
14	115.1	CH_2	5.16, 5.12	2	*br s*
15	17.3	CH_3	1.71	3	*s*

Table S4: ^{1}H and ^{13}C NMR of compound **3** (CD_3OD, 500MHz).

Table S5: ^{1}H and ^{13}C NMR of compound **3** ($CDCl_3$, 300MHz).

**Position**	**δ_C ($CDCl_3$)**	**C**	**δ_H ($CDCl_3$)**	**Number H**	**Multiplicity J (Hz)**
1	70.6	CH	3.85	1	*dd* (J=4.8, 10.8)
2	28.1	CH_2	1.99-2.27	2	*m*
3	34.2	CH_2	1.99-2.27	2	*m*
4	136.5	C	-	-	-
5	130.8	CH	5.04	1	*br d* (J=10.5)
6	70.7	CH	4.27	1	*t* (J=10.5)
7	51.6	CH	2.82	1	*dddd* (J=3.3, 6.6, 10.2)
8	78.6	CH	3.97	1	*m*
9	41.7	CH_2	2.96	2	*br d* (J=15.0)
			2.38		*dd* (J=9.9, 14.4)
10	146.6	C	-	-	-
11	136.5	C	-	-	-
12	169.8	C=O	-	-	-
13	125.6	CH_2	6.36	2	*dd* (J=1.2, 3.3)
			6.19		*dd* (J=1.2, 3.0)
14	114.7	CH_2	5.16 , 5.11	2	*br s*
15	17.4	CH_3	1.70	3	*s*

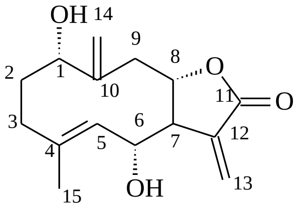

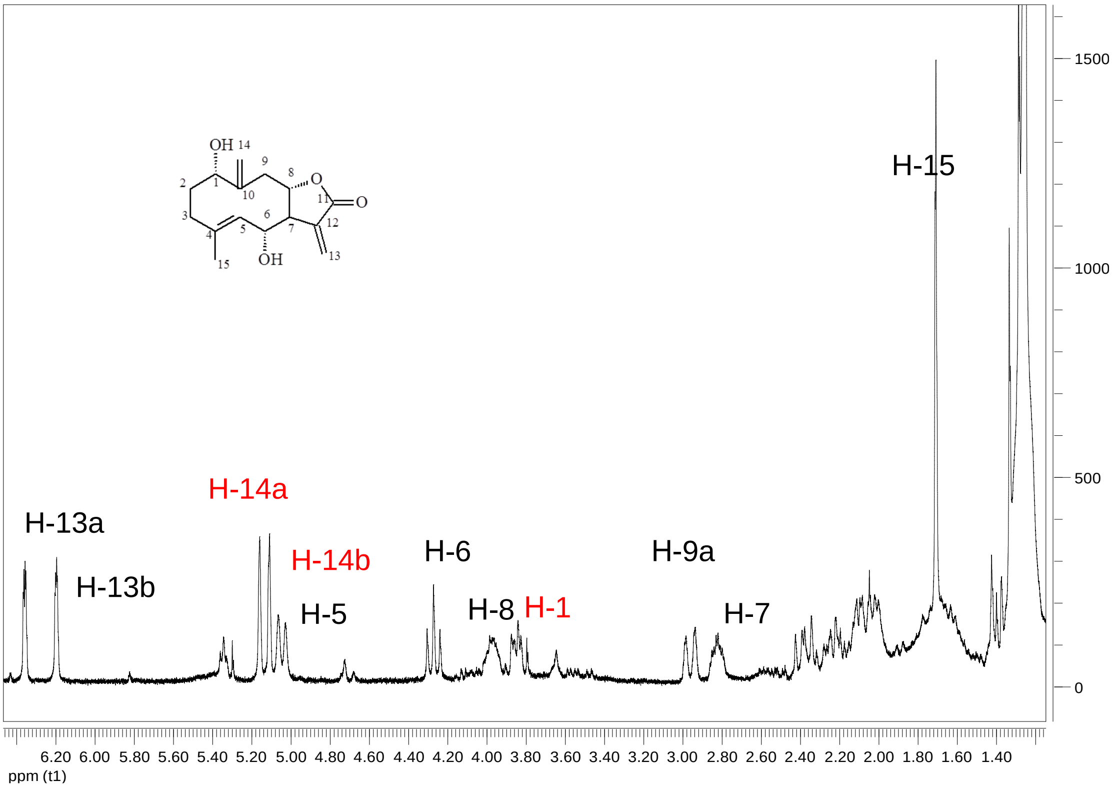

Figure S 14. ^{1}H-NMR spectrum of compound **3** ($CDCl_3$, 300MHz).

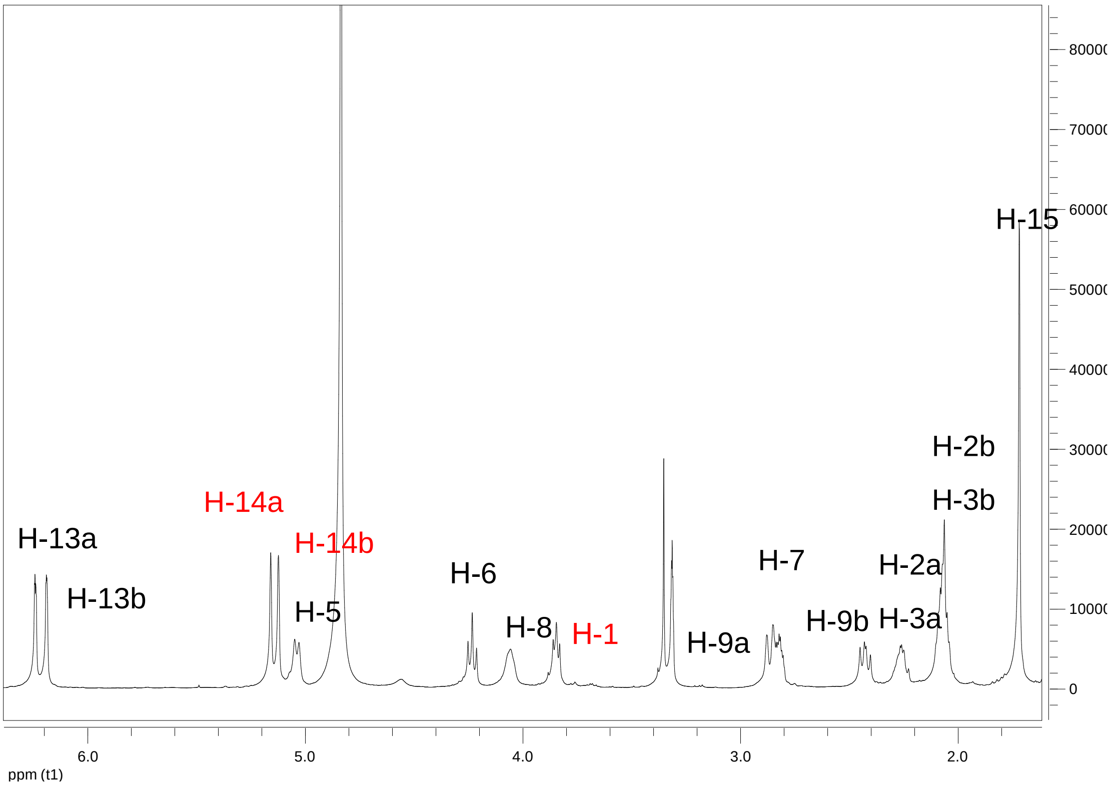

Figure S 15. ^{1}H-NMR spectrum of compound **3** (CD_3OD, 500MHz).

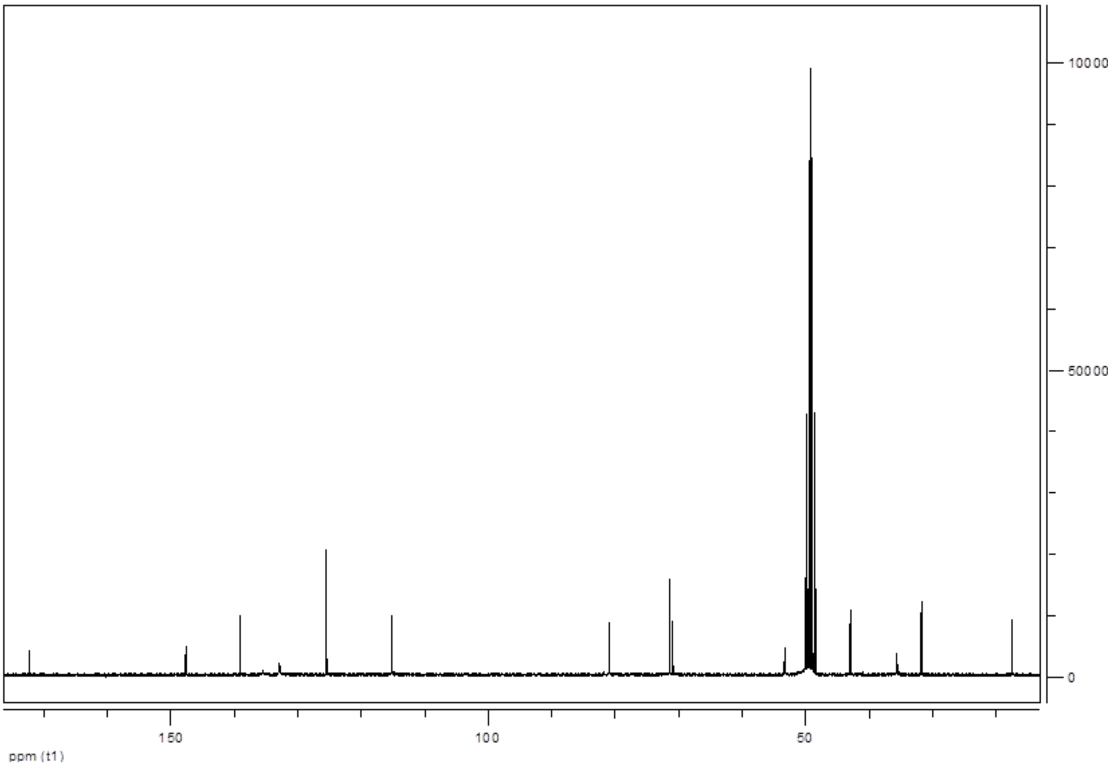

Figure S 16. ^{13}C-NMR spectrum of compound **3** (CD_3OD, 75MHz).

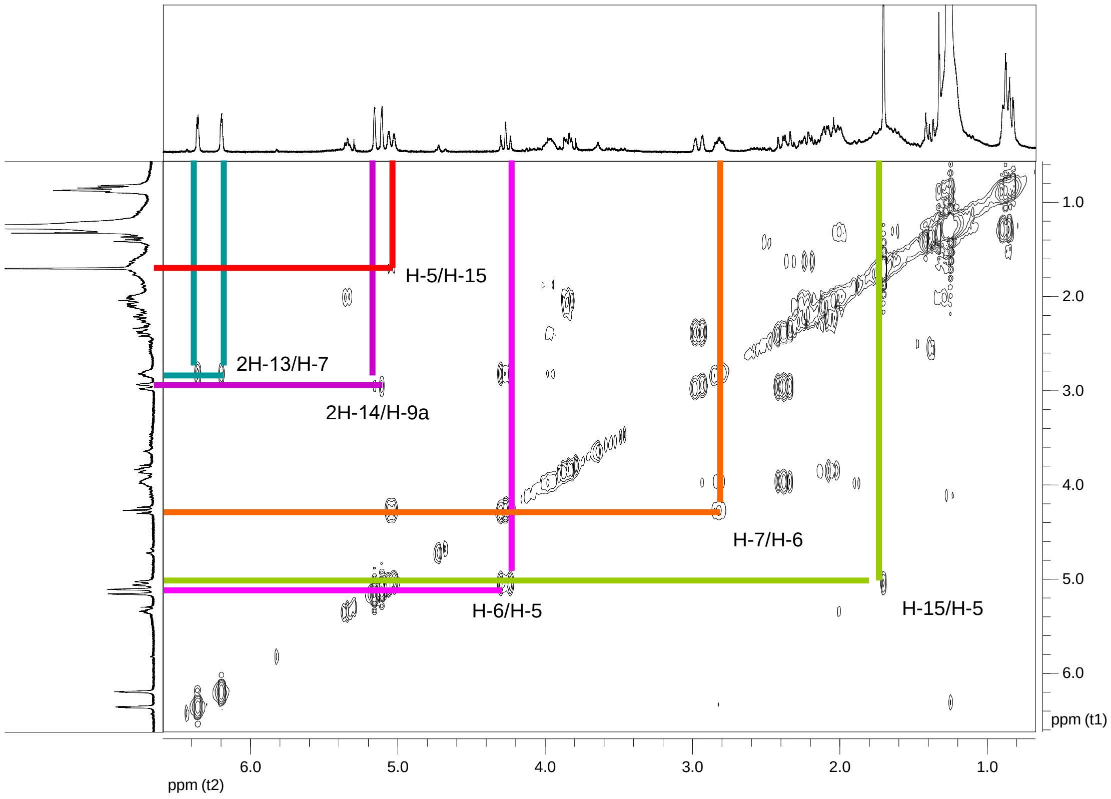

Figure S 17. COSY-NMR spectrum of compound **3** ($CDCl_3$, 300MHz).

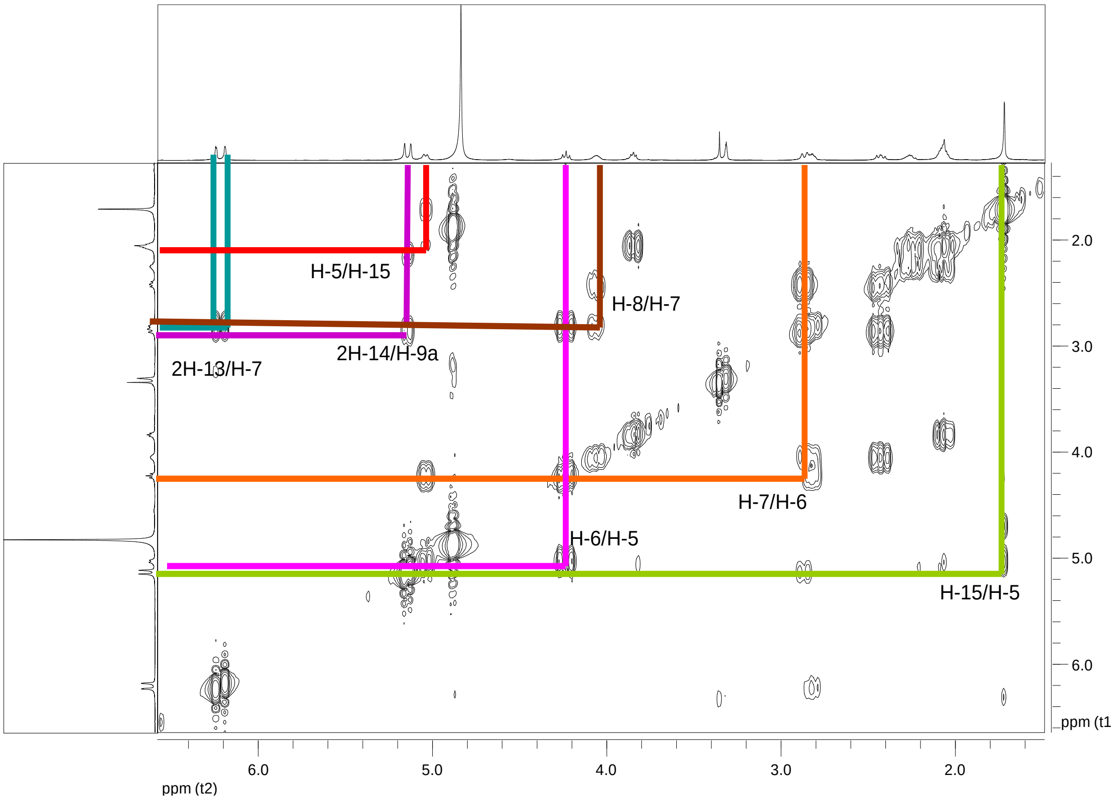

Figure S 18. COSY-NMR spectrum of compound **3** (CD_3OD, 300MHz).

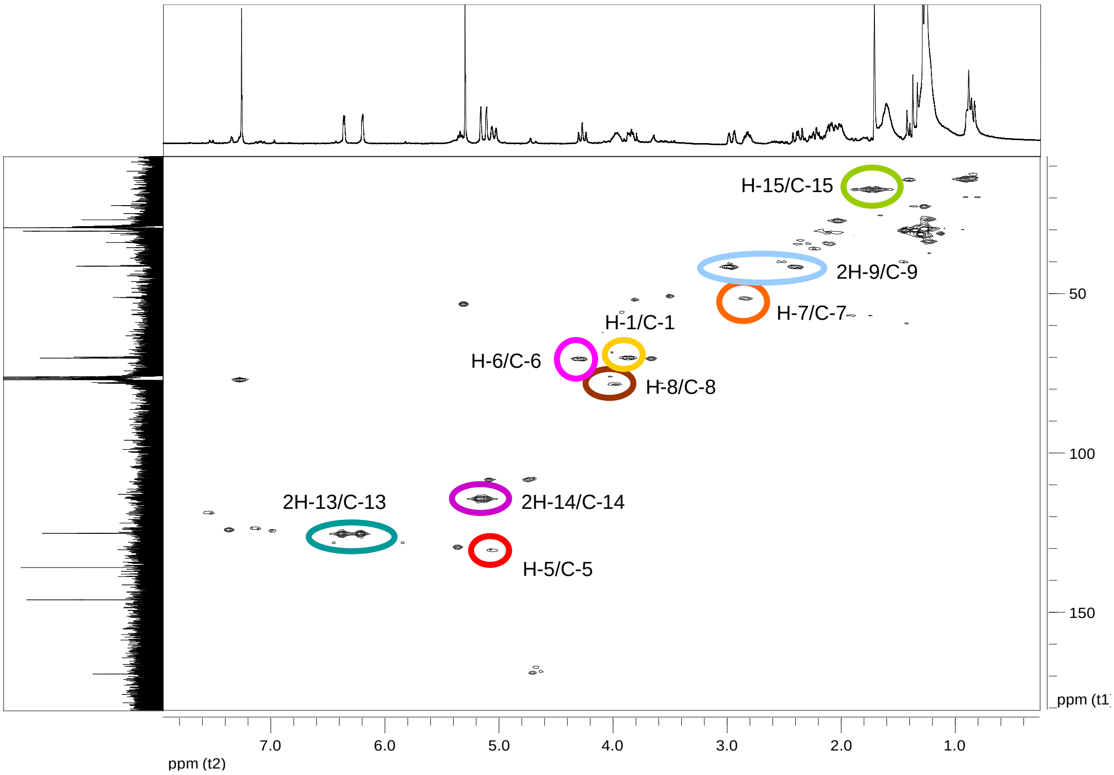

Figure S 19. HMQC-NMR spectrum of compound **3** ($CDCl_3$, 500MHz).

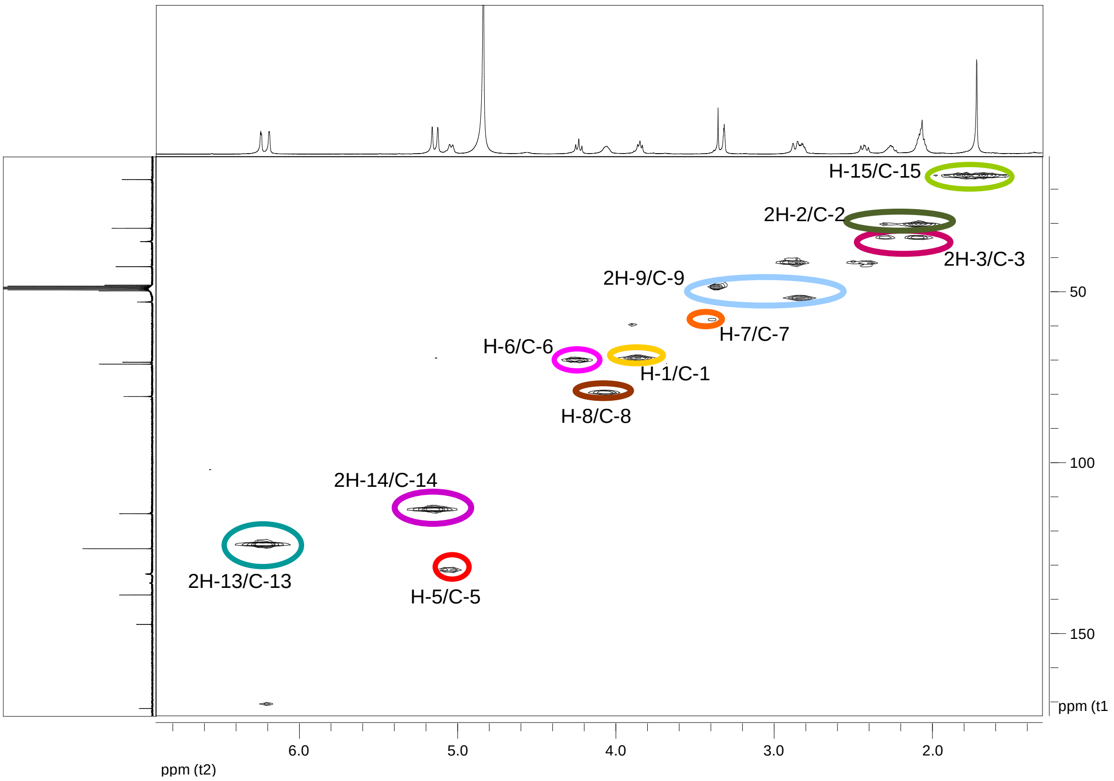

Figure S 20. HMQC-NMR spectrum of compound **3** (CD_3OD, 500MHz).

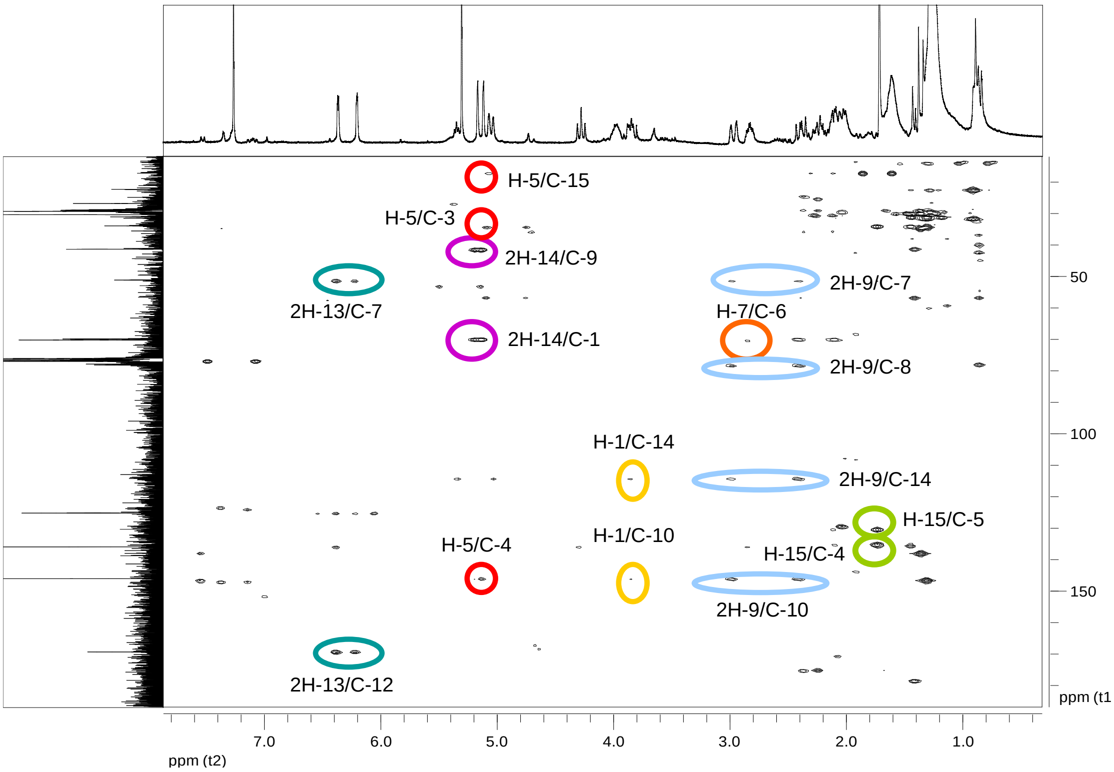

Figure S 21. HMBC-NMR spectrum of compound **3** ($CDCl_3$, 500MHz).

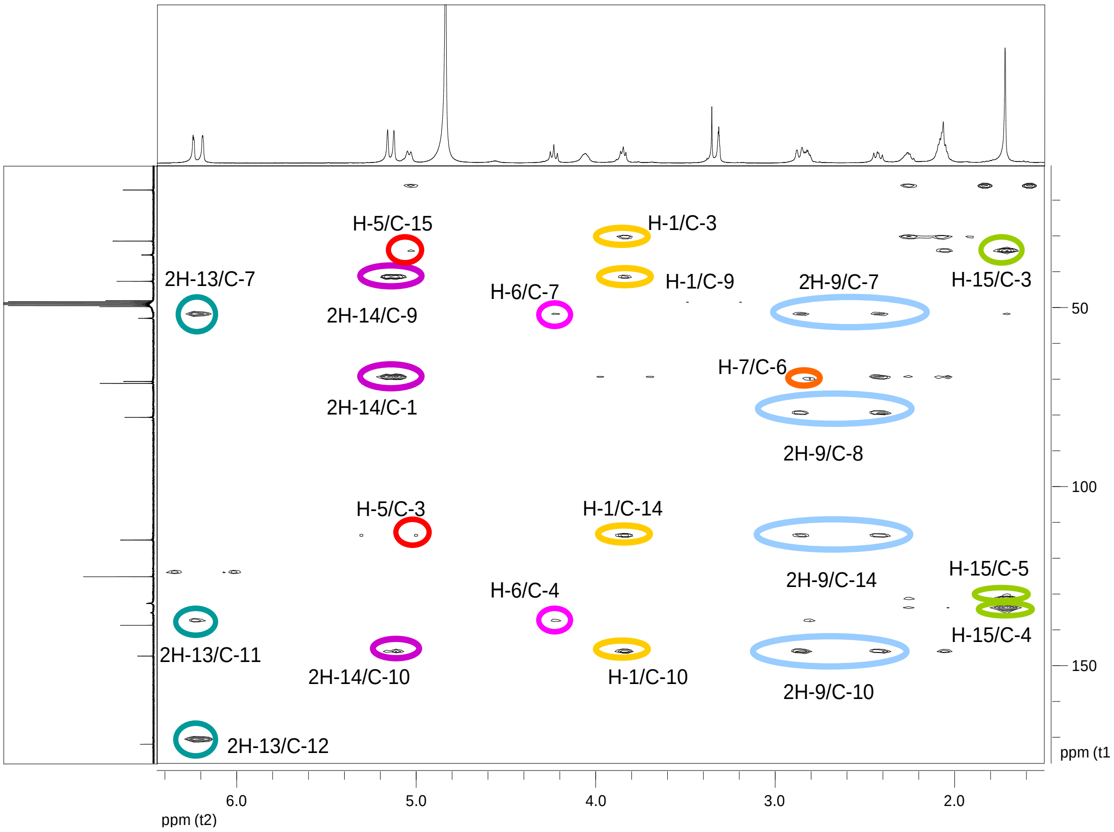

Figure S 22. HMBC-NMR spectrum of compound **3** (CD_3OD, 500MHz).

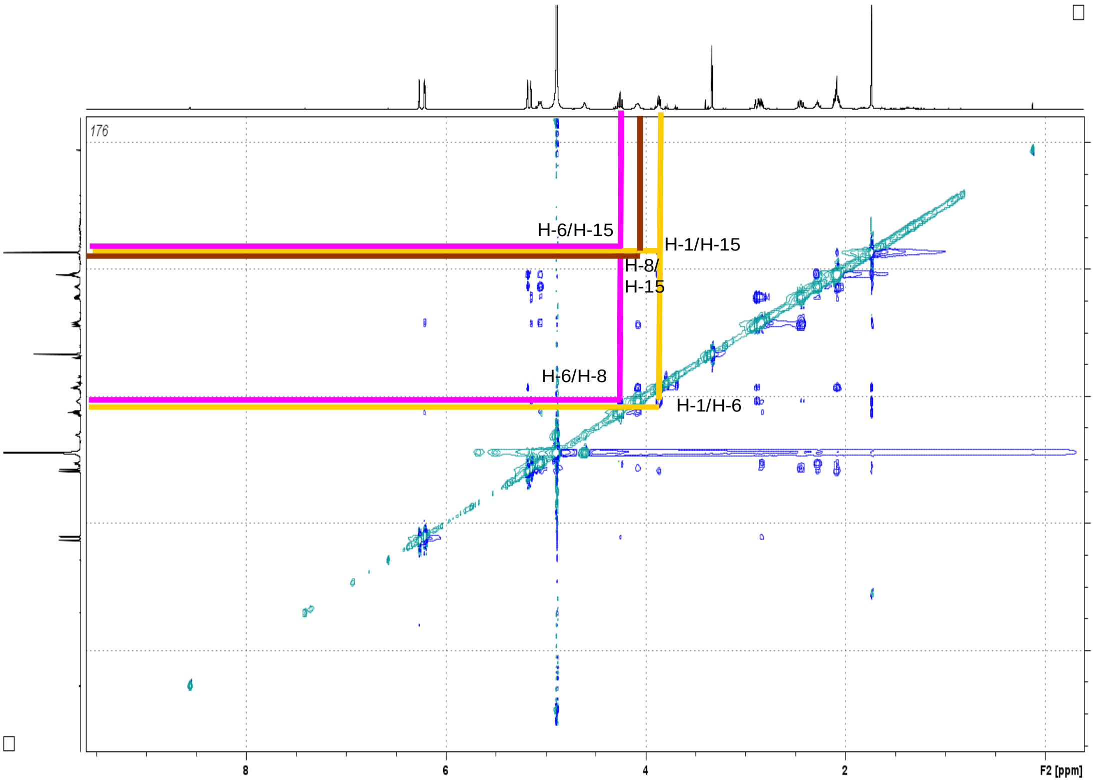

Figure S 23. NOESY-NMR spectrum of compound **3** (CD_3OD, 500MHz).

Table S6: ^{1}H and ^{13}C NMR of compound **4** ($CDCl_3$, 300MHz).

**Position**	**δ_C ($CDCl_3$)**	**C**	**δ_H ($CDCl_3$)**	**Number H**	**Multiplicity J (Hz)**
1	78.2	CH	3.52	1	*dd* (J=4.5, 11.4)
2	31.4	CH_2	1.57-1.83	2	*m*
3	33.6	CH_2	2.33	2	*ddd* (J=2.1, 5.1)
			2.10		*dd* (J=2.5, 5.3)
4	142.7	C	-	-	-
5	53.0	CH	2.17	1	*d* (J=10.8)
6	79.6	CH	4.02	1	*t* (J=10.8)
7	49.7	CH	2.53	1	*ddd* (J=3, 10.9, 11.7)
8	35.8	CH_2	2.10	2	*dd* (J=2.5, 5.3)
			1.35		*m*
9	21.6	CH_2	2.10	2	*dd* (J=2.5, 5.3)
			1.57		*m*
10	43.0	C	-	-	-
11	139.3	C	-	-	-
12	170.8	C=O	-	-	-
13	117.4	CH_2	6.08	2	*d* (J=3.3)
			5.41		*d* (J=2.9)
14	11.8	CH_3	0.80	3	*s*
15	110.7	CH_2	4.98, 4.85	2	*br s*

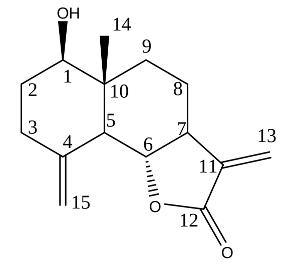

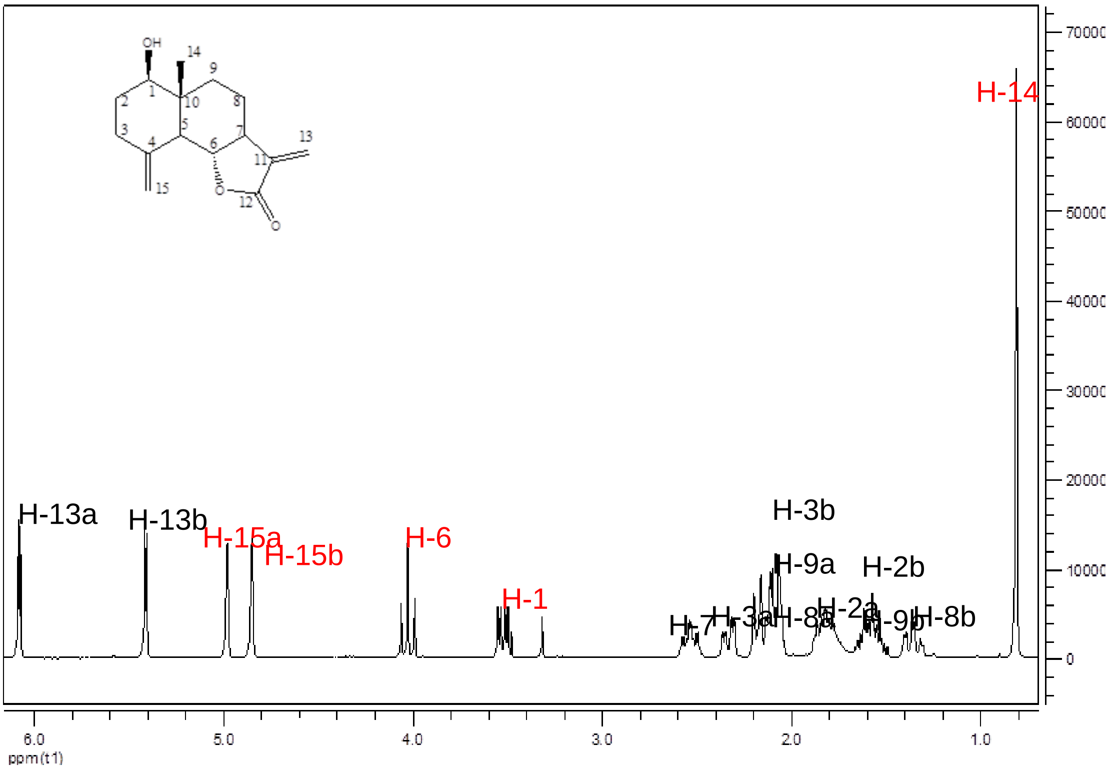

Figure S 24. ^{1}H-NMR spectrum of compound **4** ($CDCl_3$, 300MHz).

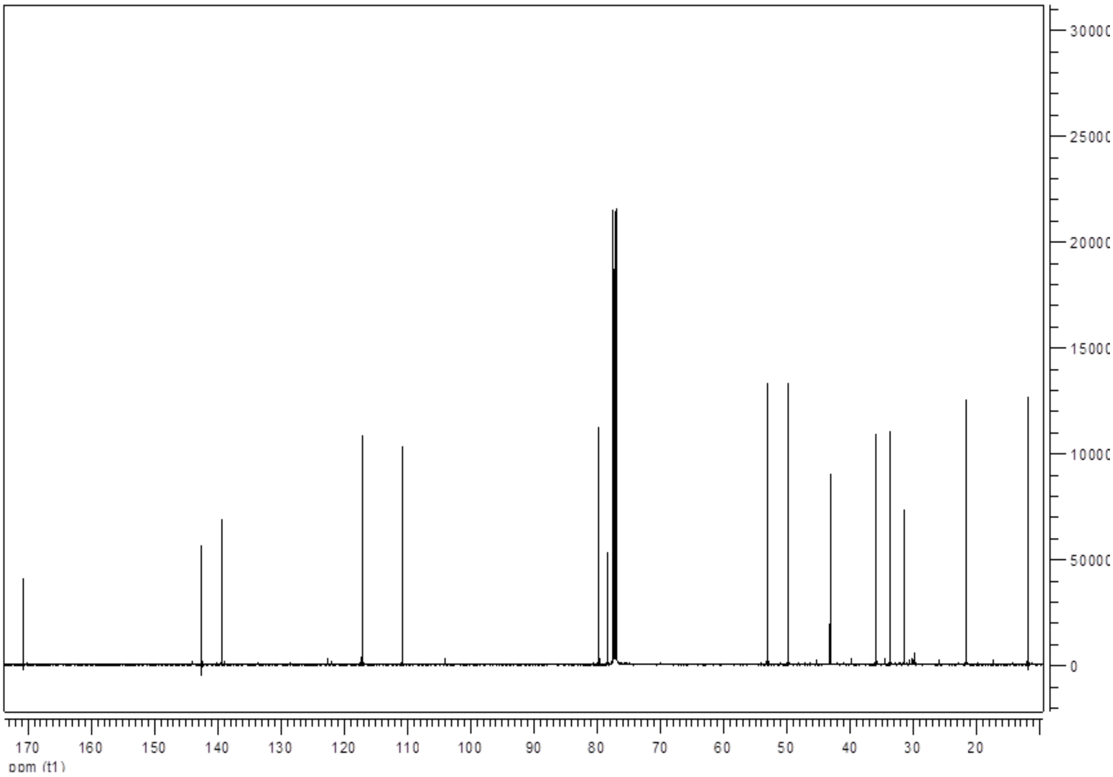

Figure S 25. ^{13}C-NMR spectrum of compound **4** ($CDCl_3$, 75MHz).

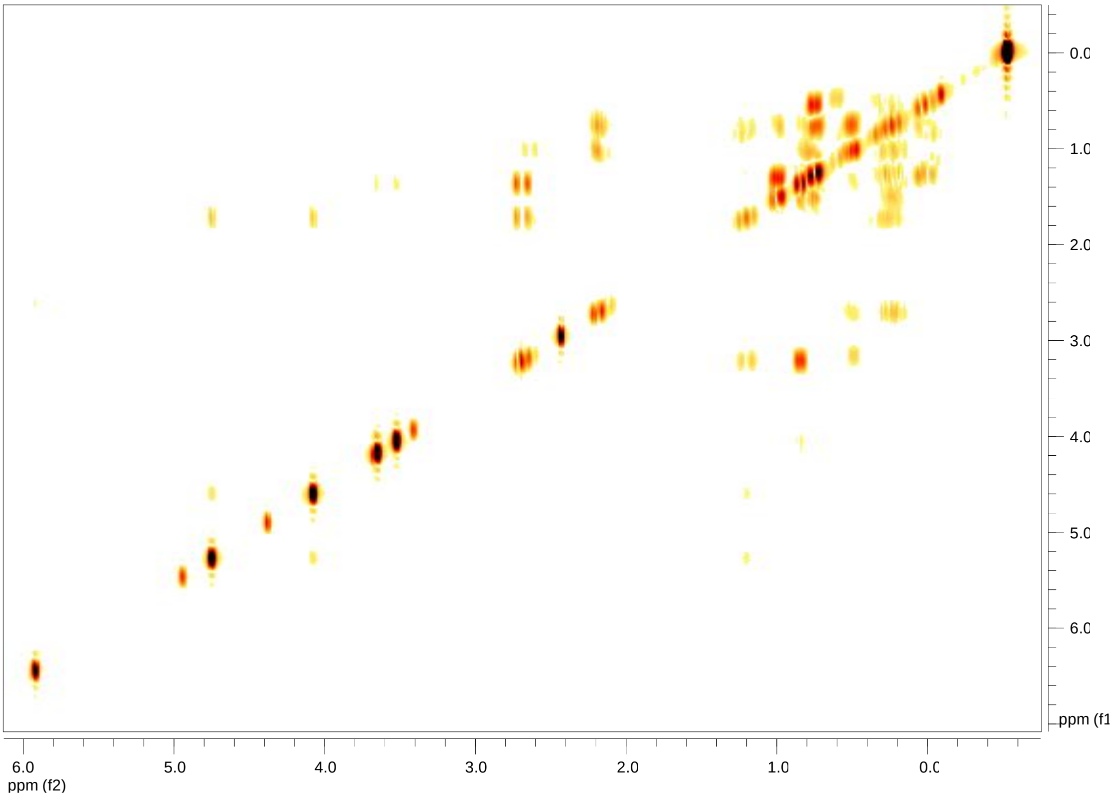

Figure S 26. COSY-NMR spectrum of compound **4** ($CDCl_3$, 300MHz).

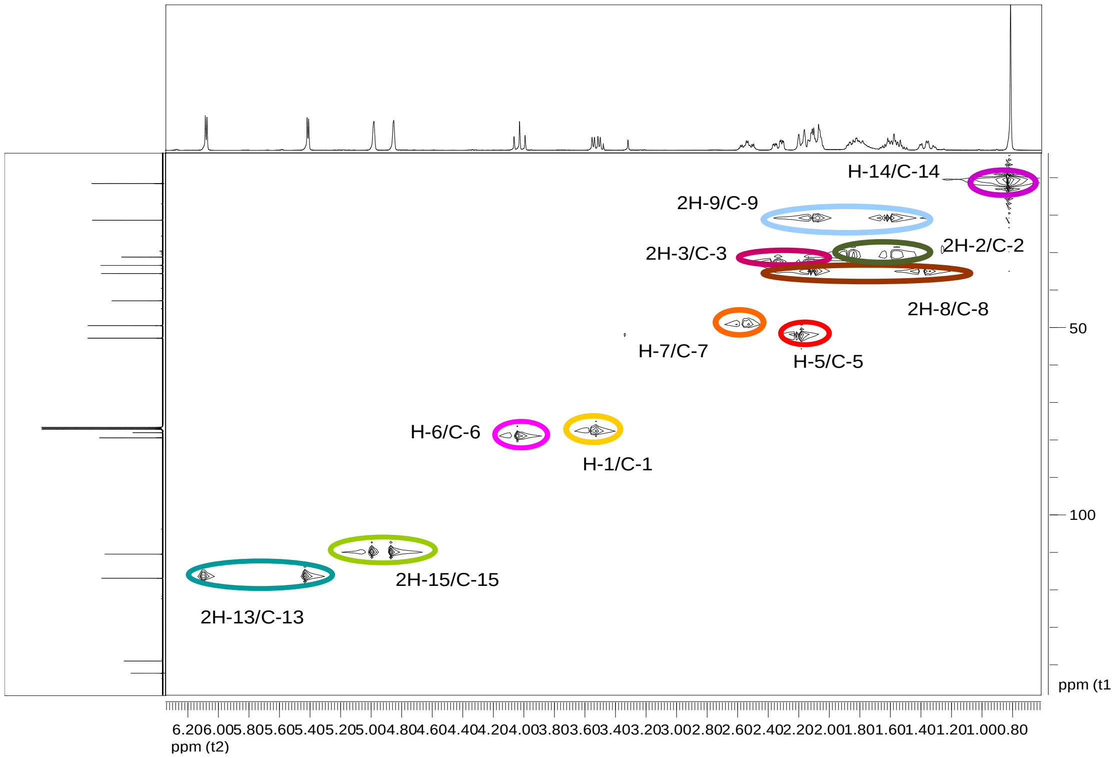

Figure S 27. HMQC-NMR spectrum of compound **4** ($CDCl_3$, 500MHz).

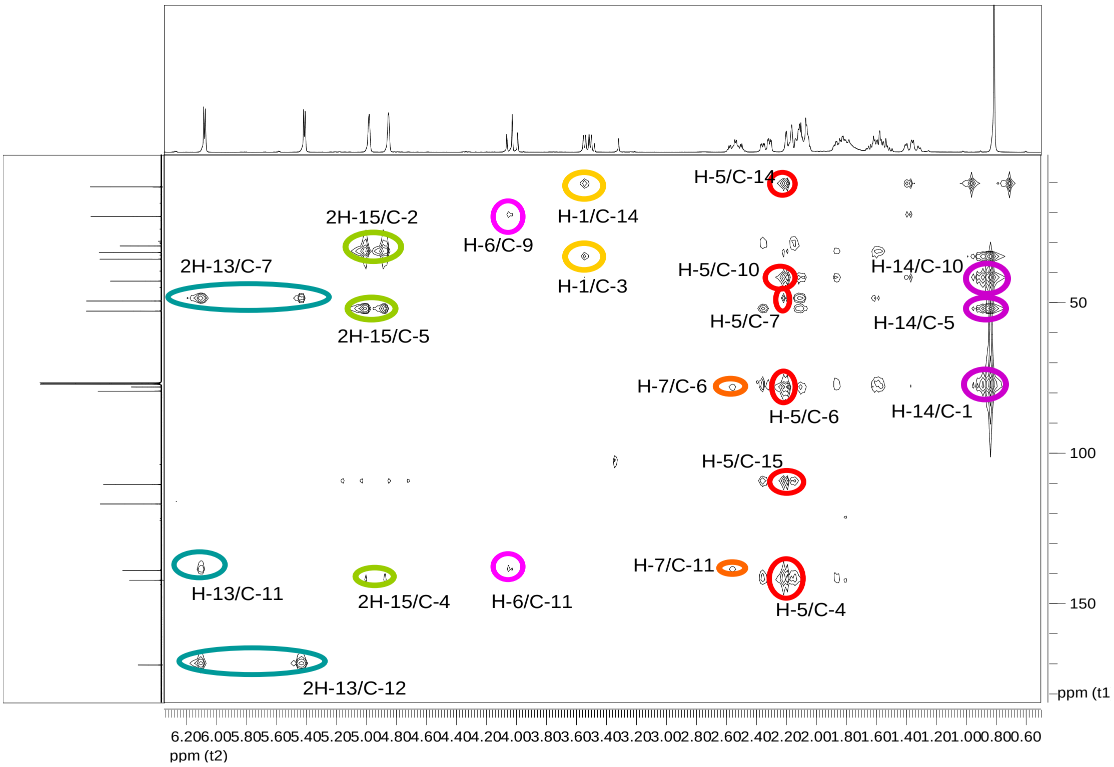

Figure S 28. HMBC-NMR spectrum of compound **4** ($CDCl_3$, 500MHz).

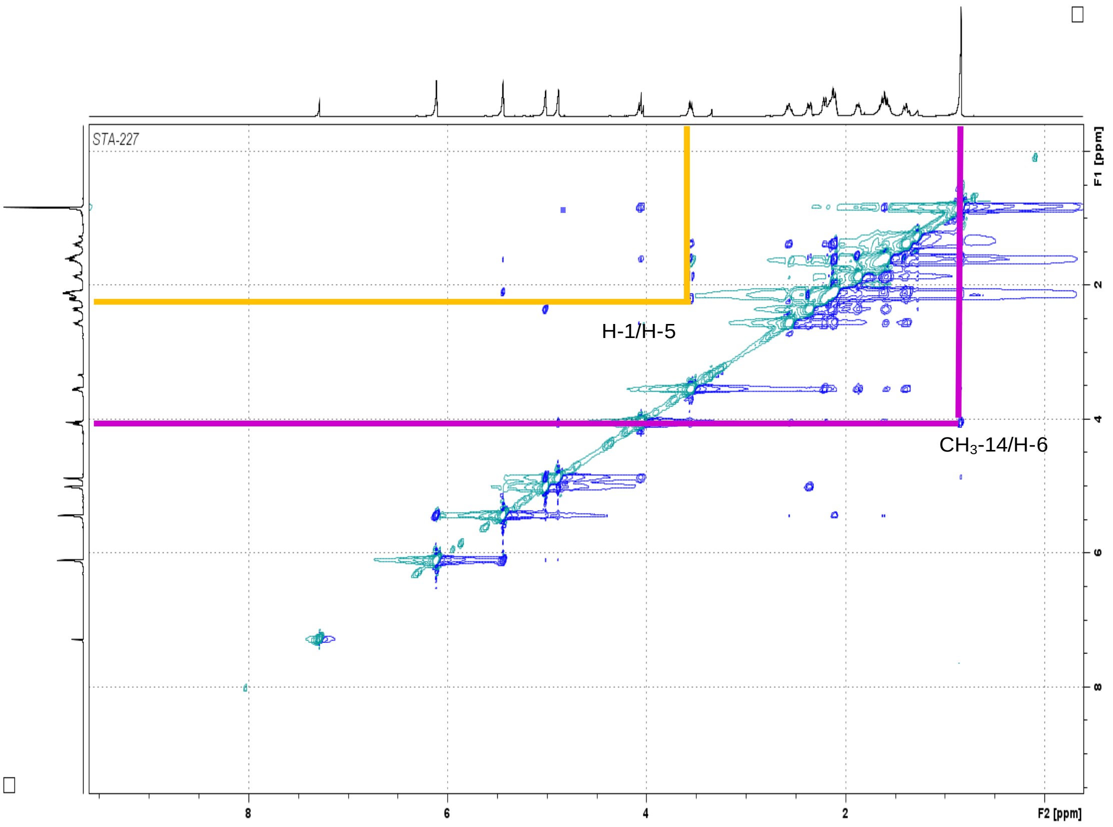

Figure S 29. NOESY-NMR spectrum of compound **4** ($CDCl_3$, 500MHz).

Table S7: ^{1}H and ^{13}C NMR of compound **5** (CD_3OD, 300MHz).

**Position**	***δ_C*** **($CDCl_3$)**	**C**	***δ_H*** **($CDCl_3$)**	**Number H**	**Multiplicity J (Hz)**
1	78.4	CH	3.46	1	*dd* (J=4.8, 11.4)
2	30.4	CH_2	-	2	-
3	33.9	CH_2	-	2	-
4	144.9	C	-	-	-
5	52.9	CH	2.34	1	*ddd* (2.1, 4.8)
6	69.4	CH	3.98	1	*dd* (J=9.9, 10.2)
7	47.4	CH	2.58	1	*dd* (J=2.5, 9.8)
8	35.6	CH_2	-	2	-
9	25.2	CH_2	-	2	-
10	42.5	C	-	-	-
11	141.4	C	-	-	-
12	165.7	C=O	-	-	-
13	125.0	CH_2	6.28, 5.72	2	*s*
14	11.1	CH_3	0.81	3	*s*
15	108.2	CH_2	5.01, 4.75	2	*d* (J=0.9)
-$COOCH_3$	51.2	-$COOCH_3$	3.77	3	*s*

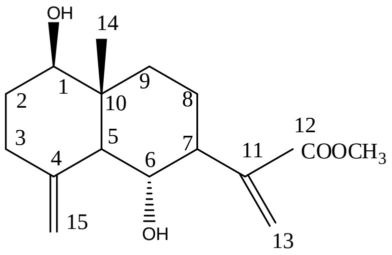

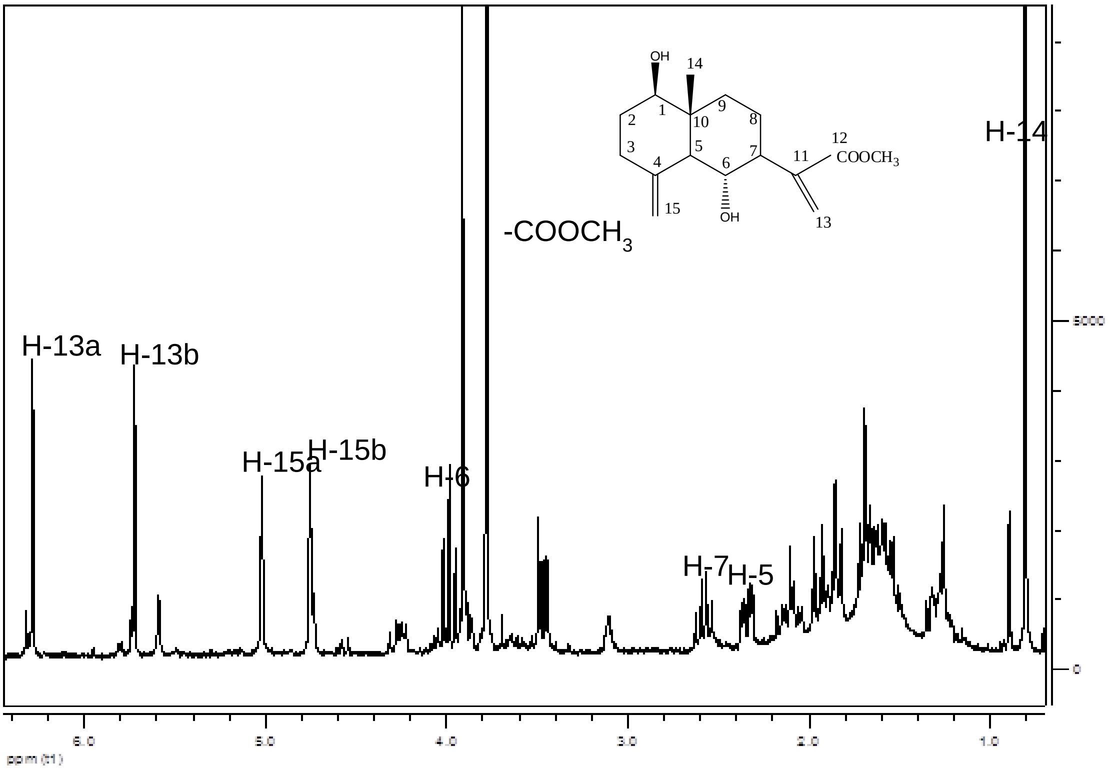

Figure S 30. ^{1}H-NMR spectrum of compound **5** (CD_3OD, 300MHz).

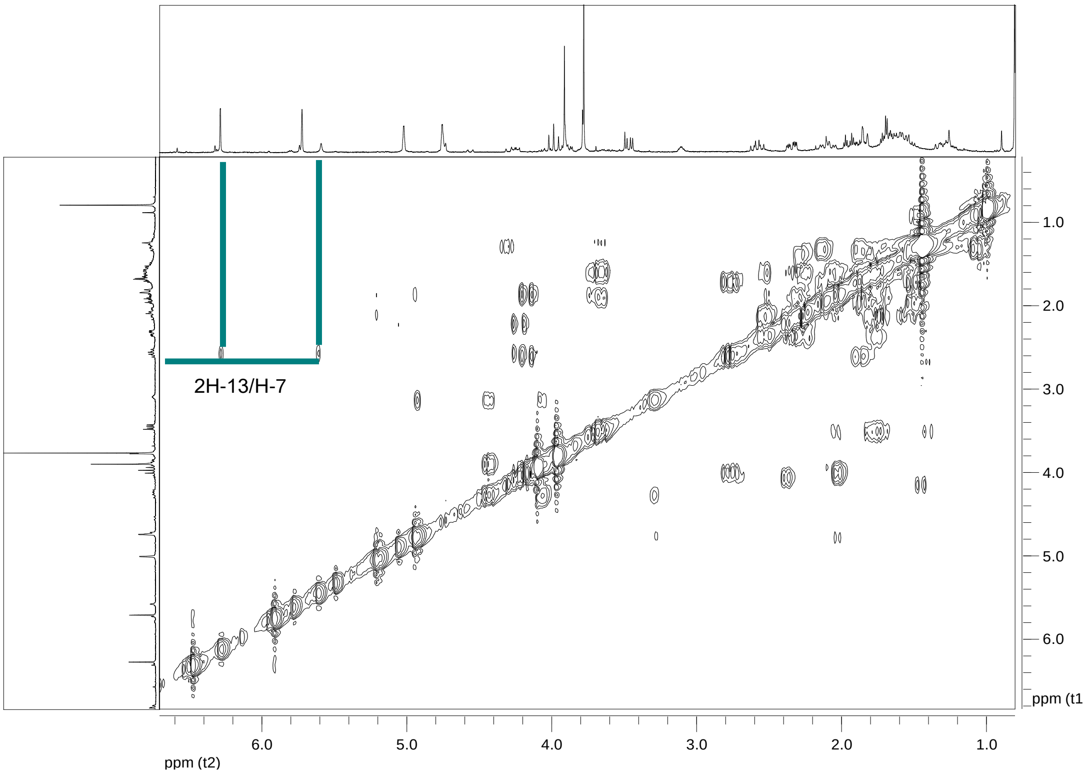

Figure S 31. COSY-NMR spectrum of compound **5** (CD_3OD, 300MHz).

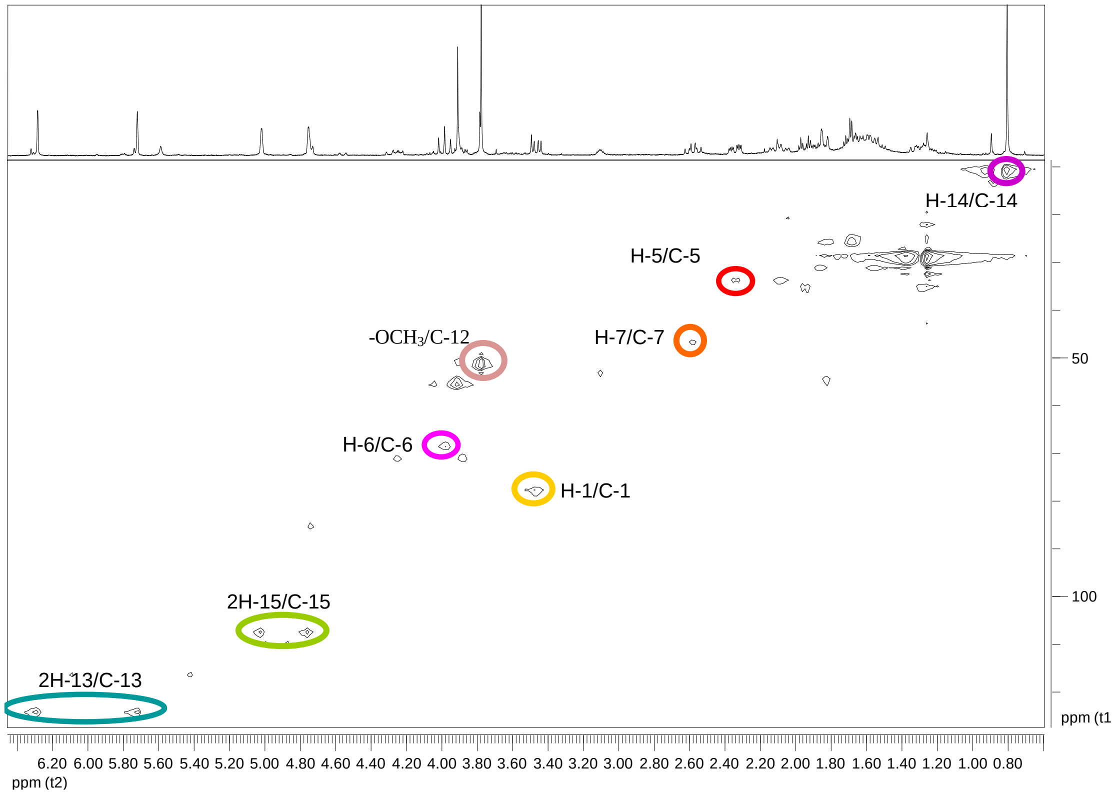

Figure S 32. HMQC-NMR spectrum of compound **5** (CD_3OD, 500MHz).

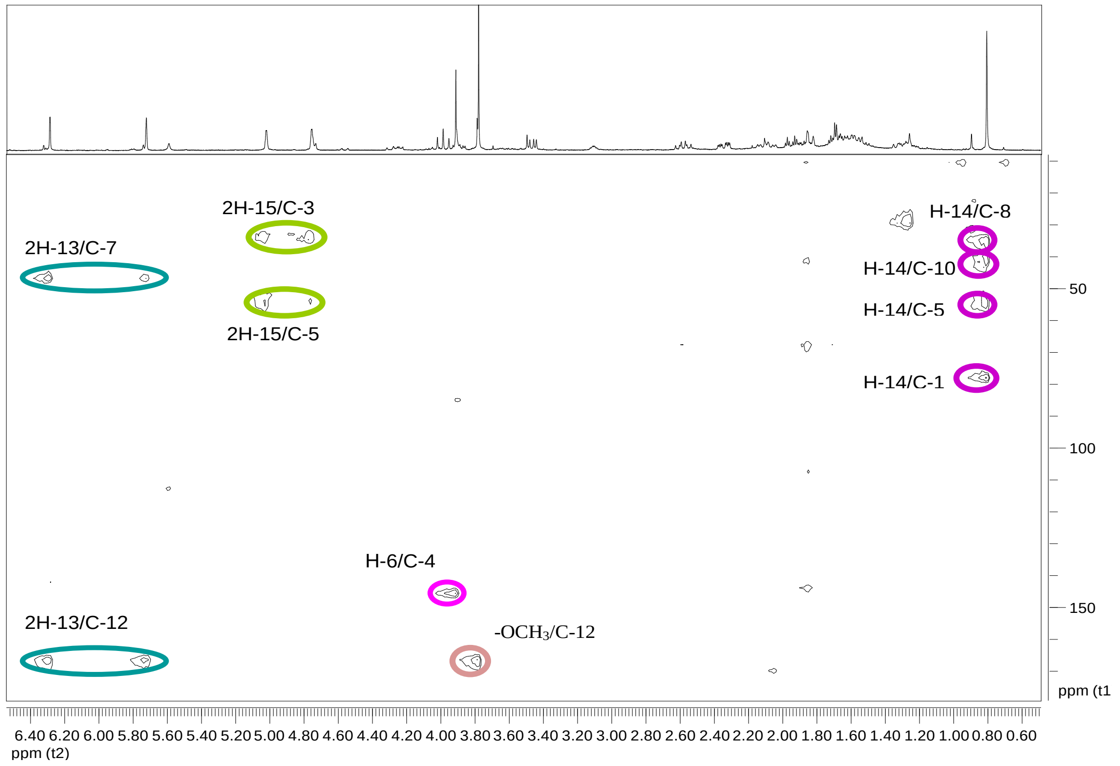

Figure S 33: HMBC-NMR spectrum of compound **5** (CD_3OD, 500MHz).

Table S8: ^{1}H and ^{13}C NMR of compound **6** ($CDCl_3$, 500MHz).

**Position**	**δ_C ($CDCl_3$)**	**C**	**δ_H ($CDCl_3$)**	**Number H**	**Multiplicity J (Hz)**
1	78.6	CH	3.56	1	*dd* (J=5.0, 11.5)
2	31.8	CH_2	1.89	2	*dd* (J=7.5)
			1.61		*m*
3	34.9	CH_2	2.36	2	*ddd* (J=1.5, 5.0, 13.5)
			2.08		*dt* (J=5.5, 13.5)
4	144.5	C	-	-	-
5	57.5	CH	1.92	1	*d* (10.0)
6	67.7	CH	4.11	1	*t* (J=9.7)
7	54.4	CH	2.58	1	*tt* (J=3.0, 10.0)
8	76.9	CH	3.97	1	*dt* (J=3.5, 11.7)
9	40.7	CH_2	2.52	2	*dd* (J=3.7, 11.7)
			1.53		*t* (J=12.0)
10	43.2	C	-	-	-
11	137.3	C	-	-	-
12	170.8	C=O	-	-	-
13	120.3	CH_2	6.16	2	*d* (J=3.0)
			5.97		*d* (J=2.5)
14	14.2	CH_3	0.81	3	*s*
15	109.1	CH_2	5.07, 4.75	2	*br s*

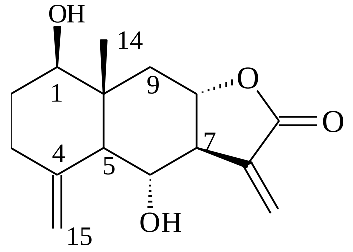

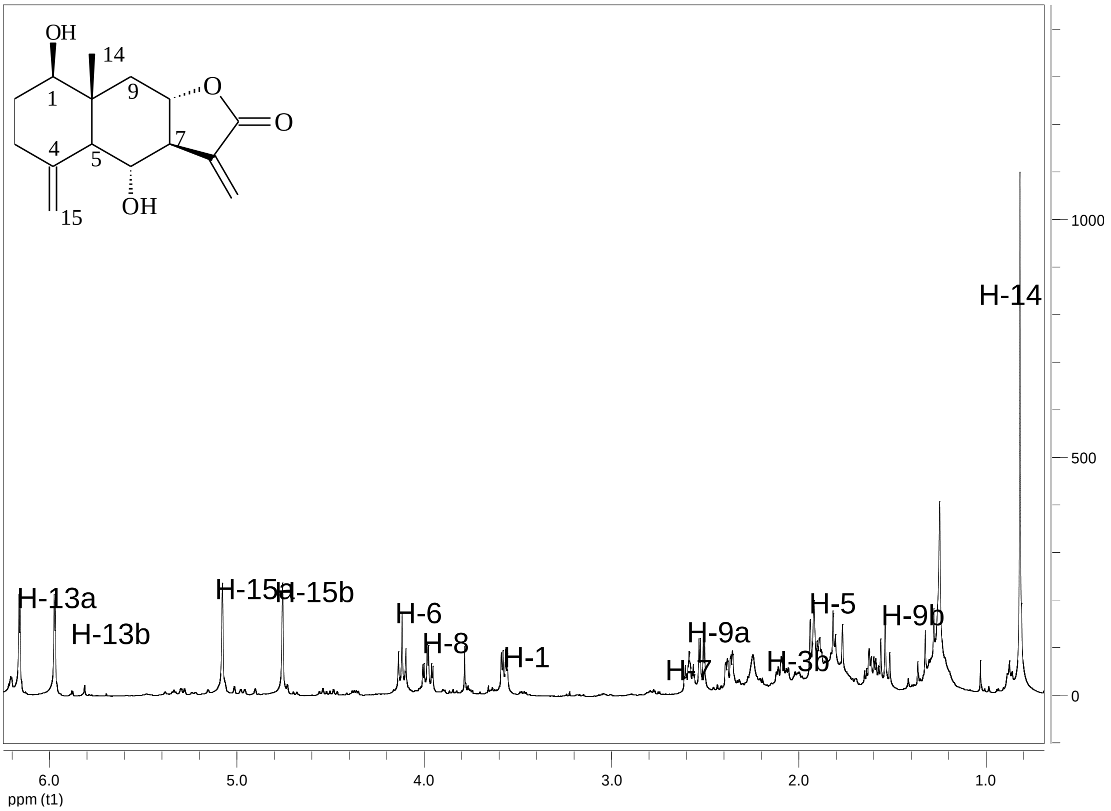

Figure S 34. ^{1}H-NMR spectrum of compound **6** ($CDCl_3$, 500MHz).

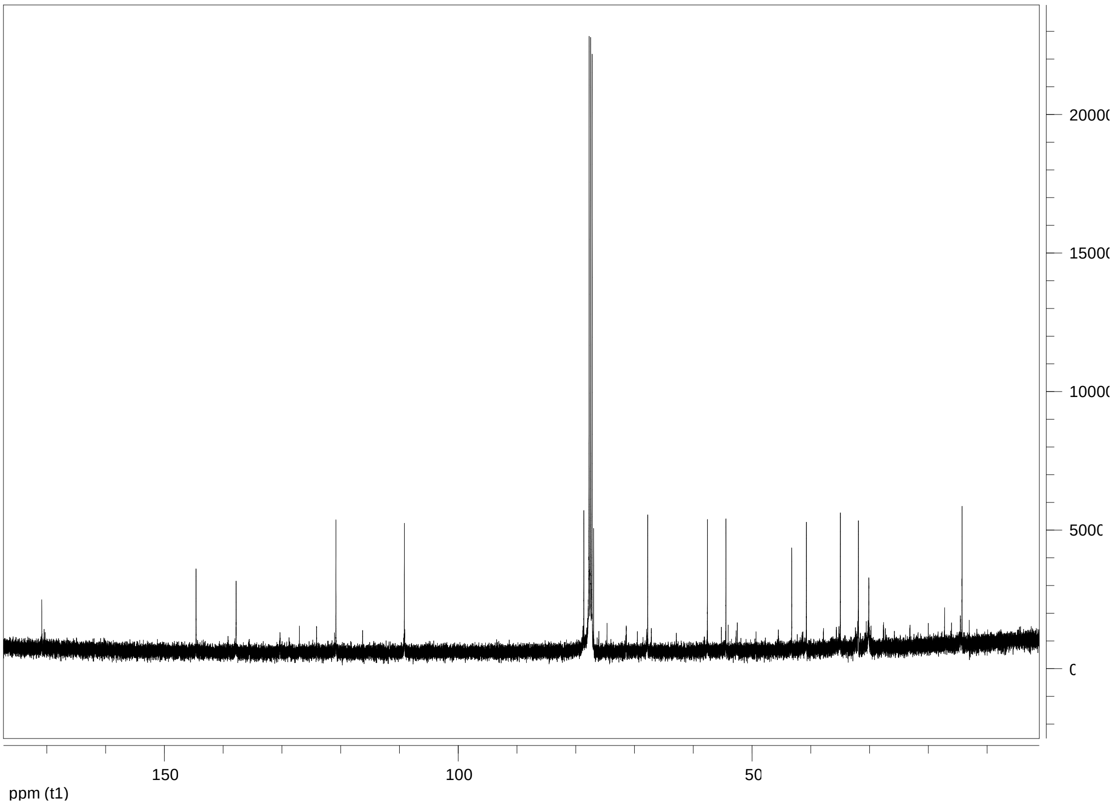

Figure S 35. ^{13}C-NMR spectrum of compound **6** ($CDCl_3$, 300MHz).

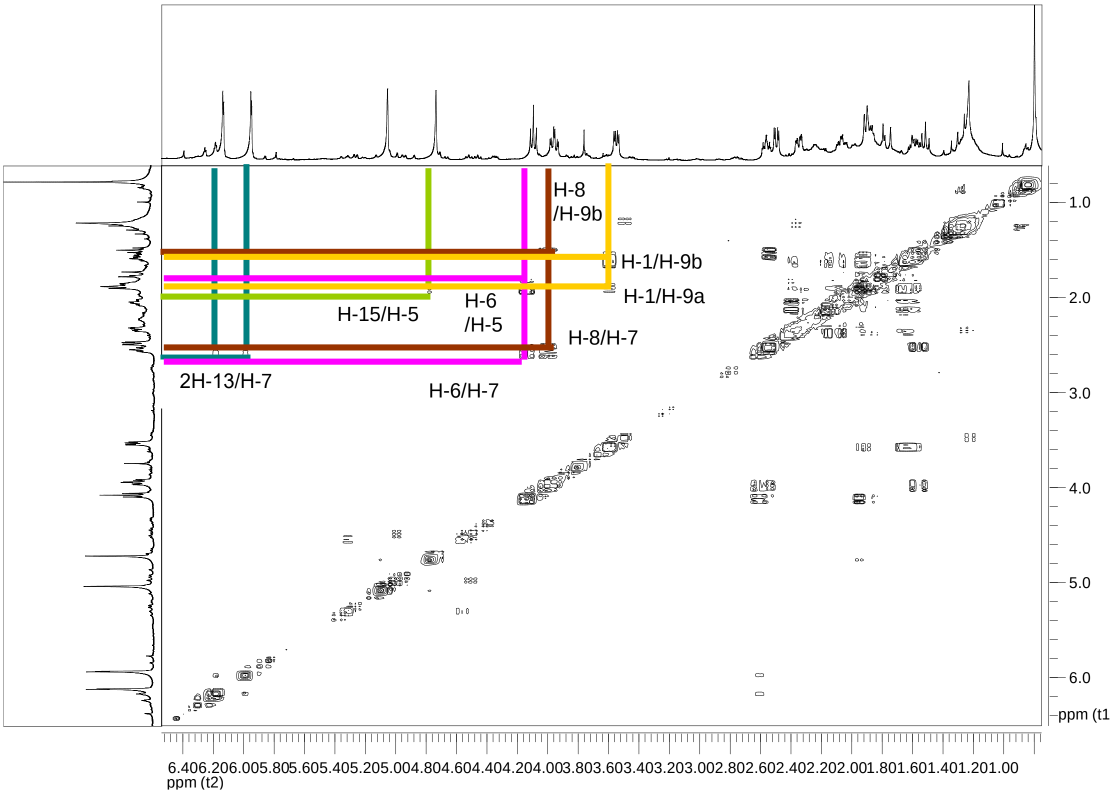

Figure S 36. COSY-NMR spectrum of compound **6** ($CDCl_3$, 300MHz).

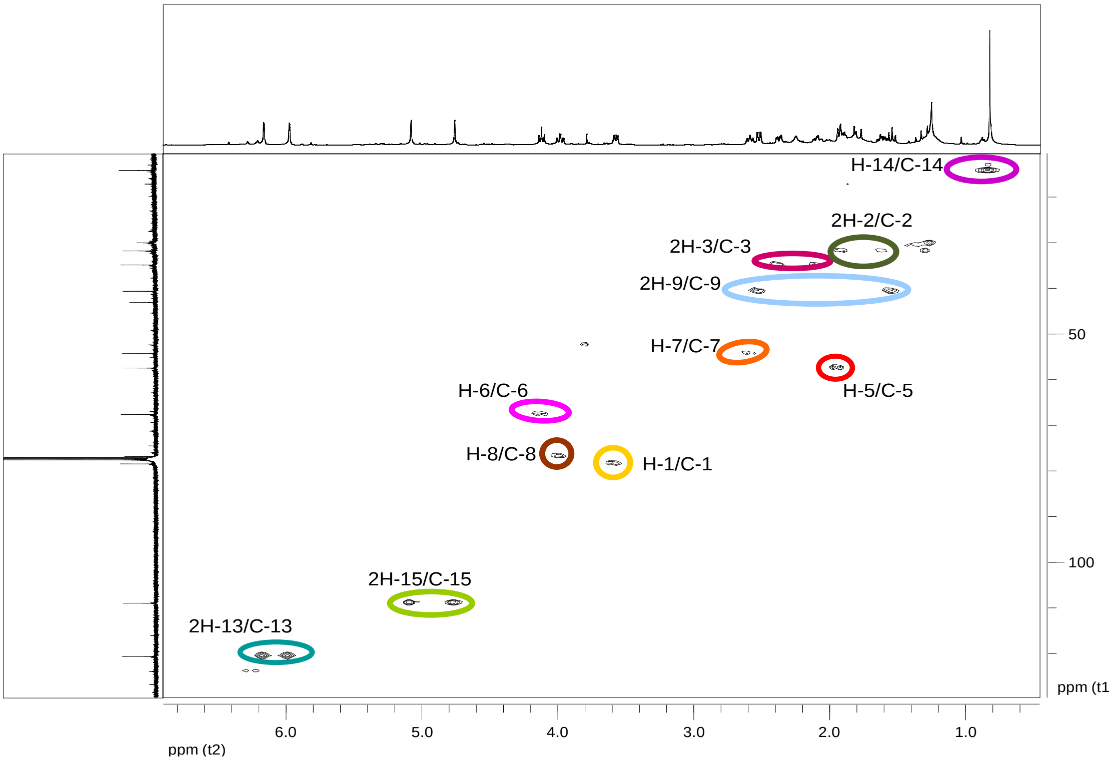

Figure S 37. HMQC-NMR spectrum of compound **6** ($CDCl_3$, 500MHz).

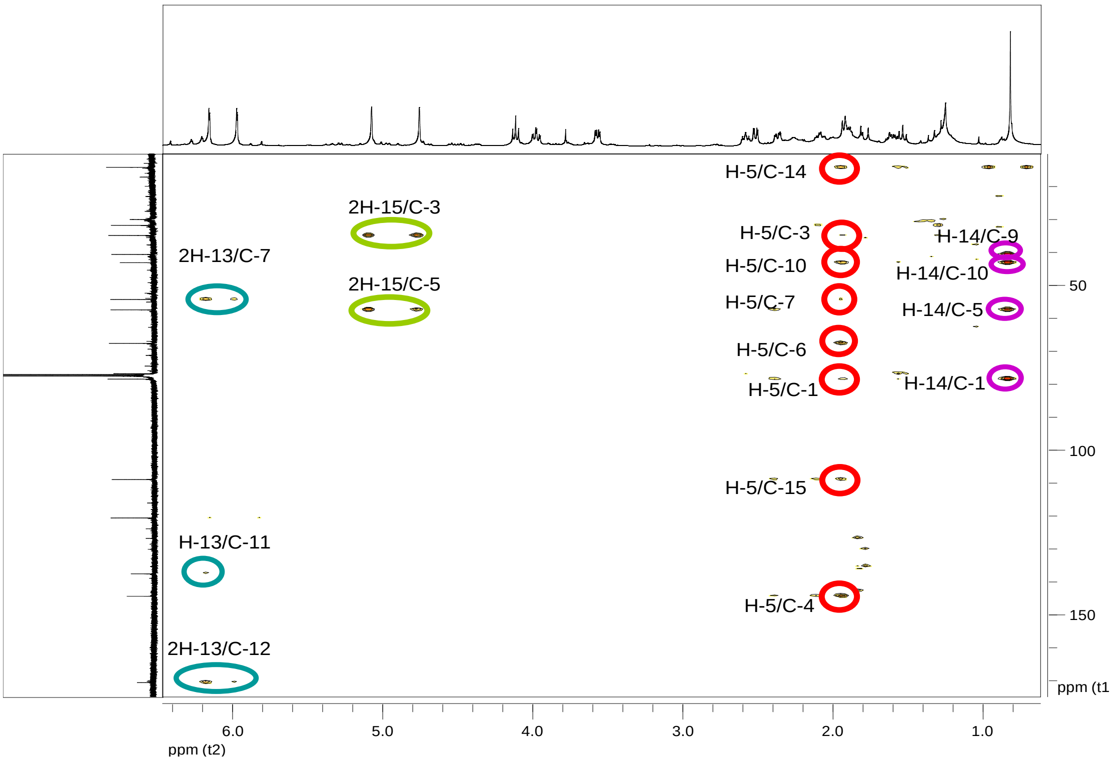

Figure S 38. HMBC-NMR spectrum of compound **6** ($CDCl_3$, 500MHz).

Table S9: ^1H and ^{13}C NMR of compound **7** (CD_3OD, 300MHz).

**Position**	δ_C **(CD_3OD)**	**C**	δ_H **(CD_3OD)**	**Number H**	**Multiplicity J (Hz)**
1	78.3	CH	3.42	1	*dd* (J=4.7, 10.4)
2	28.0	CH_2	1.76, 1.64	2	*m*
3	34.6	CH_2	-	2	-
4	74.9	C	-	-	-
5	54.4	CH	1.91	1	*d* (11.1)
6	80.1	CH	4.06	1	*t* (J=11.1)
7	50.4	CH	2.60	1	*q* (J=3.3, 11.4)
8	21.8	CH_2	2.04, 1.55	2	*m*
9	39.2	CH_2	-	2	-
10	42.2	C	-	-	-
11	139.2	C	-	-	-
12	170.9	C=O	-	-	-
13	116.9	CH_2	6.08	2	*d* (J=3.3)
			5.38		*d* (J=3.0)
14	14.1	CH_3	1.02	3	*s*
15	18.6	CH_2	1.30	2	*br s*
$-OCH_3$	48.0	$-OCH_3$	3.20		*s*

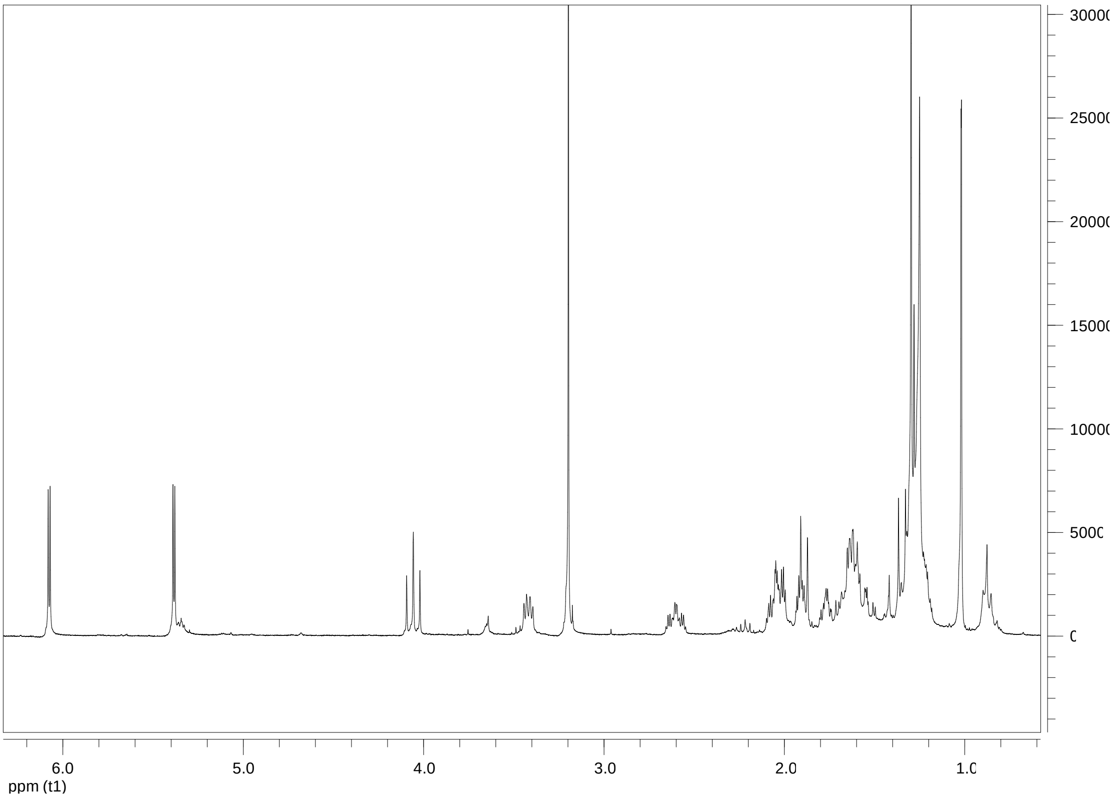

Figure S 39. ^{1}H-NMR spectrum of compound **7** (CD_3OD, 300MHz).

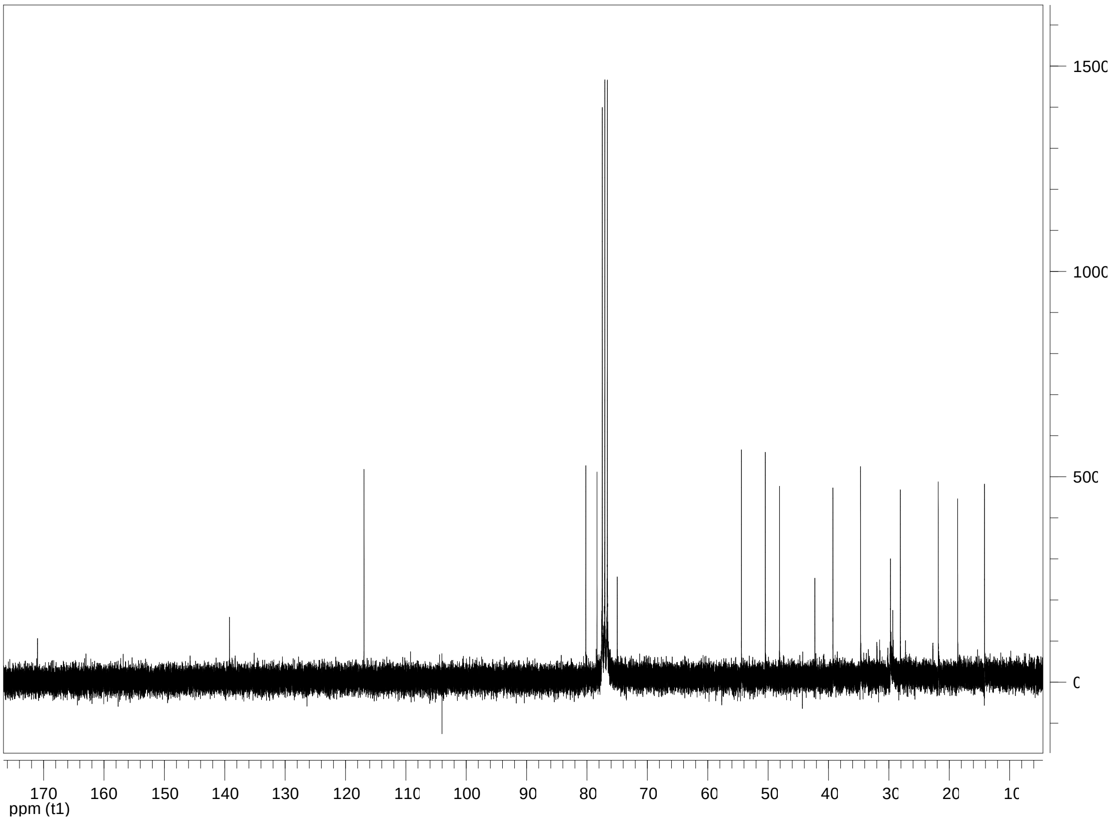

Figure S 40. ^{13}C-NMR spectrum of compound **7** (CD_3OD, 75MHz).

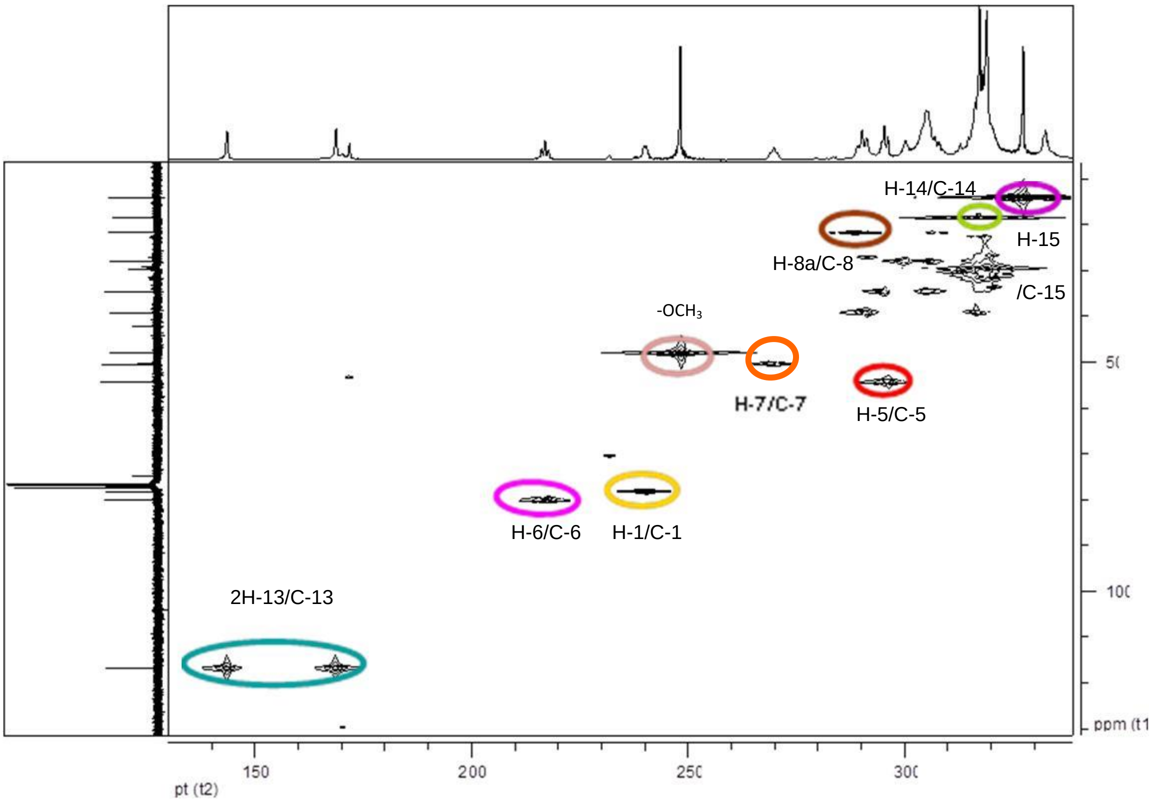

Figure S 41. HMQC-NMR spectrum of compound **7** (CD_3OD, 500MHz).

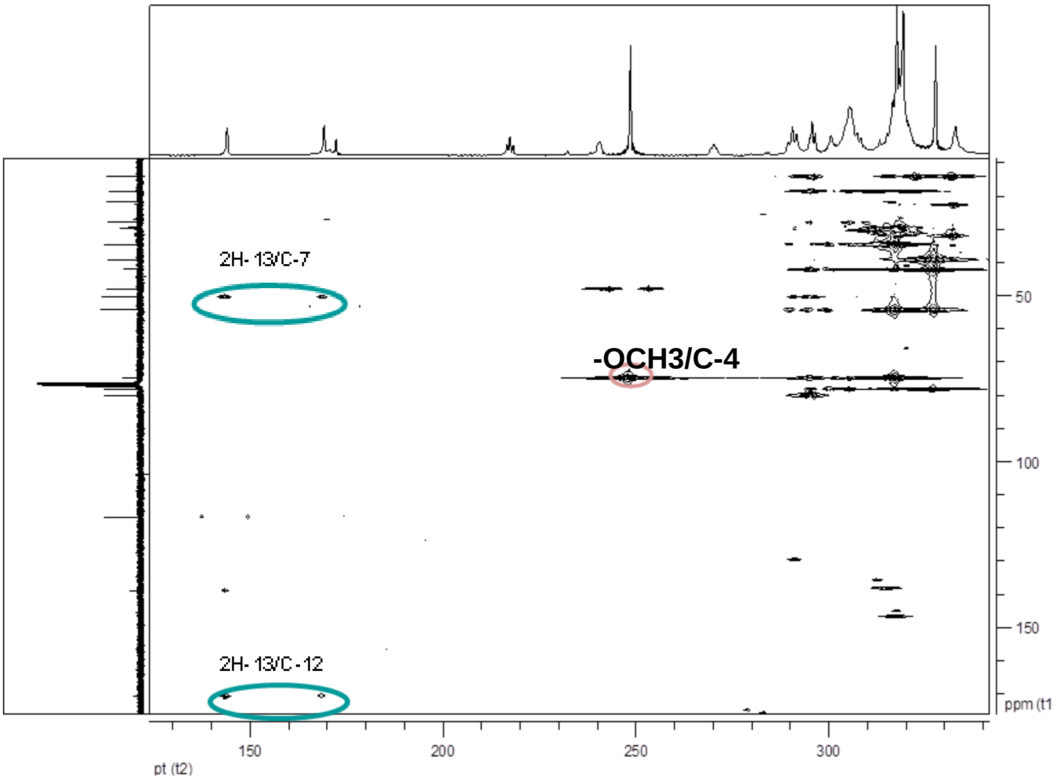

Figure S 42. HMBC-NMR spectrum of compound **7** (CD_3OD, 500MHz).

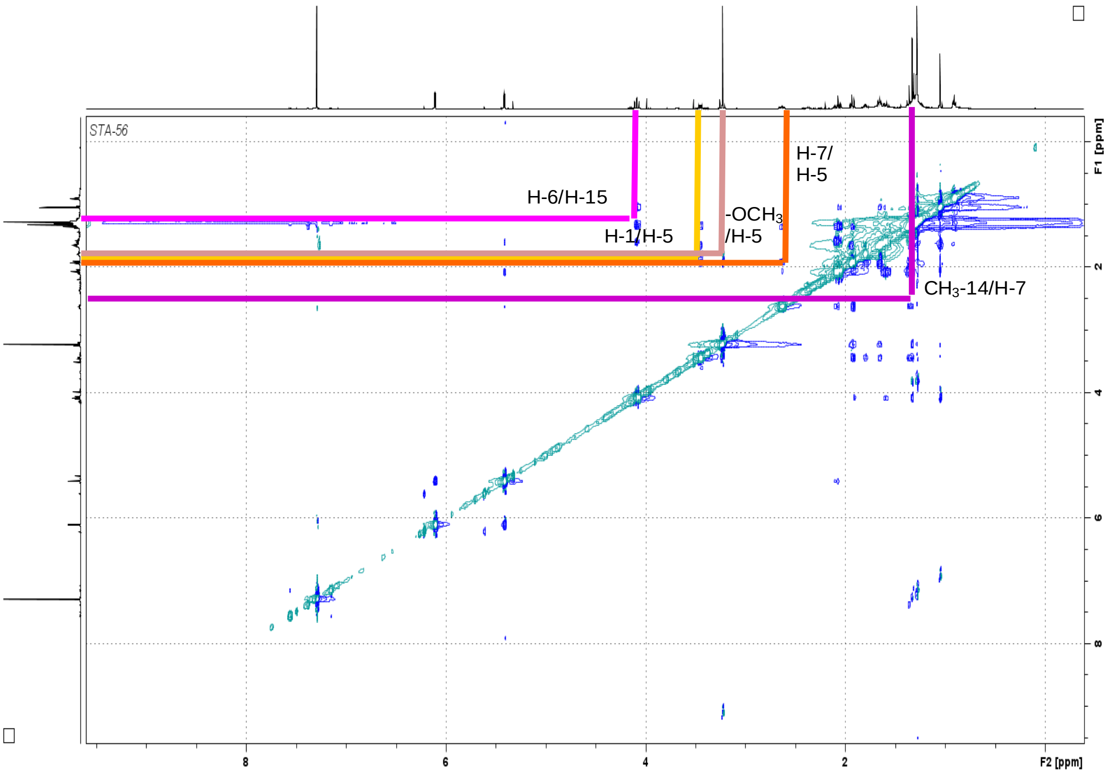

Figure S 43. NOESY-NMR spectrum of compound **7** (CD_3OD, 500MHz).

Table S10: ^{1}H and ^{13}C NMR of compound **8** ($CDCl_3$, 500MHz).

**Position**	***δ$_C$*** **($CDCl_3$)**	**C**	***δ$_H$*** **($CDCl_3$)**	**Number H**	**Multiplicity J (Hz)**
1	77.3	CH	3.48	1	*m*
2	28.0	CH_2	1.69	2	*m*
3	41.3	CH_2	1.84, 1.64	2	*m*
4	75.4	C	-	-	-
5	57.7	CH	1.55	1	*d* (10.0)
6	70.3	CH	4.27	1	*t* (J=9.8)
7	56.4	CH	2.60	1	*m*
8	75.8	CH	3.93	1	*td* (J=3.5, 11.8)
9	43.5	CH_2	2.46	2	*dd* (J=3.5, 11.5)
			1.53		*m*
10	40.0	C	-	-	-
11	137.8	C	-	-	-
12	169.2	C=O	-	-	-
13	119.8	CH_2	6.15	2	*d* (J=2.5)
			5.98		*d* (J=2.5)
14	16.3	CH_3	0.98	3	*s*
15	24.1	CH_3	1.44	3	*s*

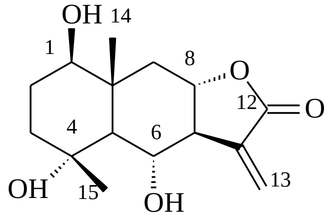

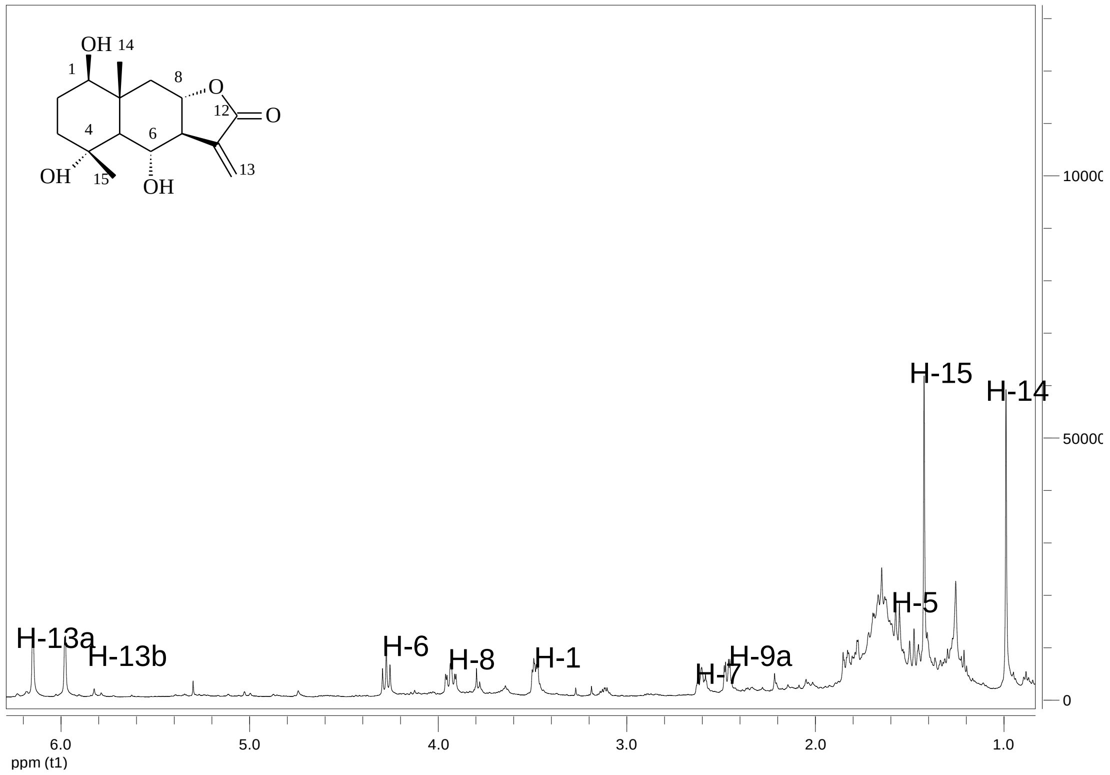

Figure S 44. ^{1}H-NMR spectrum of compound **8** ($CDCl_3$, 500MHz).

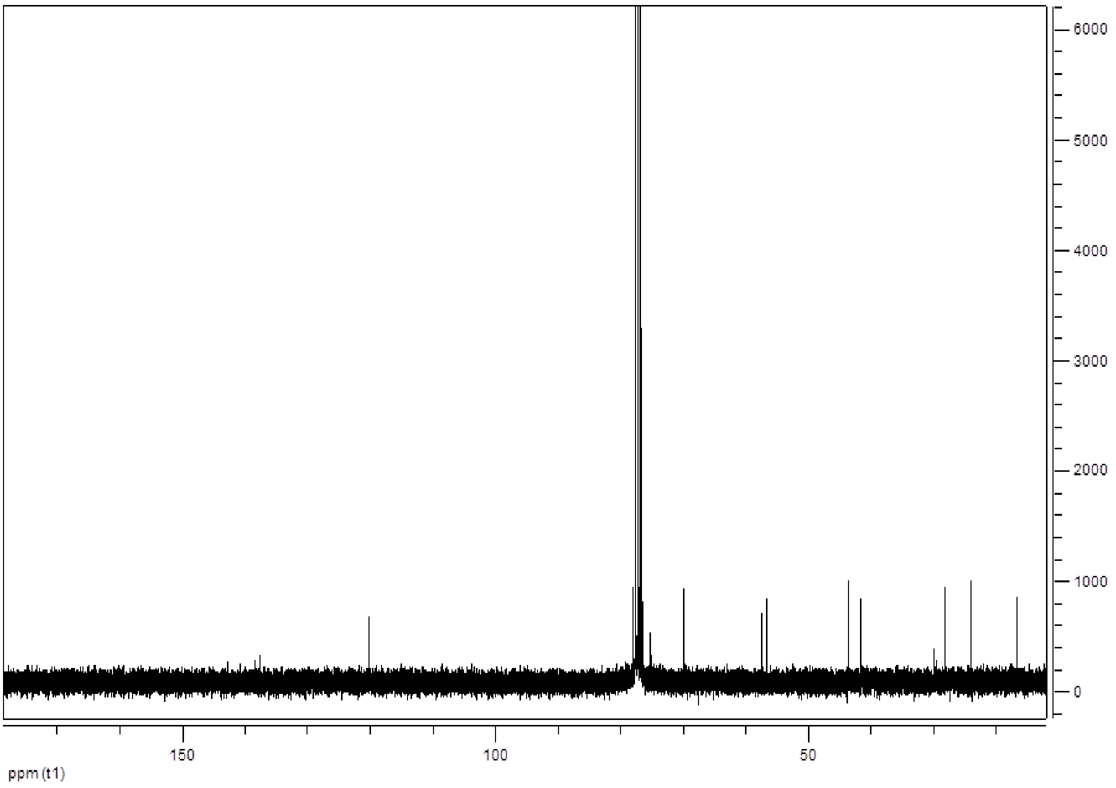

Figure S 45. ^{13}C-NMR spectrum of compound **8** ($CDCl_3$, 75MHz).

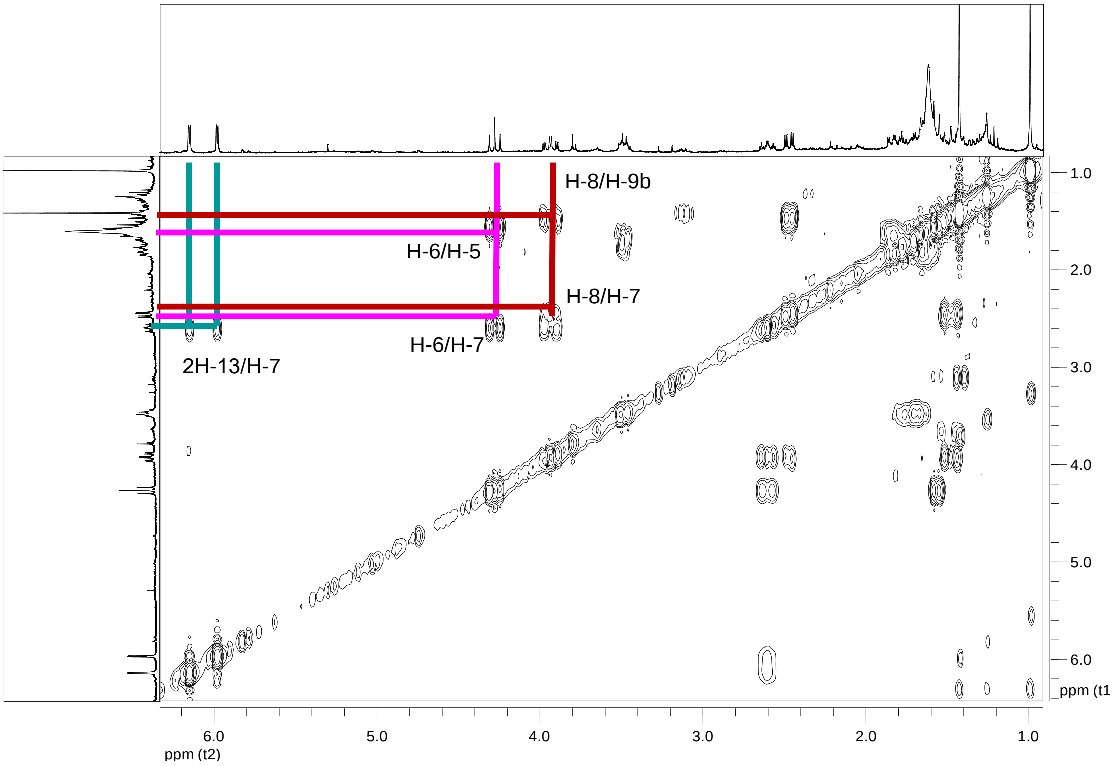

Figure S 46. COSY-NMR spectrum of compound **8** ($CDCl_3$, 300MHz).

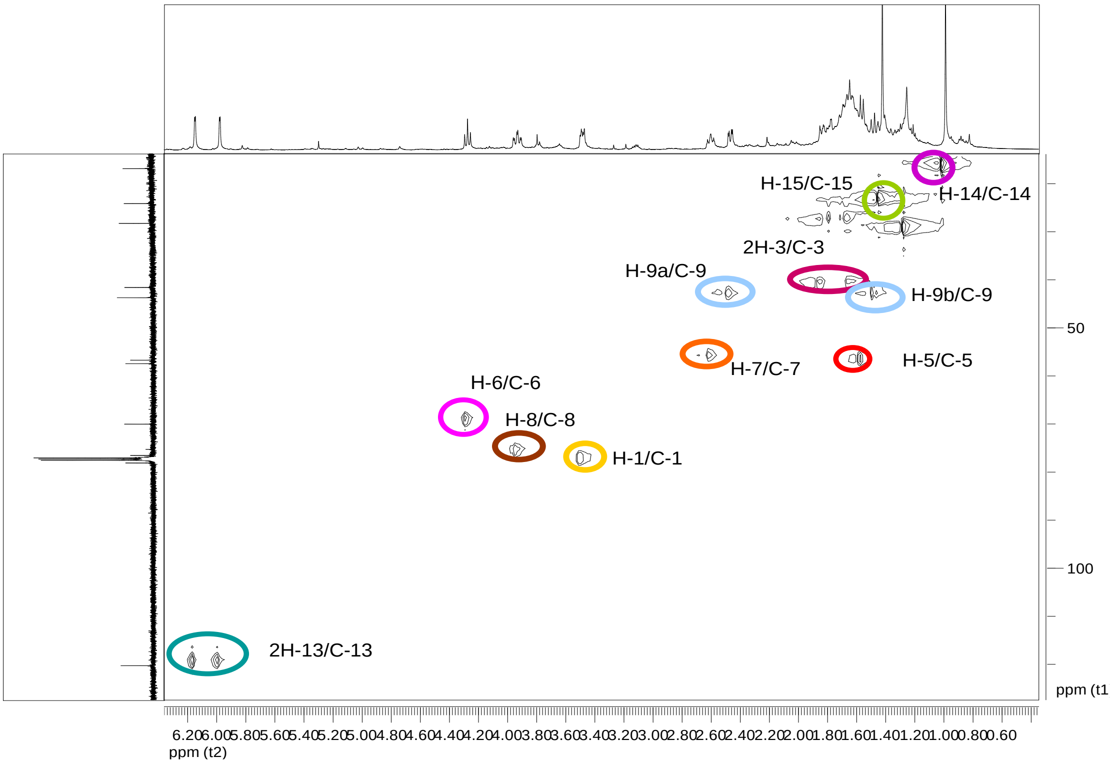

Figure S 47. HMQC-NMR spectrum of compound **8** ($CDCl_3$, 500MHz).

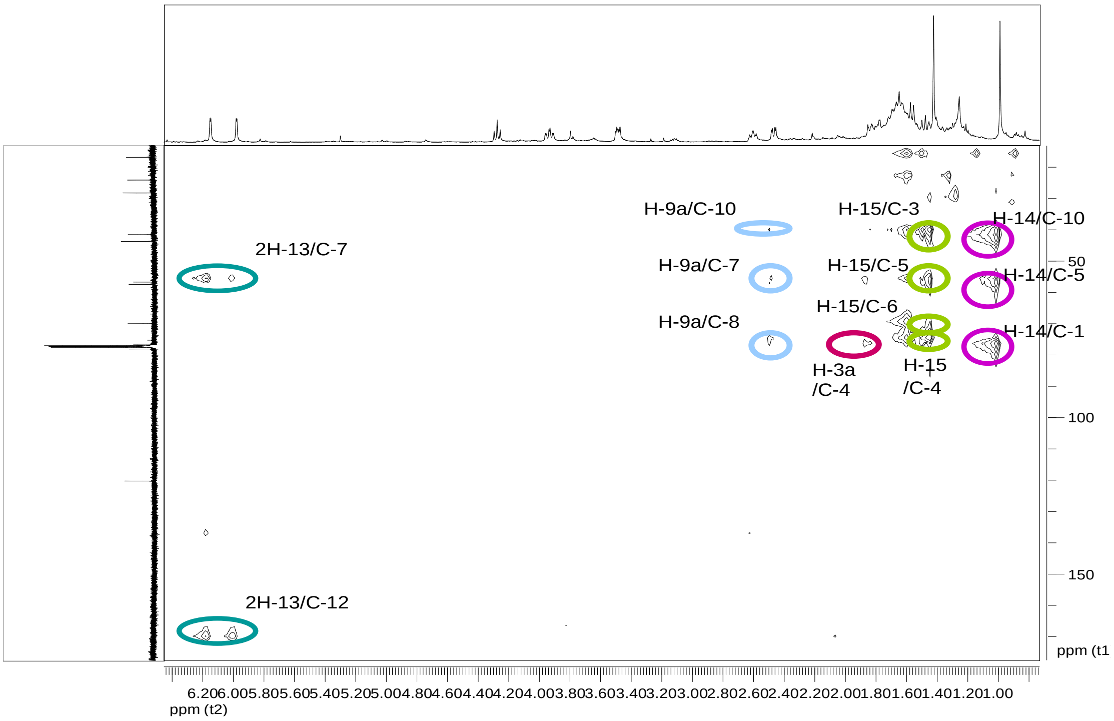

Figure S 48. HMBC-NMR spectrum of compound **8** ($CDCl_3$, 500MHz).

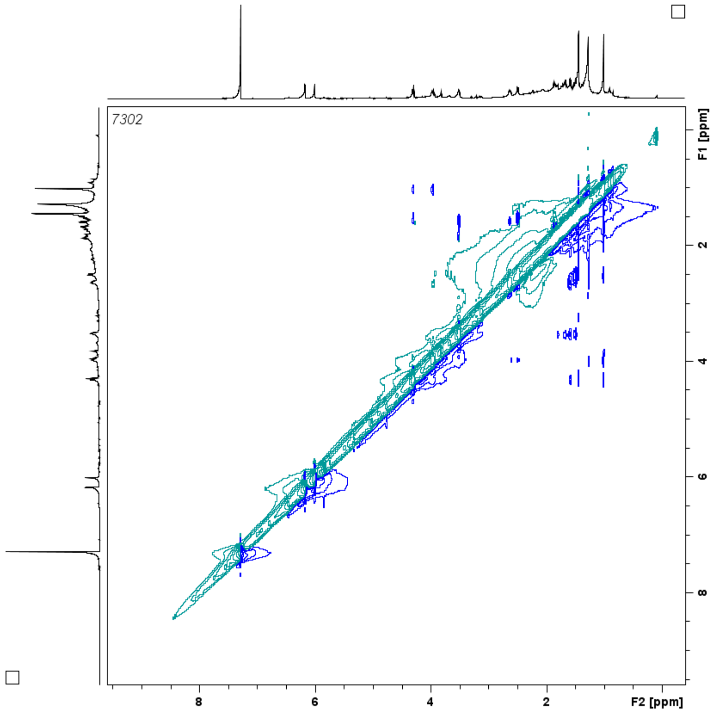

Figure S 49. NOESY-NMR spectrum of compound **8** ($CDCl_3$, 300MHz).

Table S11: ^{1}H and ^{13}C NMR of compound **9** (CD_3OD, 300MHz).

Position	δ_C (CD_3OD)	C	δ_H (CD_3OD)	Number H	Multiplicity J (Hz)
1	82.4	CH	3.27	1	*d* (J=3.9)
2	32.3	CH_2	1.94	2	*br d* (J=3.3)
			1.59		*br d* (J=3.3)
3	40.9	CH_2	1.67	2	*br d* (J=3.3)
			1.63		*br d* (J=4.5)
4	79.5	C	-	-	-
5	56.1	CH	1.53	1	*brd* (J=6.3)
6	71.6	CH	4.26	1	*t* (J=10.8)
7	52.2	CH	2.60	1	*dt* (J=3.3, 11.4, 11.2)
8	27.8	CH_2	2.06	2	*dd* (J=3.4, 6.4)
			1.56		*br d* (J=2.7)
9	39.2	CH_2	1.28	2	*dd* (J=3.4, 8.8)
			2.00		*dd* (J=3.0, 9.9)
10	43.7	C	-	-	-
11	141.3	C	-	-	-
12	172.8	C=O	-	-	-
13	117.1	CH_2	5.99	2	*d* (J=3.3)
			5.48		*d* (J=3.0)
14	13.7	CH_3	1.12	3	*br s*
15	22.6	CH_3	1.38	3	*br s*

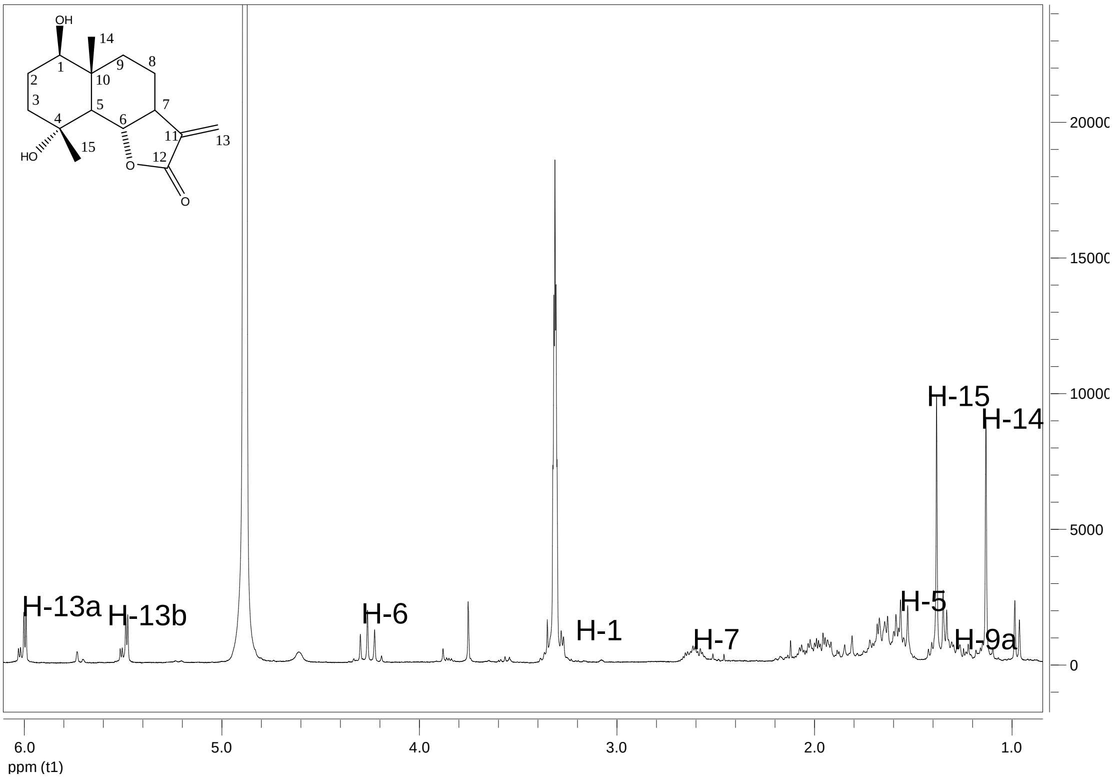

Figure S 50. ^{1}H-NMR spectrum of compound **9** (CD_3OD, 300MHz).

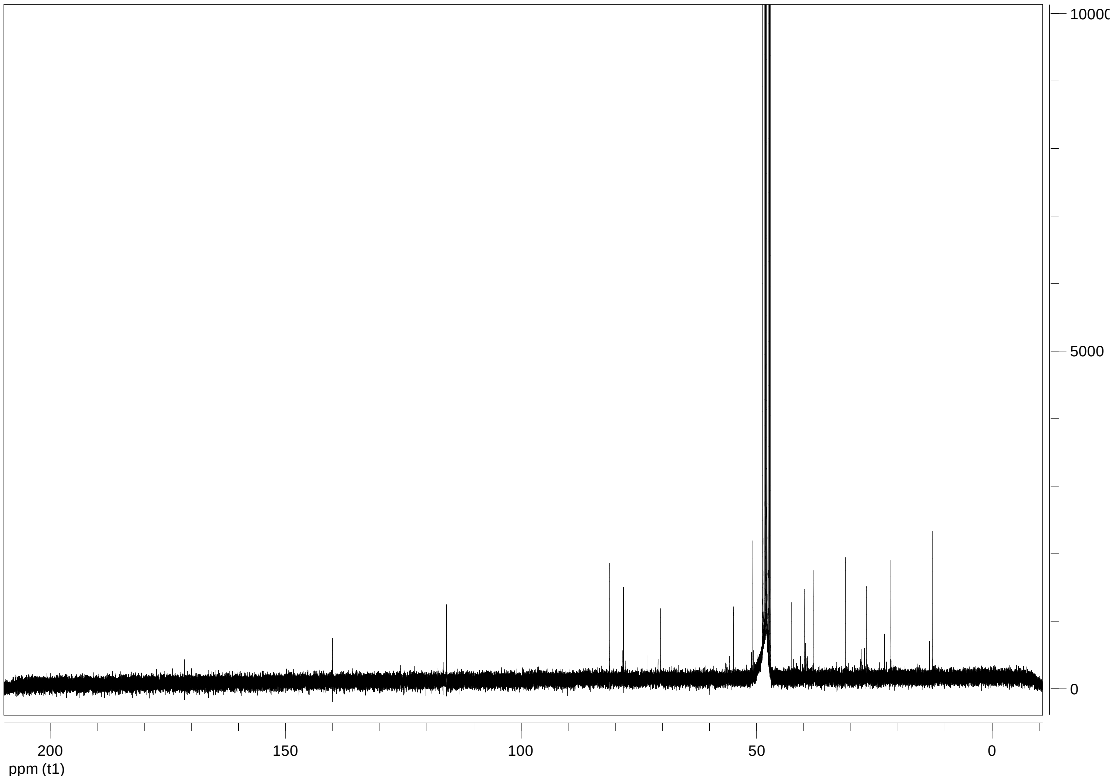

Figure S 51. ^{13}C-NMR spectrum of compound **9** (CD_3OD, 75MHz).

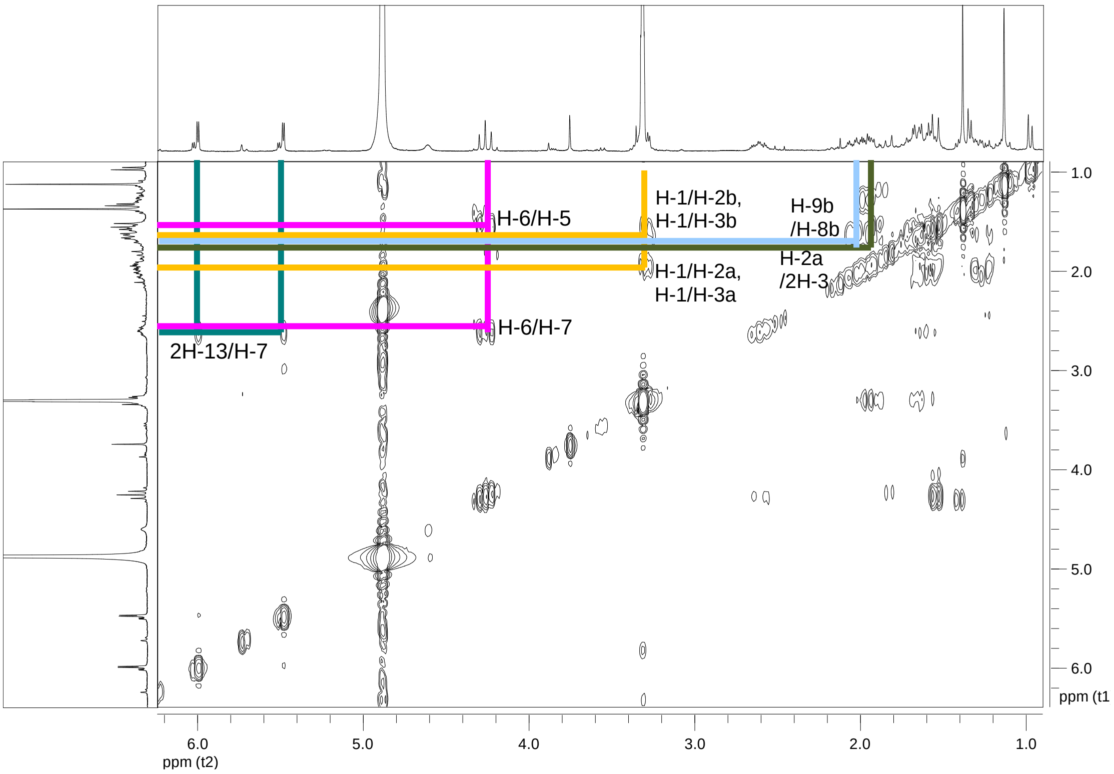

Figure S 52. COSY-NMR spectrum of compound **9** (CD_3OD, 500MHz).

Table S12: ^{1}H and ^{13}C NMR of compound **10** (CD_3OD, 300MHz).

Position	δ_C (CD_3OD)	C	δ_H (CD_3OD)	Number H	Multiplicity J (Hz)
1	79.6	CH	3.26	1	*br d* (J=8.0)
2	28.8	CH_2	1.71	2	*br s*
			1.60		*m*
3	41.1	CH_2	1.73	2	*br s*
			1.60		*m*
4	74.3	C	-	-	-
5	57.0	CH	1.40	1	*d* (J=11.0)
6	72.3	CH	4.29	1	*dd* (J=10.5, 10.2)
7	52.2	CH	2.52	1	*ddd* (J=4.5, 11.2)
8	28.3	CH_2	1.66	2	*m*
9	40.8	CH_2	1.18	2	*dd* (J=4.2, 12.2)
			1.87		*d* (J=12.5)
10	41.9	C	-	-	-
11	143.6	C	-	-	-
12	169.0	C=O	-	-	-
13	126.9	CH_2	6.25, 5.73	2	*br s*
14	14.5	CH_3	0.95	3	*br s*
15	24.0	CH_3	1.33	3	*br s*
-COOCH3	52.3	-COOCH3	3.75	3	*br s*

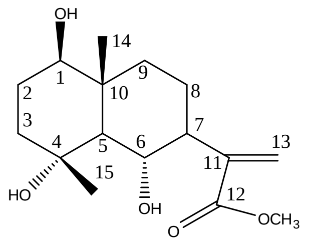

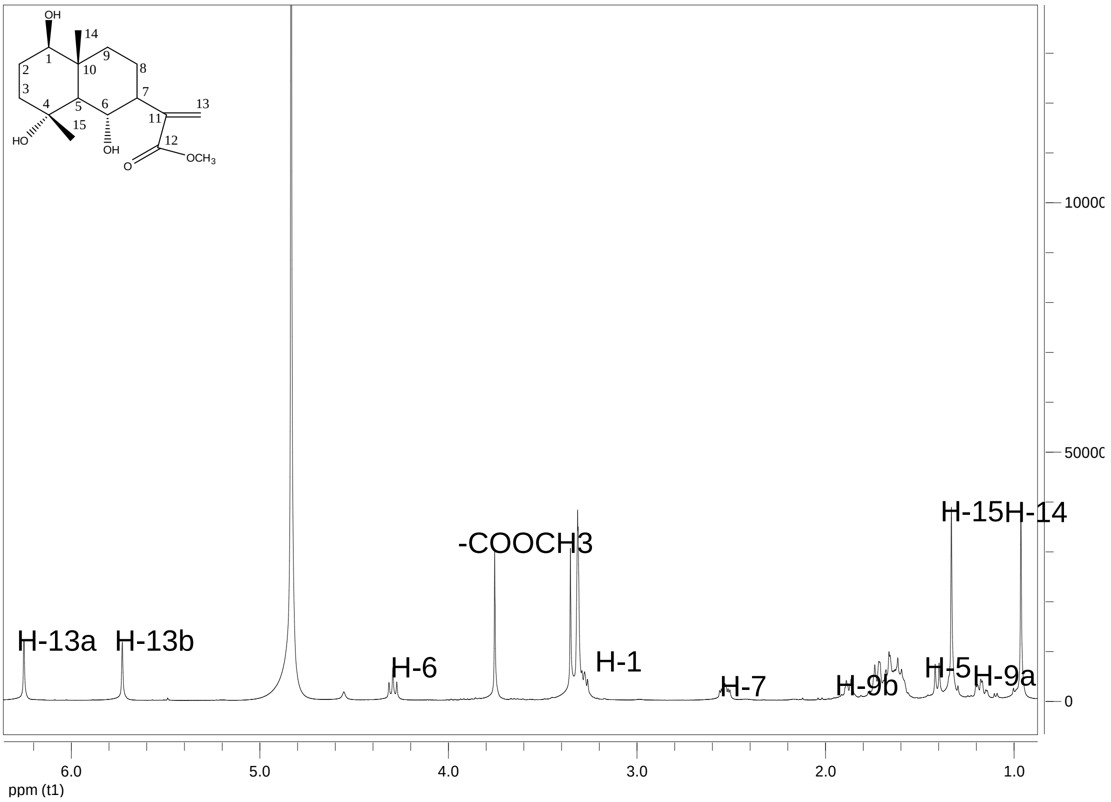

Figure S 53. ^{1}H-NMR spectrum of compound **10** (CD_3OD, 500MHz).

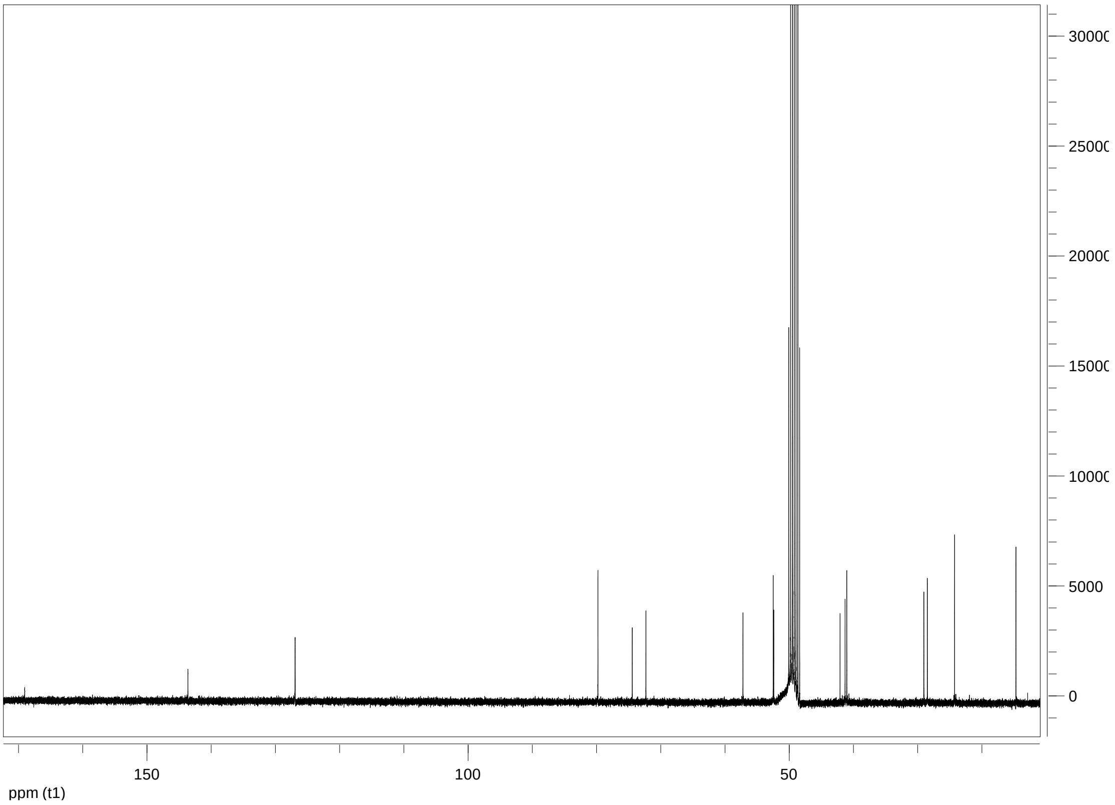

Figure S 54. ^{13}C-NMR spectrum of compound **10** (CD_3OD, 75MHz).

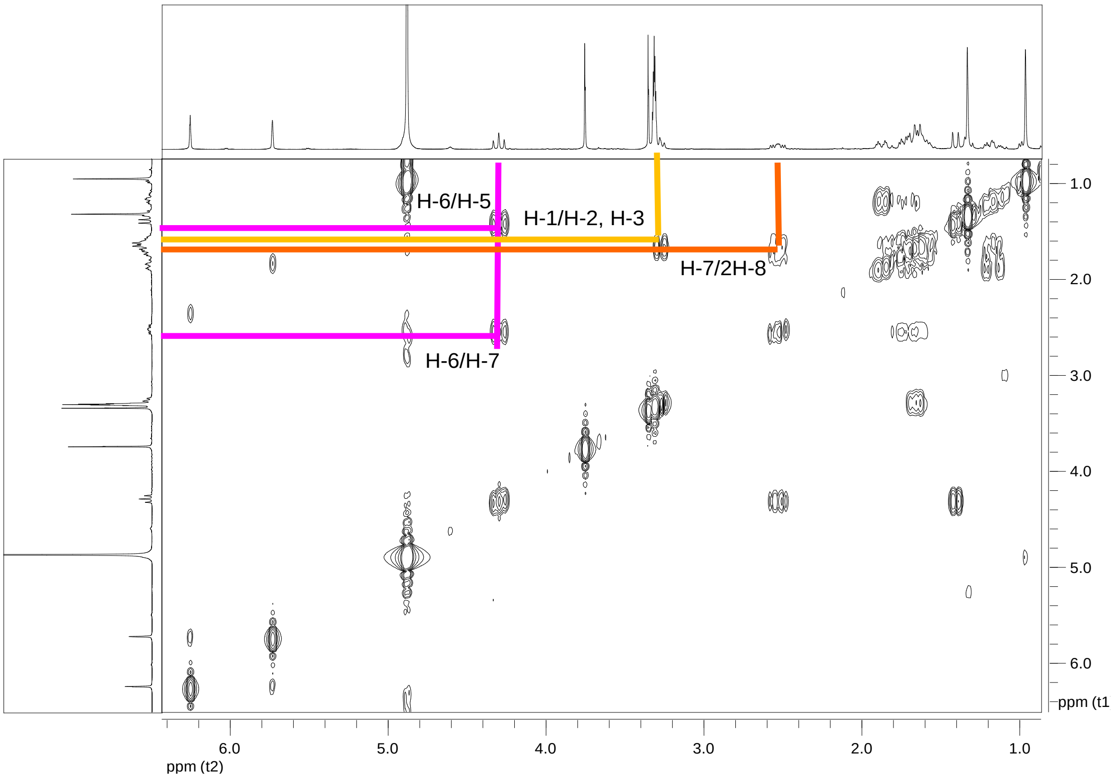

Figure S 55. COSY-NMR spectrum of compound **10** (CD_3OD, 300MHz).

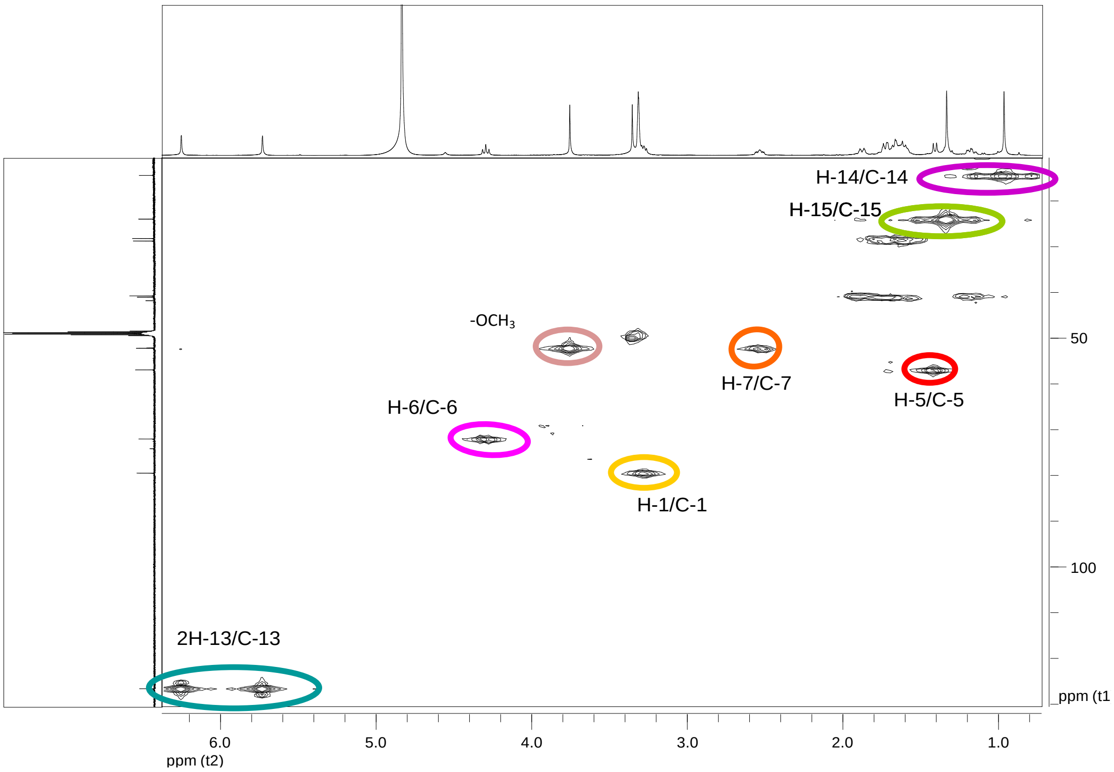

Figure S 56. HMQC-NMR spectrum of compound **10** (CD_3OD, 500MHz).

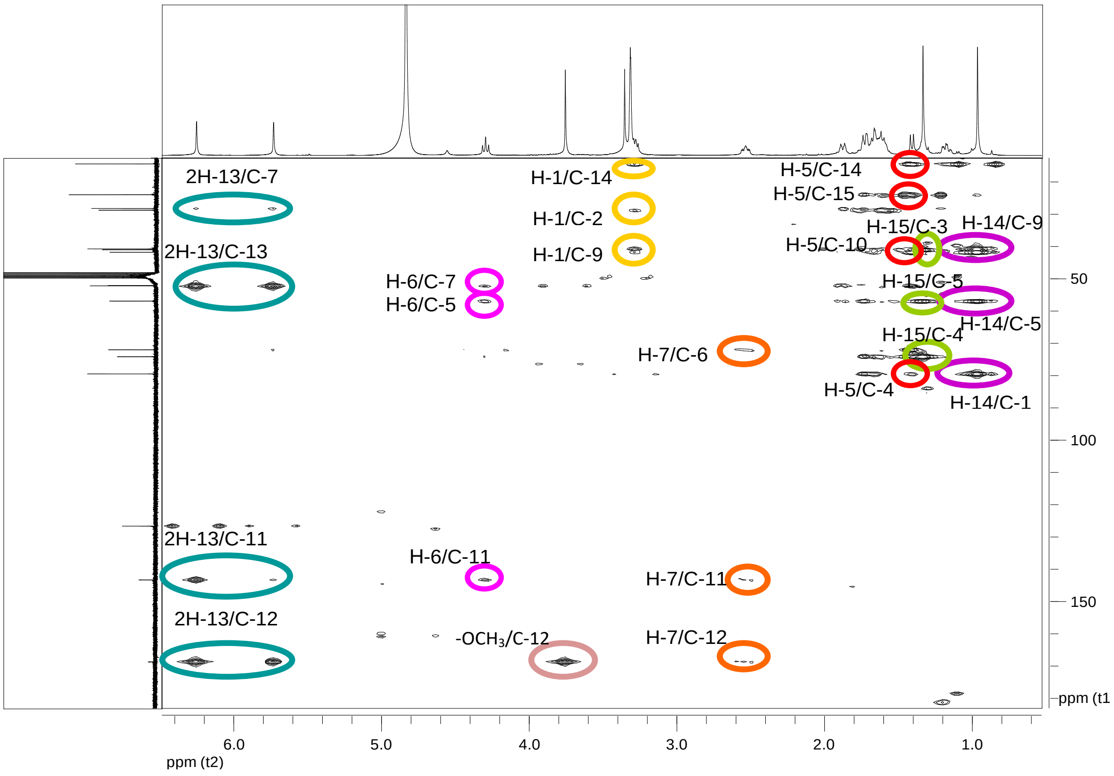

Figure S 57. HMBC-NMR spectrum of compound **10** (CD_3OD, 500MHz).

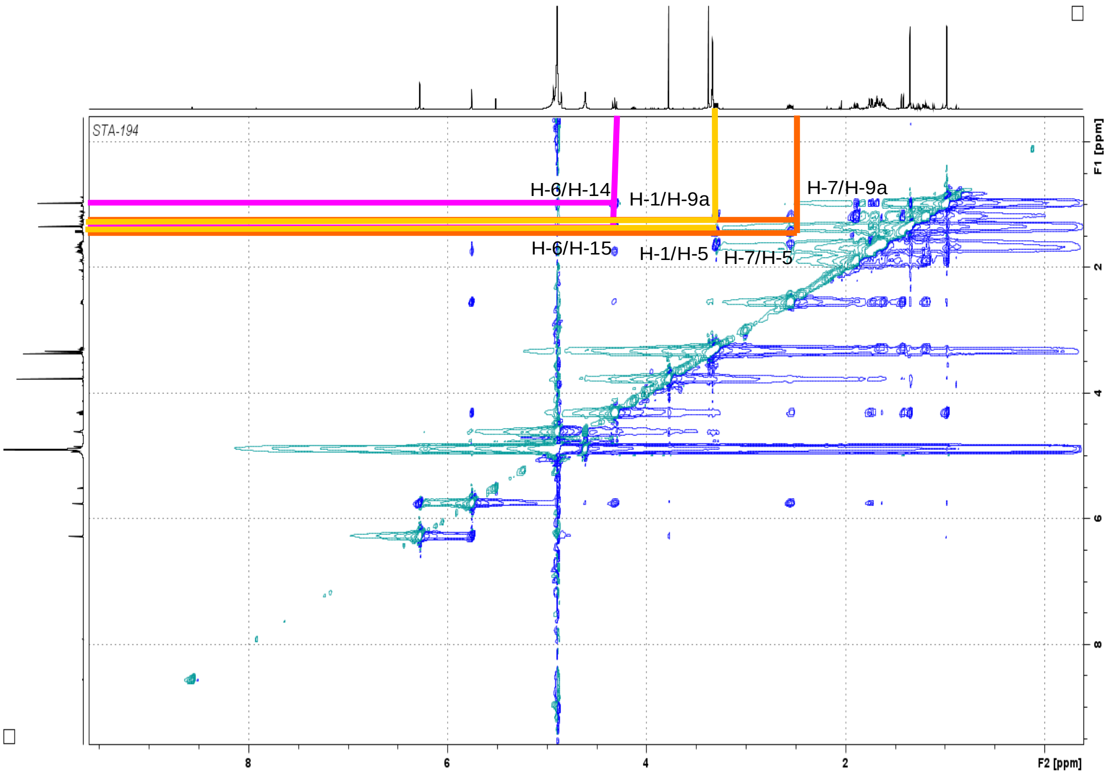

Figure S 58. NOESY-NMR spectrum of compound **10** (CD_3OD, 500MHz).

Table S13: ^{1}H and ^{13}C NMR of compound **11** ($CDCl_3$, 500MHz).

Position	δ_C ($CDCl_3$)	C	δ_H ($CDCl_3$)	Number H	Multiplicity J (Hz)
1	78.2	CH	3.49	1	*m*
2	31.6	CH_2	1.88	2	*br d* (J=5.5)
			1.58		*m*
3	34.9	CH_2	2.35	2	*dd* (J=4.0, 12.5)
			2.07		*t* (J=7.5)
4	145.0	C	-	-	-
5	55.0	CH	1.83	1	*d* (J=10.0)
6	67.6	CH	4.13	1	*t* (J=10.3)
7	57.4	CH	2.44	1	*t* (J=10.5)
8	67.2	CH	4.05	2	*td* (J=4.5, 10.8)
9	45.1	CH_2	2.35	2	*dd* (J=4.0, 12.5)
			1.25		*br s*
10	41.2	C	-	-	-
11	138.4	C	-	-	-
12	167.8	C=O	-	-	-
13	128.0	CH_2	6.43, 5.82	2	*br s*
14	14.1	CH_3	0.82	3	*br s*
15	108.5	CH_2	5.07, 4.74	2	*br s*
-COOCH3	51.6	-COOCH3	3.80	3	*s*

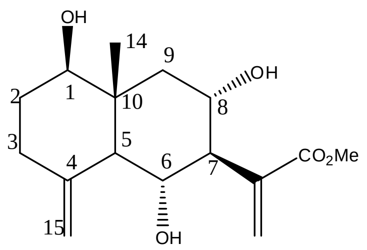

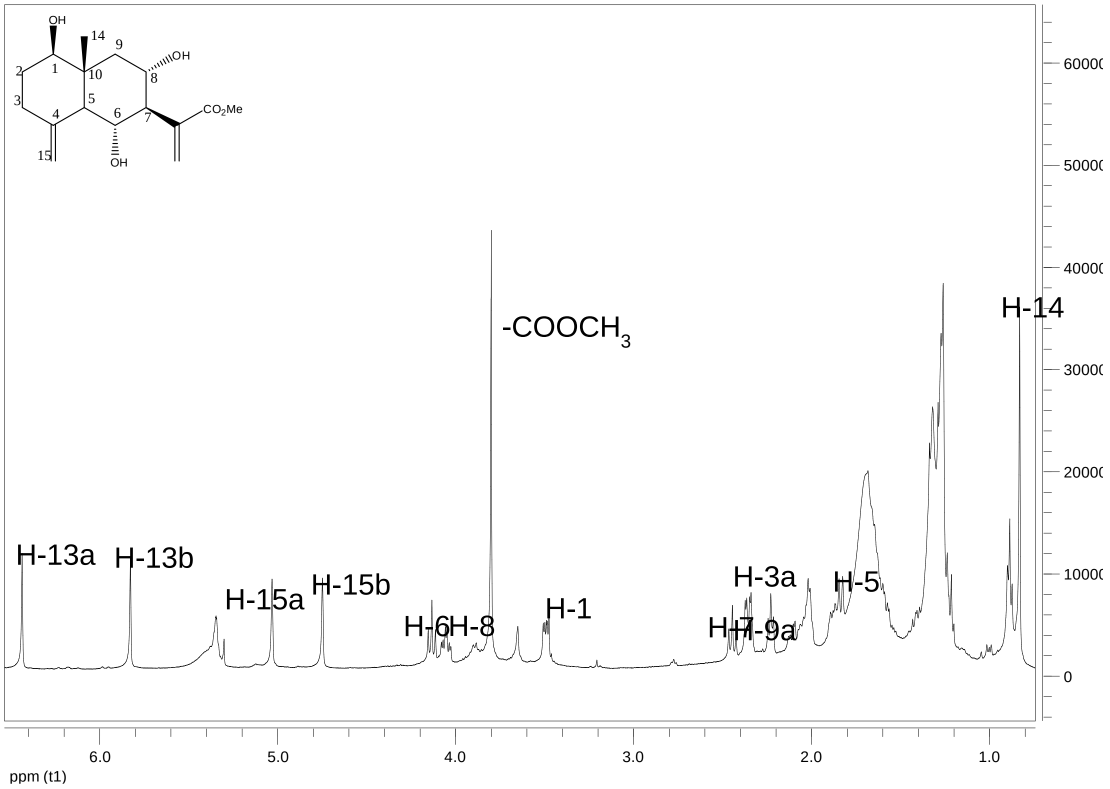

Figure S 59. ^{1}H-NMR spectrum of compound **11** ($CDCl_3$, 500MHz).

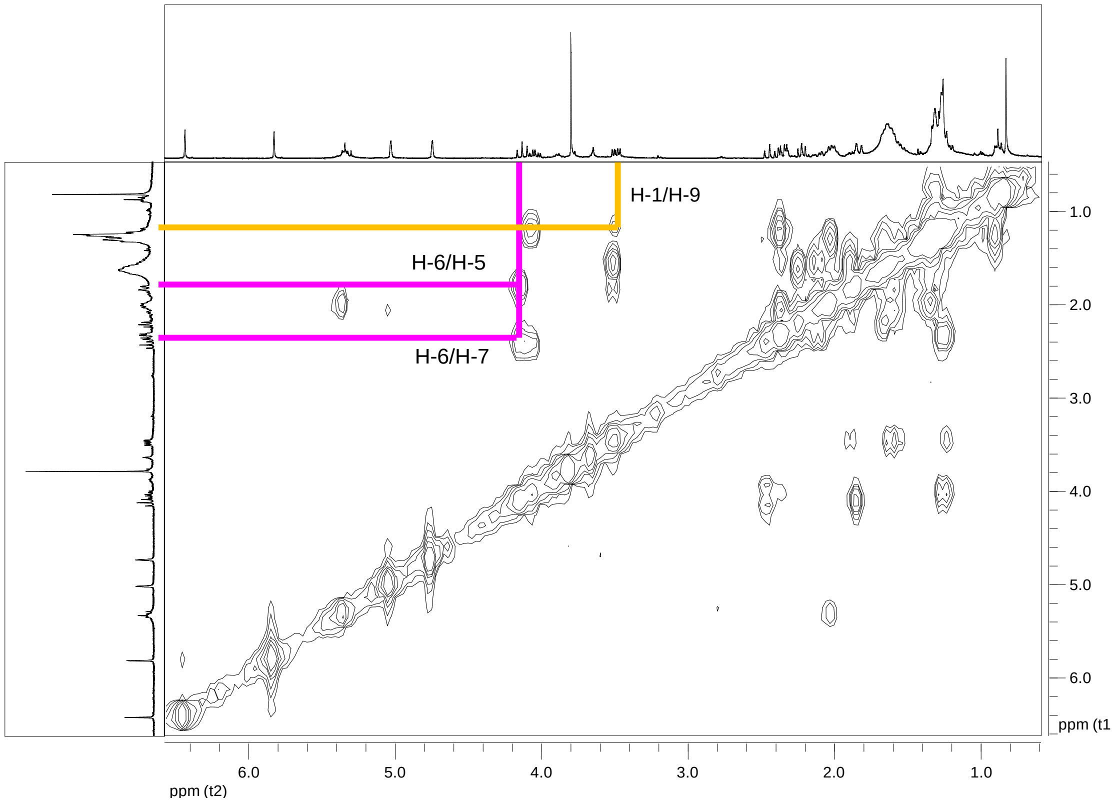

Figure S 60. COSY-NMR spectrum of compound **11** ($CDCl_3$, 300MHz).

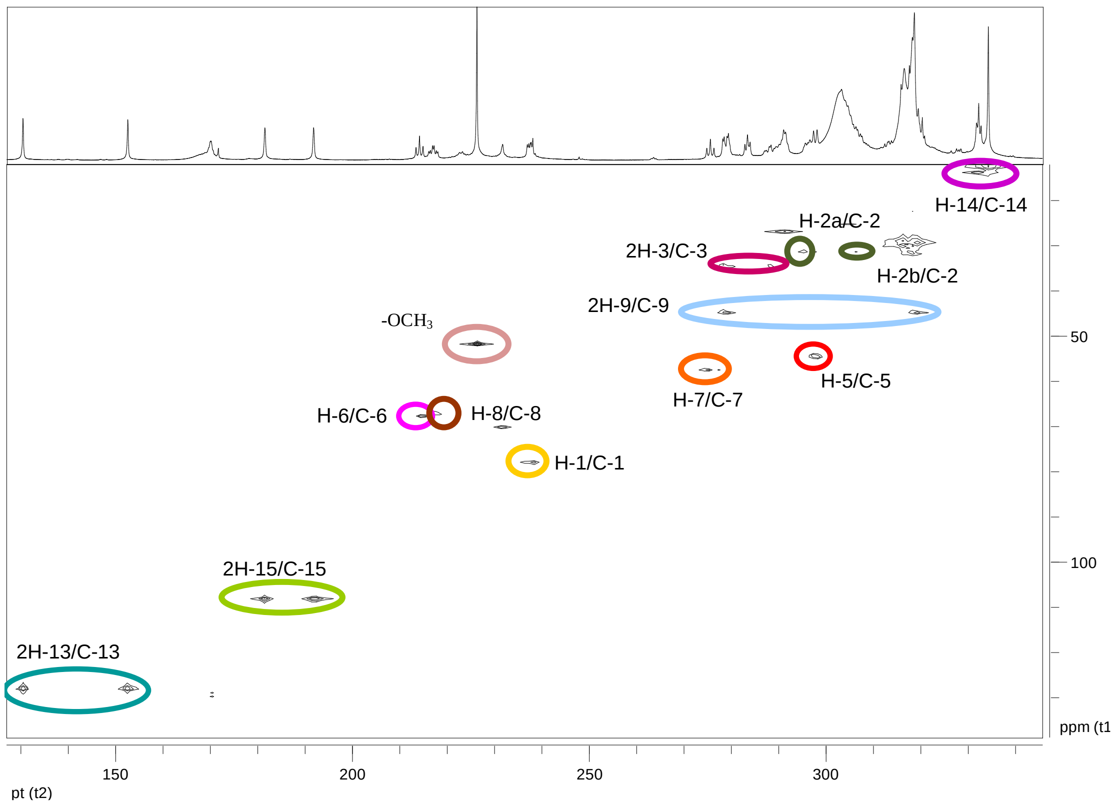

Figure S 61. HMQC-NMR spectrum of compound **11** ($CDCl_3$, 500MHz).

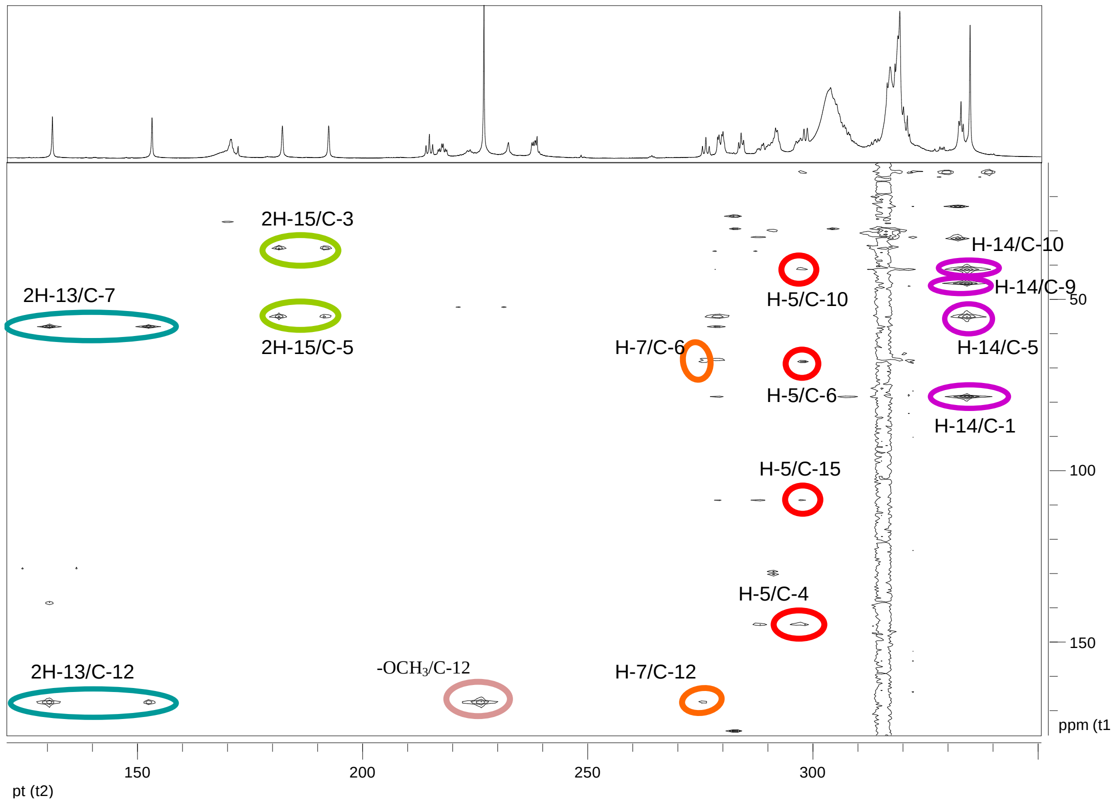

Figure S 62. HMBC-NMR spectrum of compound **11** ($CDCl_3$, 500MHz).

Table S14: ^{1}H NMR of compound **12** ($CDCl_3$, 300MHz).

**Position**	**δ_H ($CDCl_3$)**	**Number H**	**Multiplicity J (Hz)**
1, 5	3.10	2	*m*
2, 6	4.73	2	*d* (J=4.1)
4, 8	4.24	2	*dd* (J=6.9, 9.0)
4´, 8´	3.87	2	*dd* (J=3.8, 9.1)
a	6.90	2	*d* (J=8.1)
b	6.88	2	*d* (J=1.9)
c	6.81	2	*dd* (J=1.8, 8.1)
-OH	5.58	2	*s*
$-OCH_3$	3.88	6	*s*

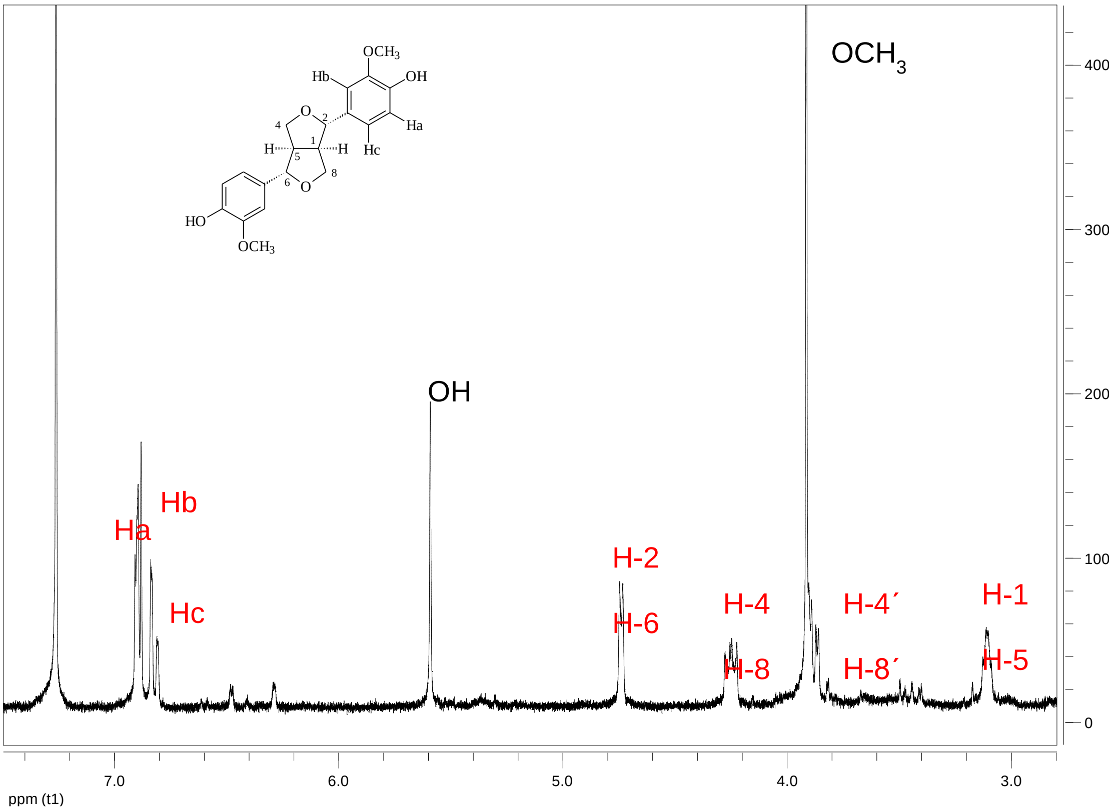

Figure S 63. ^{1}H-NMR spectrum of compound **12** ($CDCl_3$, 300MHz).

Table S15: ^{1}H NMR of compound **13** ($CDCl_3$, 500MHz).

Position	δ_H ($CDCl_3$)	Number H	Multiplicity J (Hz)
2a	1.97	1	*ddd* (J=2.5, 2.8, 14.5)
2b	1.53	1	*dd* (J=3.8, 14.8)
3	4.33	1	*m*
4a	2.45	1	*ddd* (J=2.5, 2.8, 14.0)
4b	1.80	1	*dd* (J=4.2, 14.0)
7	5.69	1	*s*
9	1.27	9	*br s*
10	1.47	3	*br s*
11	1.78	3	*br s*

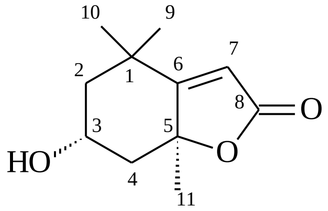

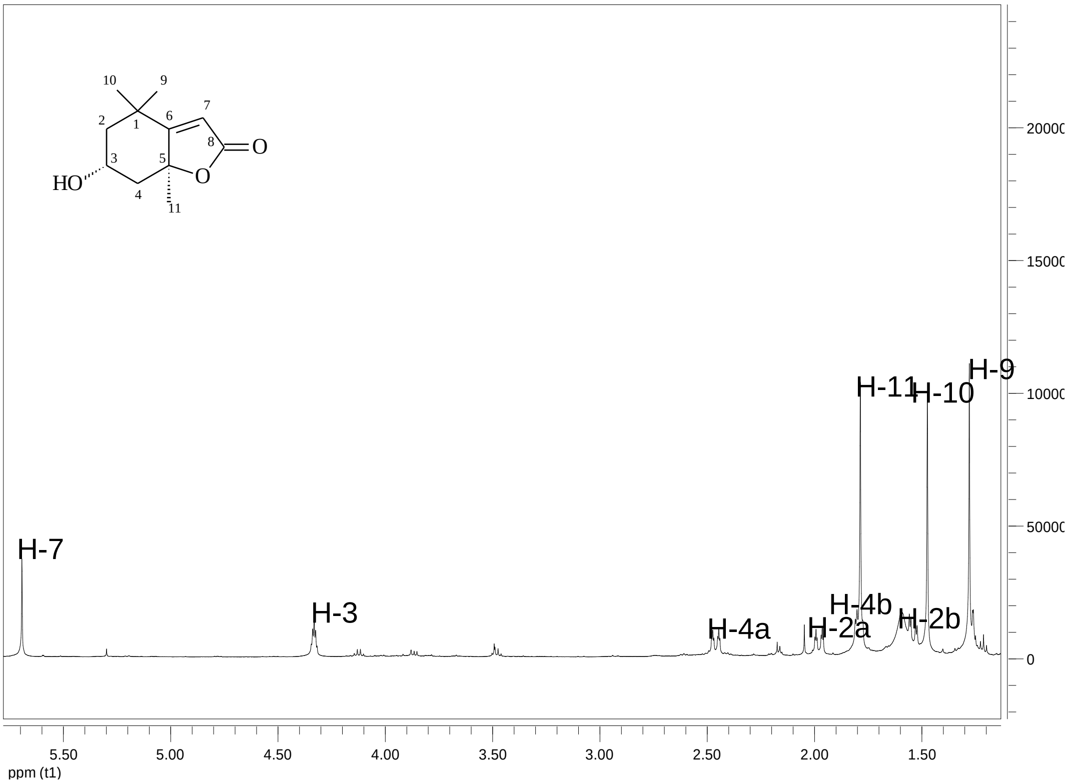

Figure S 64. ^{1}H-NMR spectrum of compound **13** ($CDCl_3$, 500MHz).

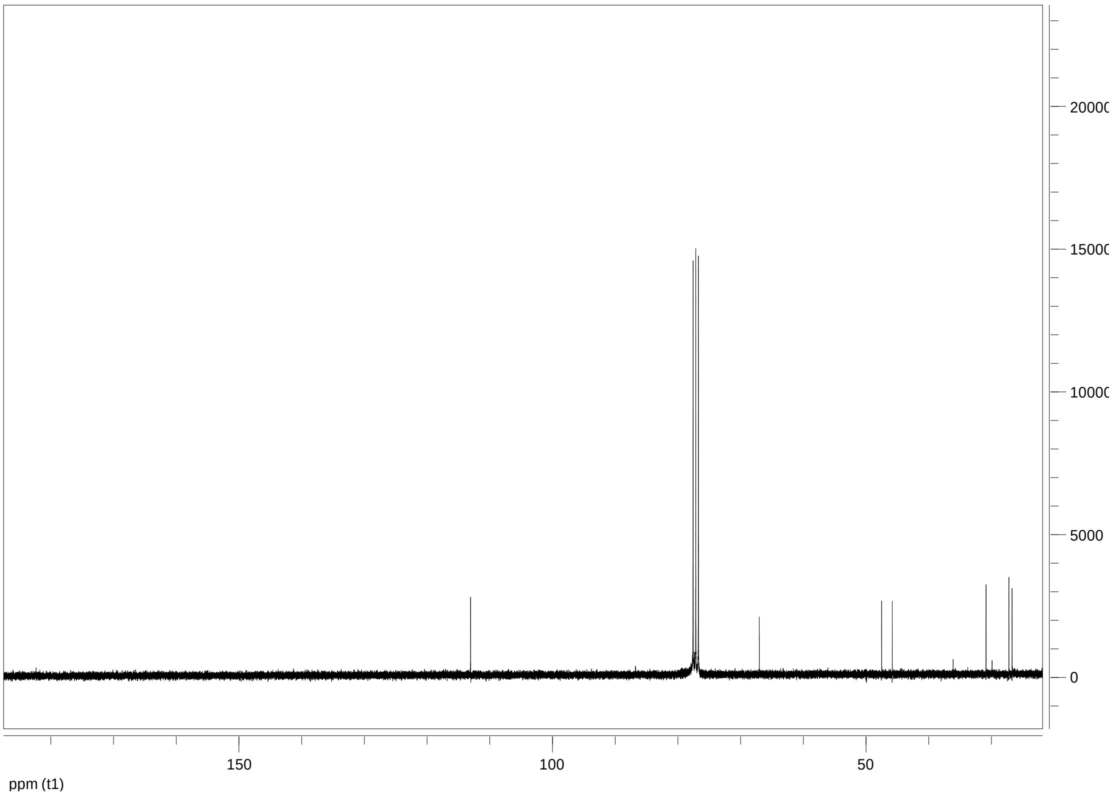

Figure S 65. ^{13}C-NMR spectrum of compound **13** ($CDCl_3$, 75MHz).

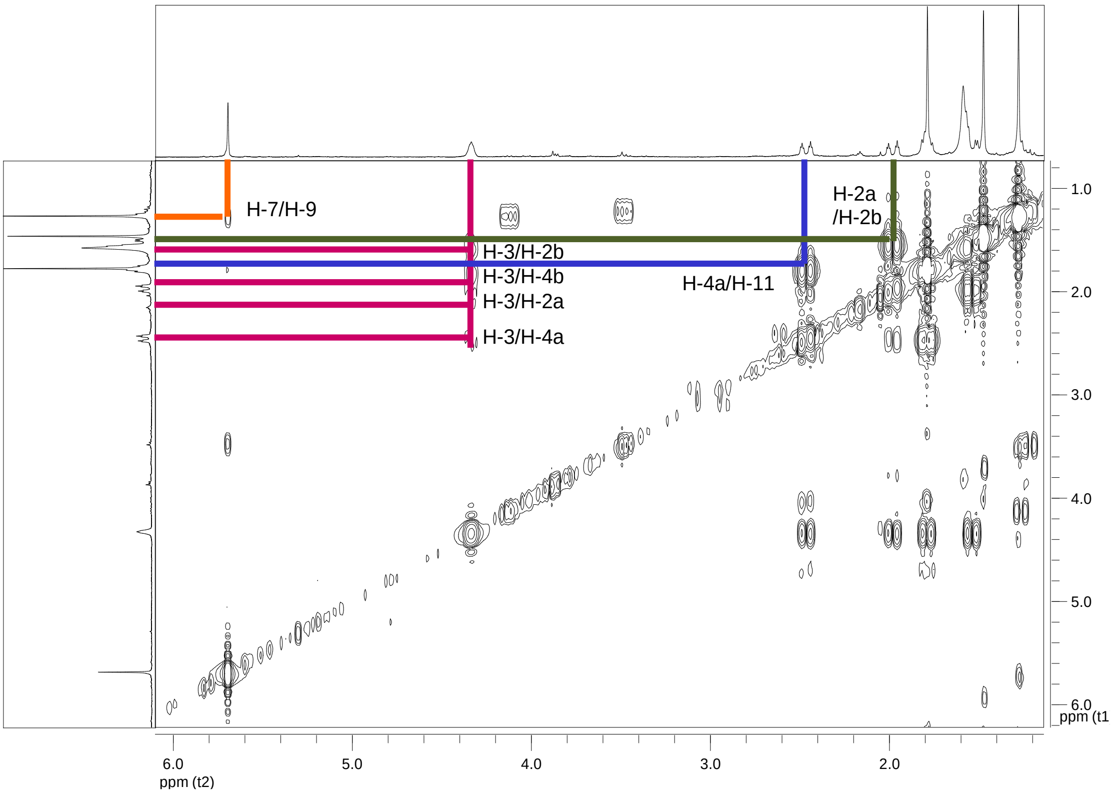

Figure S 66. COSY-NMR spectrum of compound **13** ($CDCl_3$, 300MHz).

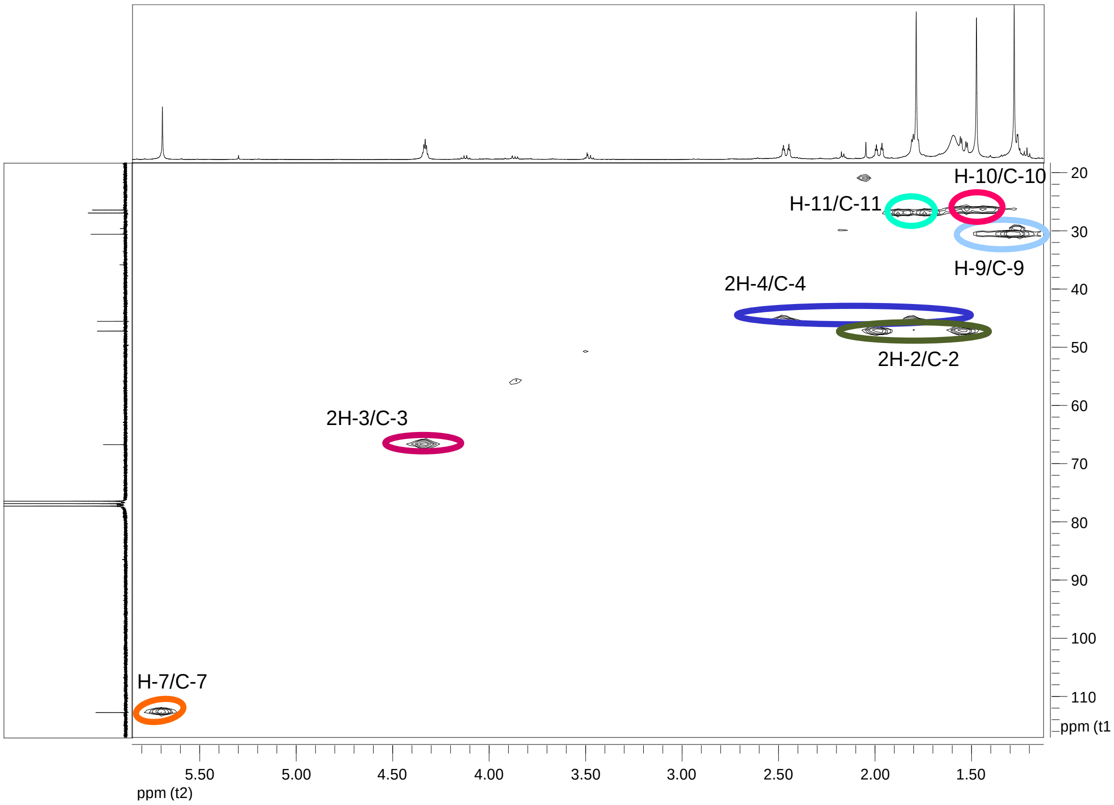

Figure S 67. HMQC-NMR spectrum of compound **13** ($CDCl_3$, 500MHz).

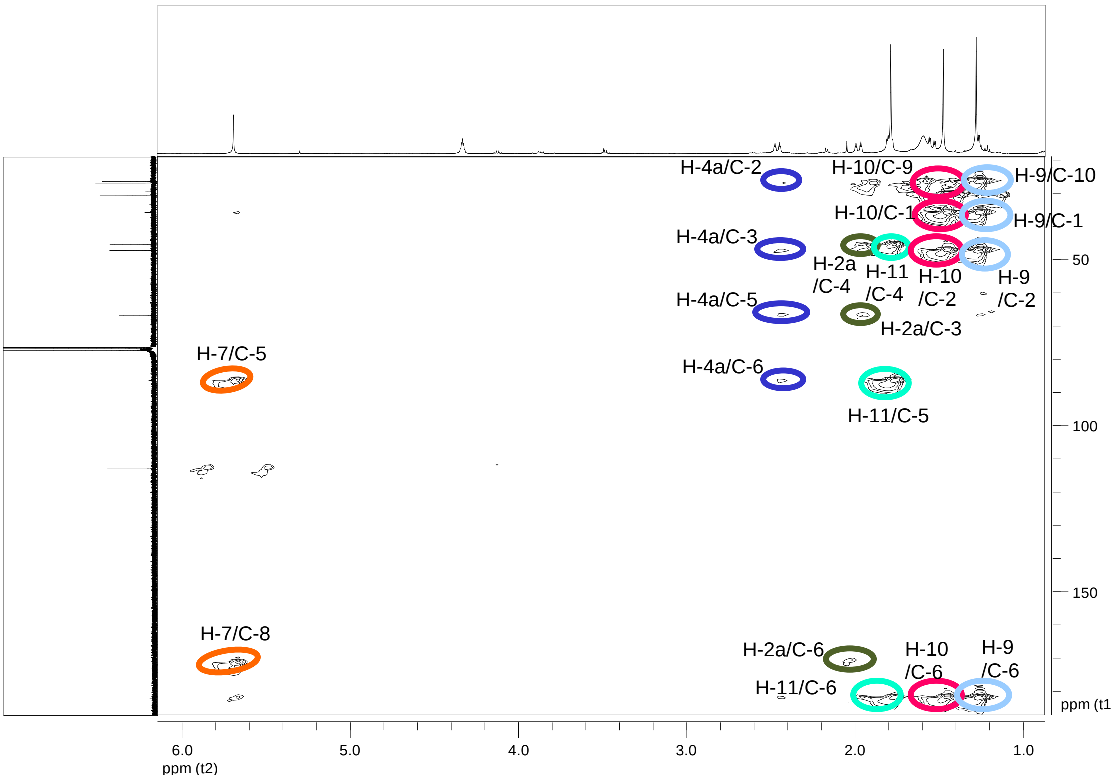

Figure S 68. HMBC-NMR spectrum of compound **13** ($CDCl_3$, 500MHz).

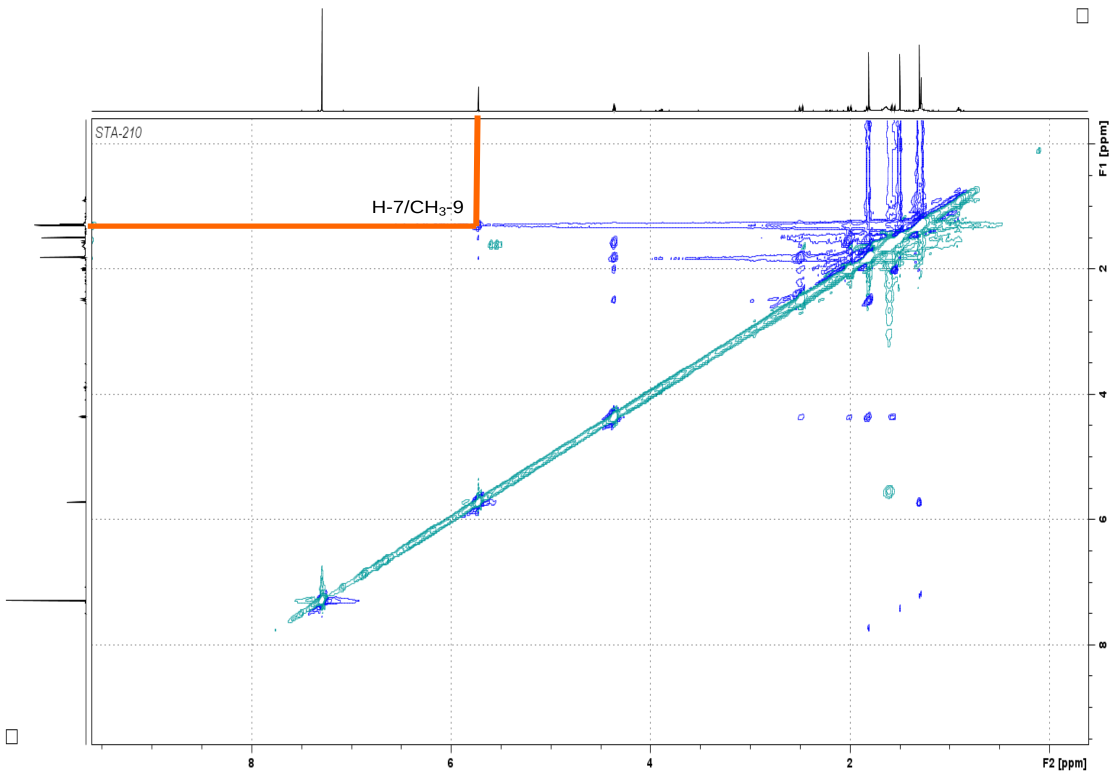

Figure S 69. NOESY-NMR spectrum of compound **13** ($CDCl_3$, 500MHz).

Table S16: ^{1}H NMR of compound **14** (CD_3OD, 300MHz).

Position	δ_H (CD_3OD)	Number H	Multiplicity J (Hz)
2′, 6′	7.81	2	*d* (J=8.7)
3′, 5′	6.90	2	*d* (J=9.0)
3	6.37	1	*s*
8	6.48	1	*s*
6-OCH_3	3.83	3	*br s*

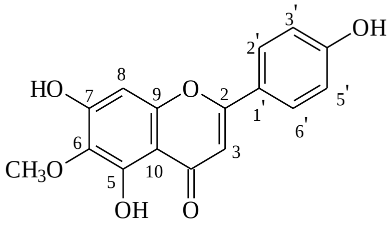

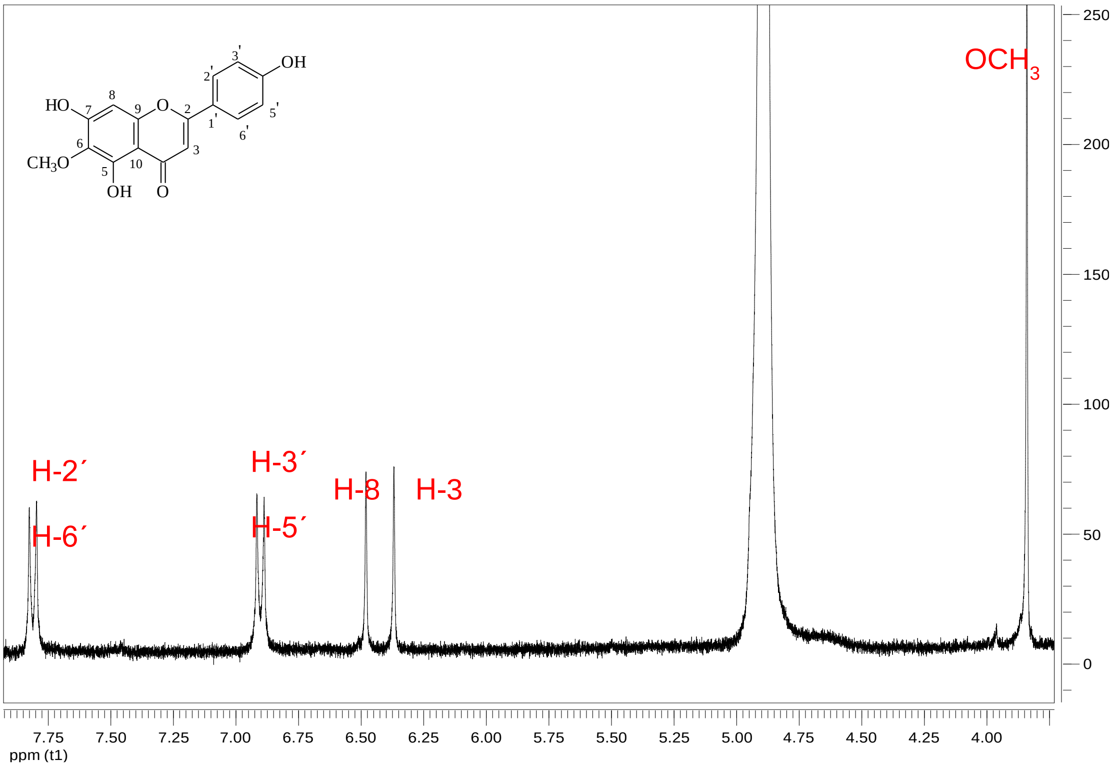

Figure S 70. ^{1}H-NMR spectrum of compound **14** (CD_3OD, 300MHz).

Table S17: ^{1}H and ^{13}C NMR of compound **15** (CD_3OD, 300MHz).

**Position**	***δ*** δ_C **(CD_3OD)**	***C***	δ_H **(CD_3OD)**	**Number H**	**Multiplicity J (Hz)**
2	166.3	C	-	-	-
3	95.6	CH	6.52	1	*br s*
4	184.1	C	-	-	-
5	154.8	C	-	-	-
6	133.2	C	-	-	-
7	160.0	C	-	-	-
8	103.3	CH	6.52	1	*br s*
9	153.9	C	-	-	-
10	105.3	C	-	-	-
1′	123.7	C	-	-	-
2′	114.1	CH	7.37	1	*br s*
3′	147.1	C	-	-	-
4′	151.1	C	-	-	-
5′	116.8	CH	6.89	1	*d* (J=7.5)
6′	120.3	CH	7.37	1	*br s*
-OCH_3	60.9	-OCH_3	3.87	3	*s*

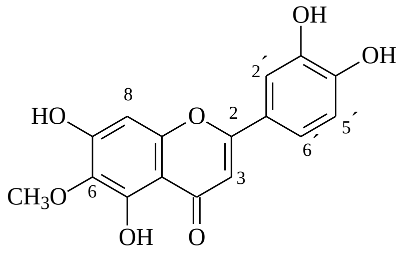

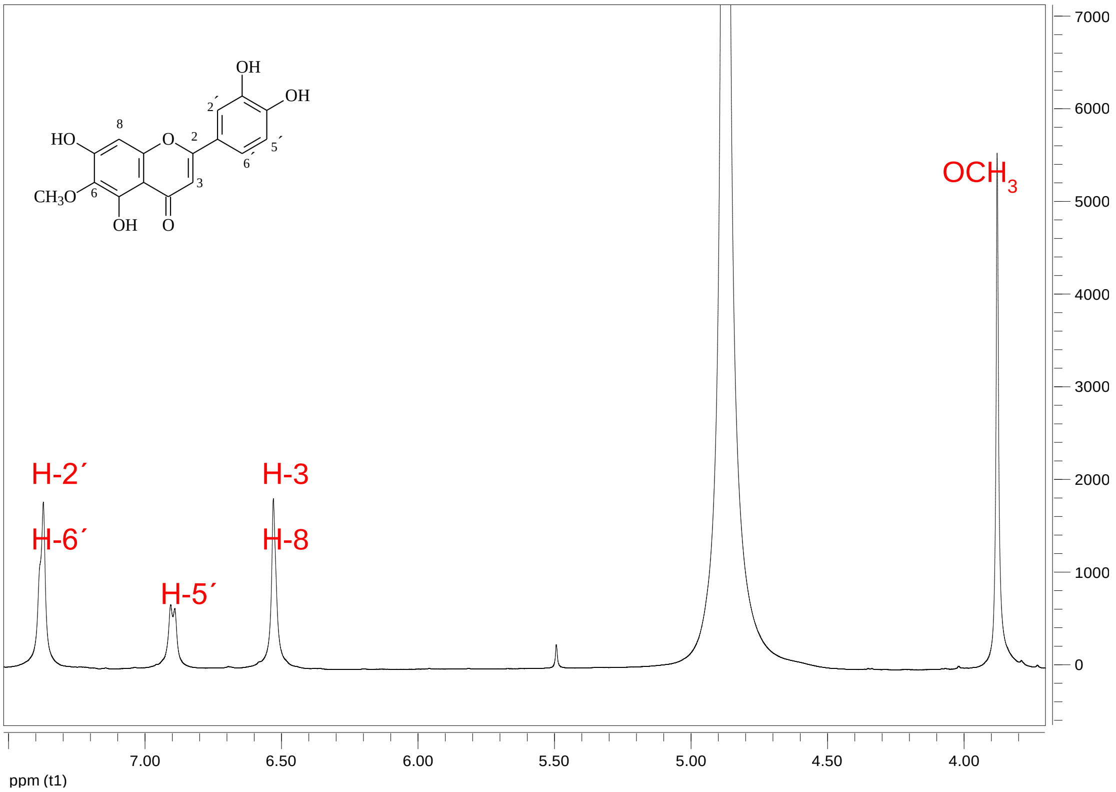

Figure S 71. ^{1}H-NMR spectrum of compound **15** (CD_3OD, 300MHz).

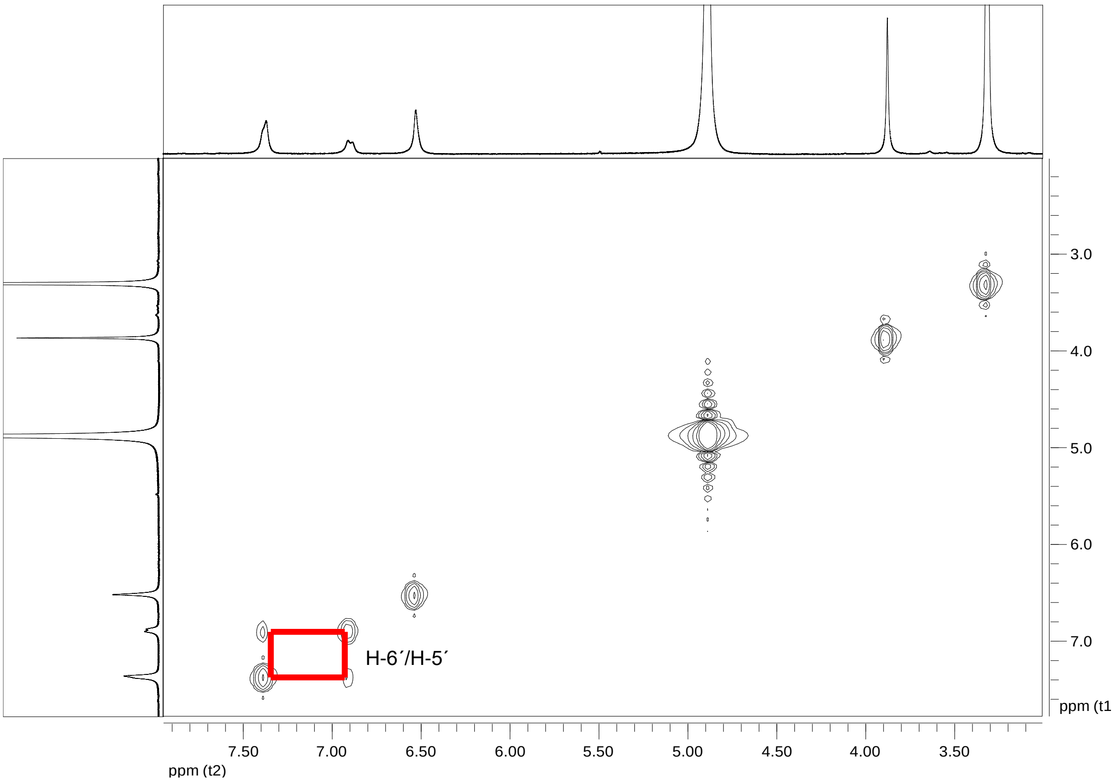

Figure S 72. COSY-NMR spectrum of compound **15** (CD_3OD, 500MHz).

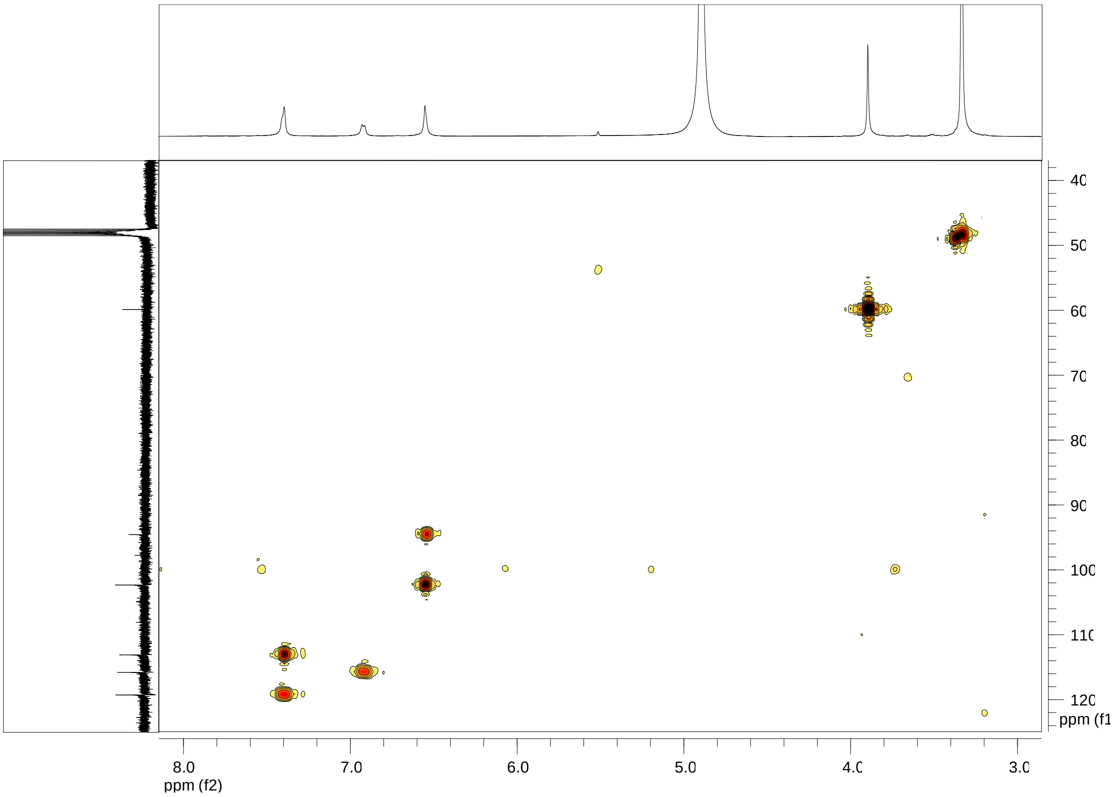

Figure S 73. HMQC-NMR spectrum of compound **15** (CD_3OD, 500MHz).

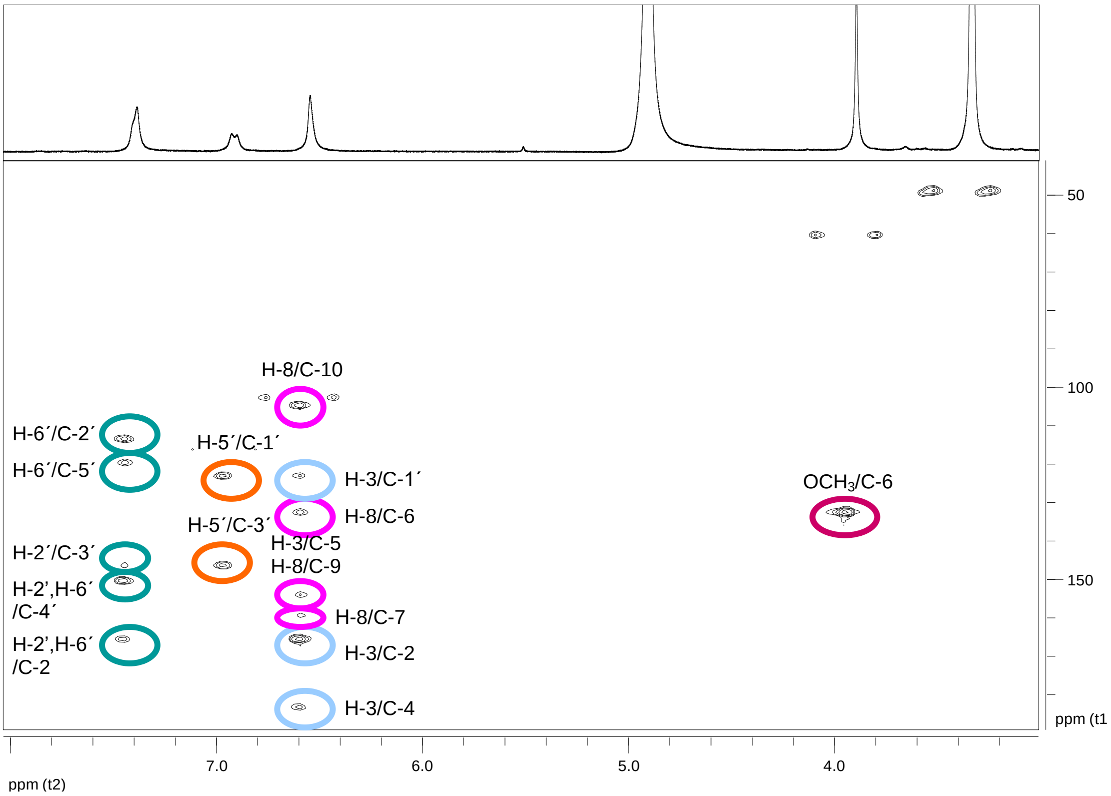

Figure S 74. HMBC-NMR spectrum of compound **15** (CD_3OD, 500MHz).

Table S18: ^{1}H and ^{13}C NMR of compound **16** ($CDCl_3$, 300MHz).

Position	δ_C ($CDCl_3$)	*C*	δ_H ($CDCl_3$)	Number H	Multiplicity J (Hz)
2	163.9	C	-	-	-
3	102.9	CH	6.55	1	*s*
4	182.2	C	-	-	-
5	152.5	C	-	-	-
6	131.9	C	-	-	-
7	158.5	C	-	-	-
8	91.3	CH	6.60	1	*s*
9	152.1	C	-	-	-
10	110.1	C	-	-	-
1′	121.5	C	-	-	-
2′	105.1	CH	7.33	1	*d* (J=1.8)
3′	148.0	C	-	-	-
4′	150.8	C	-	-	-
5′	115.8	CH	7.03	1	*d* (J=8.4)
6′	120.4	CH	7.47	1	*dd* (J=1.8, 8.4)
$-OCH_3$	60.9	$-OCH_3$	4.04	3	*s*
$-OCH_3$	56.2	$-OCH_3$	4.00	3	*s*

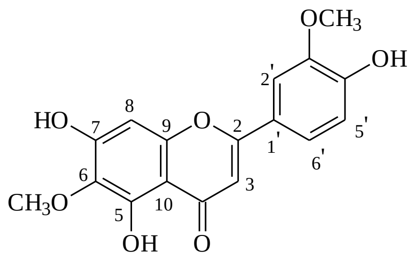

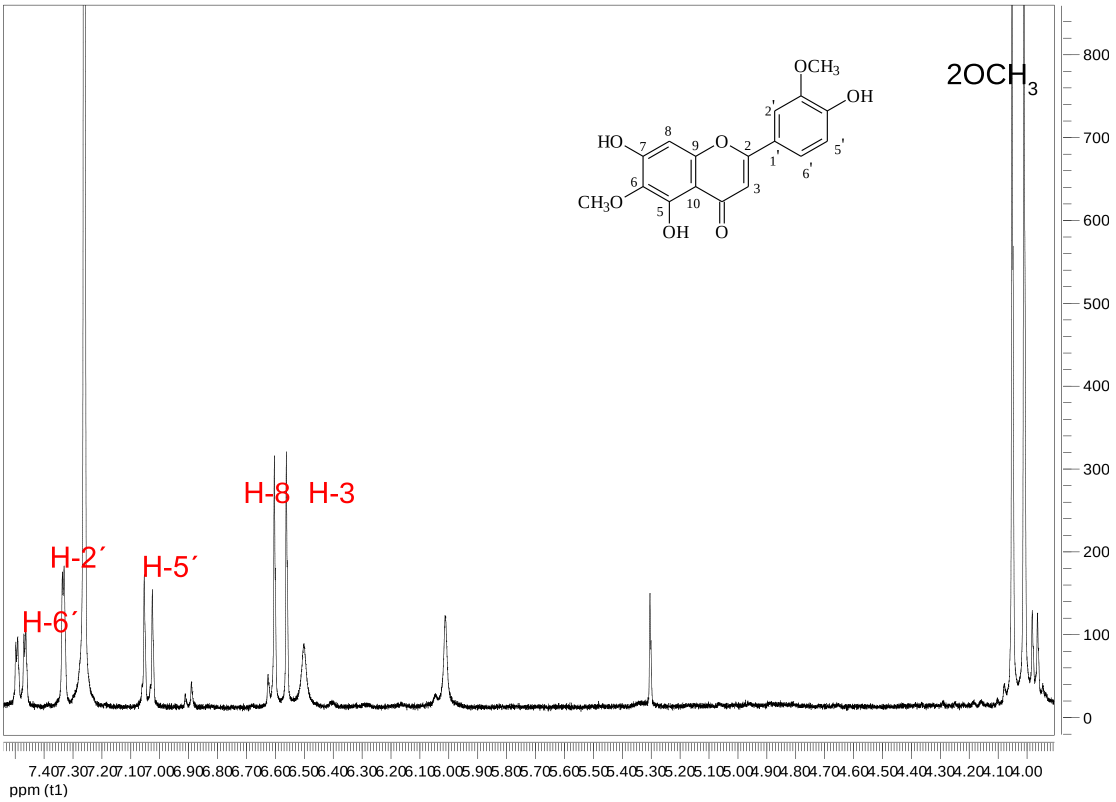

Figure S 75. ^{1}H-NMR spectrum of compound **16** ($CDCl_3$, 300MHz).

Table S19: ^{1}H NMR of compound **17** (CD_3OD, 300MHz).

**Position**	**δ_H (CD_3OD)**	**Number H**	**Multiplicity J (Hz)**
2	5.26	2	*dd* (J=3.3, 12.6)
3axial	3.05	1	*dd* (J=12.6, 16.8)
3eq	2.67	1	*dd* (J=3.6, 16.8)
6, 8	5.85	2	*d* (J=0.3)
2′	6.91	1	*d* (J=1.8)
5′, 6′	6.78	2	*br s*

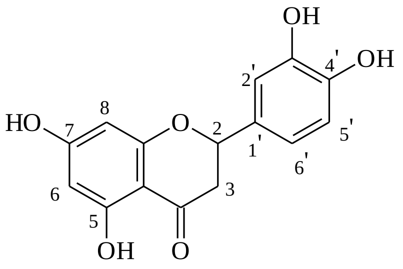

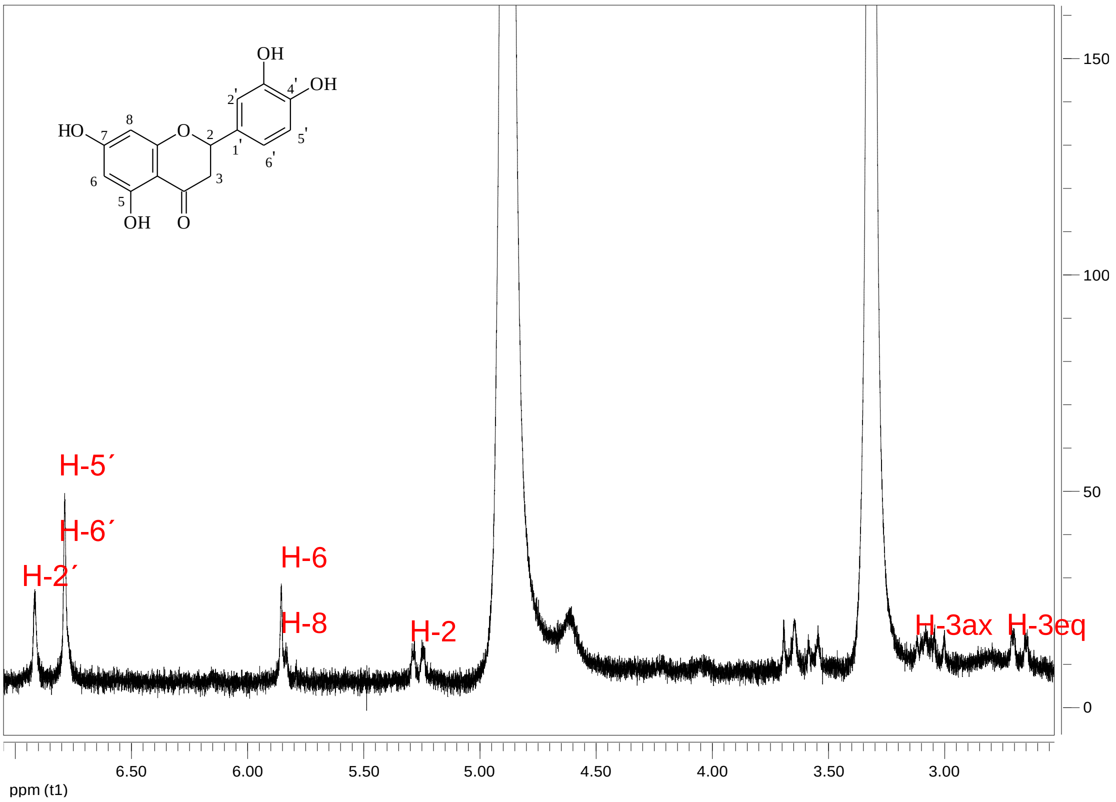

Figure S 76. ^{1}H-NMR spectrum of compound **17** (CD_3OD, 300MHz).

Table S20: ^{1}H NMR of compound **18** (CD_3OD, 600MHz).

δ (ppm)	nºH	Multiplicity J (Hz)	Position
7.35	1	*d* (J=1.8)	H-2´
7.05	1	*dd* (J=1.8, 7.8)	H-6´
6.89	1	*d* (J=8.4)	H-5´
5.91	1	*d* (J=1.8)	H-8
5.88	1	*d* (J=1.8)	H-6
5.34	1	*dd* (J=2.4, 12.6)	H-2
4.81	1	*d* (J=6.9)	H-1´´
3.91	1	*dd* (J=1.8, 12.0)	H-6´´a
3.69	1	*dd* (J=6.0, 12.0)	H-6´´b
3.49	1	m	H-2´´
3.47	1	m	H-3´´
3.42	1	m	H-4´´
3.38	1	m	H-5´´
3.14	1	*dd* (J=12.6, 16.8)	H-3a
2.71	1	*dd* (J=3.0, 16.8)	H-3b

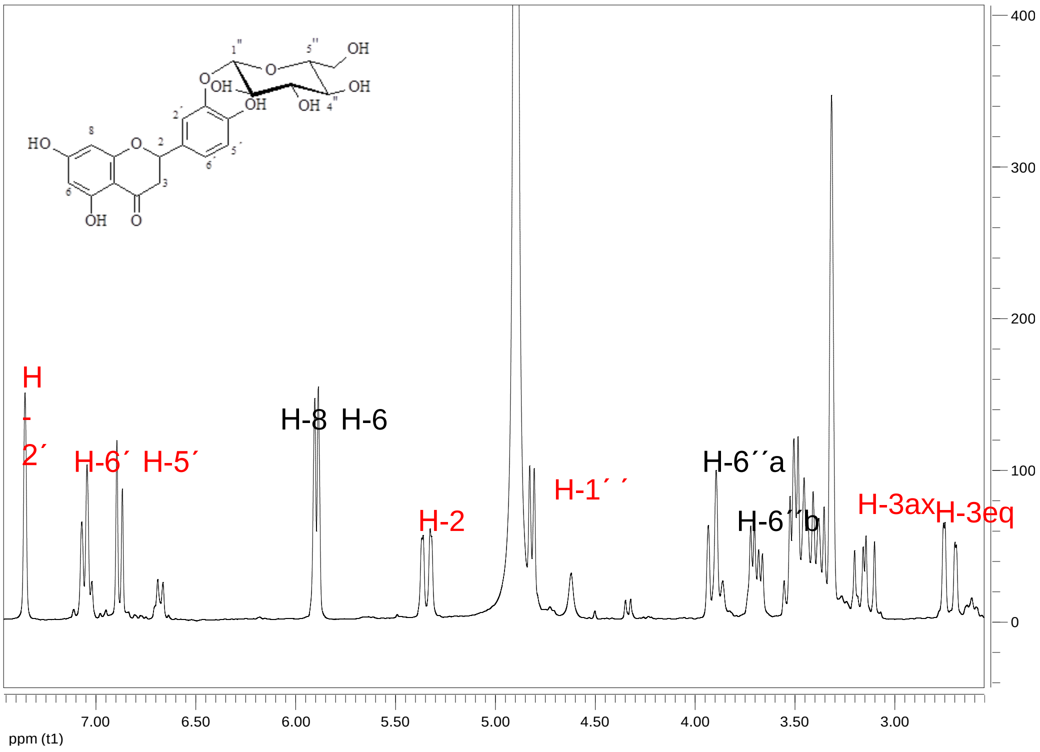

Figure S 77. ^{1}H-NMR spectrum of compound **18** (CD_3OD, 600MHz).

Table S21: ^{13}C NMR of compound **18** (from HMQC and HMBC spectra).

δ **(ppm)**	**Position**
195.8	C-4
163.8	C-5
147.1	C-4′
144.5	C-3′
130.2	C-1′
121.6	C-6′
115.8	C-5′
115.7	C-2′
102.5	C-1′′
94.6	C-8
78.7	C-2
76.7	C-4′′
75.9	C-3′′
72.8	C-2′′
69.7	C-5′′
61.1	C-6′′
42.3	C-3

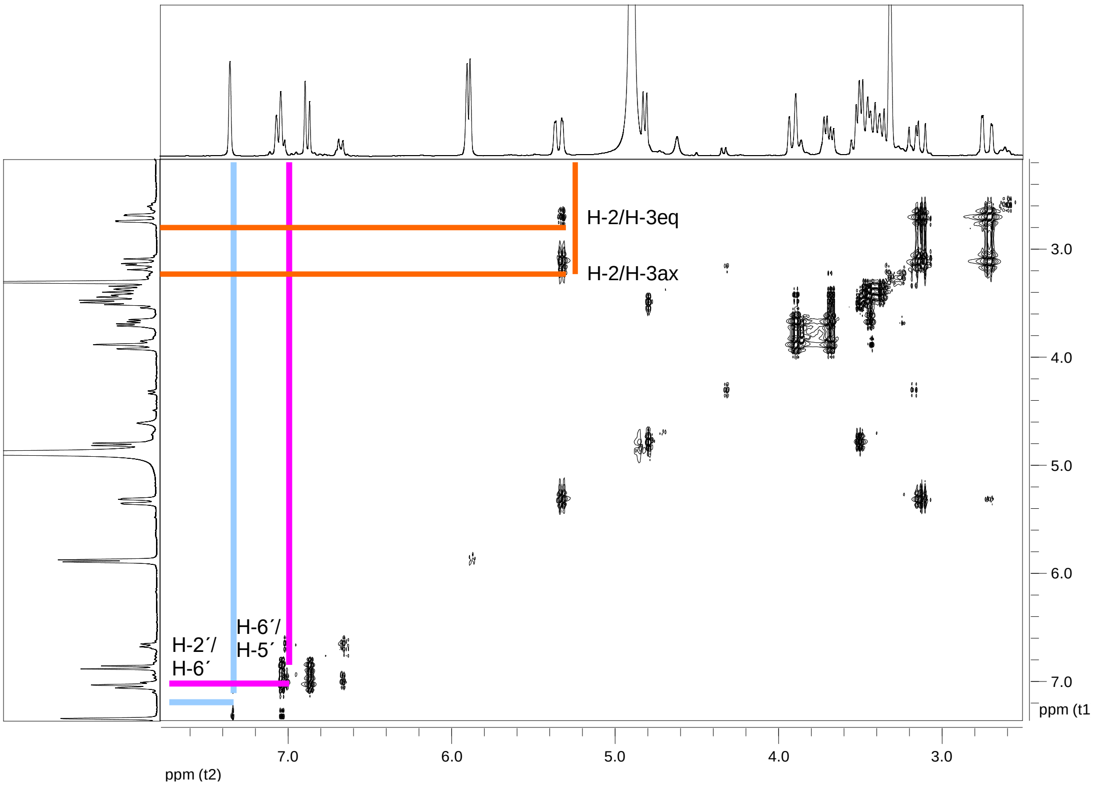

Figure S 78. COSY-NMR spectrum of compound **18** (CD_3OD, 600MHz).

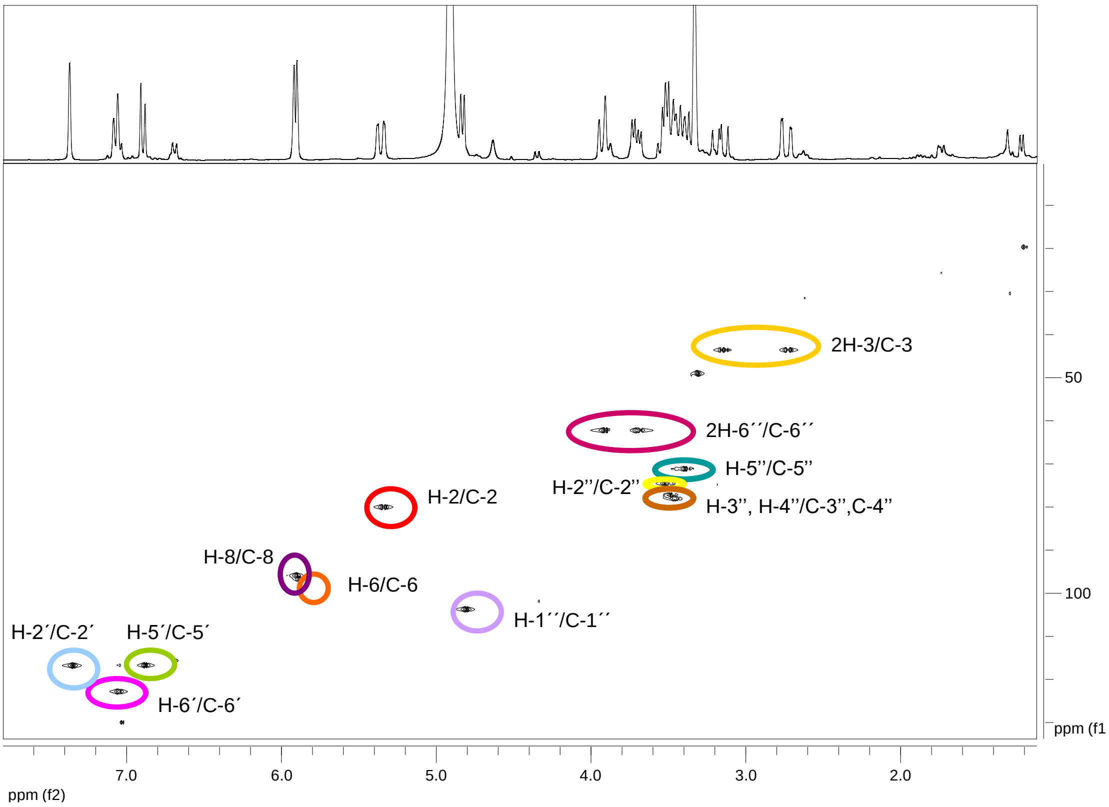

Figure S 79. HMQC-NMR spectrum of compound **18** (CD_3OD, 600MHz).

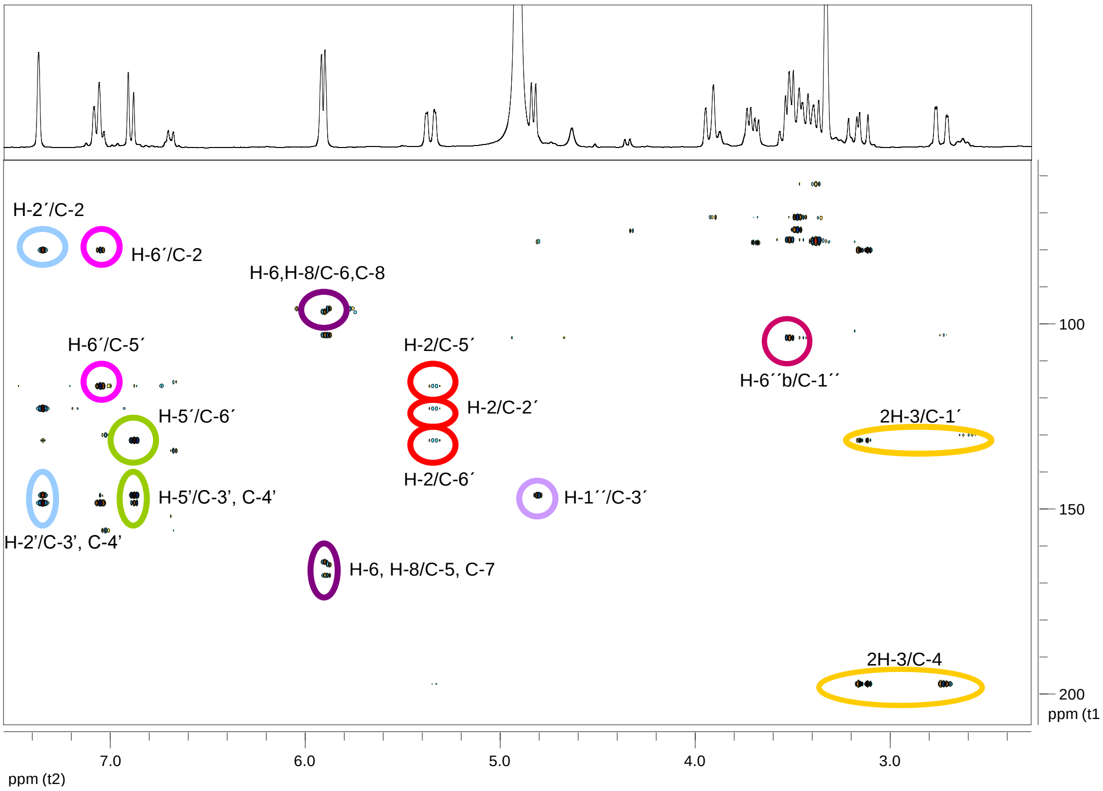

Figure S 80. HMBC-NMR spectrum of compound **18** (CD_3OD, 600MHz).

Table S22: ^{1}H NMR of compound **19** (CD_3OD, 500MHz).

δ (ppm)	nºH	Multiplicity J (Hz)	Position
6.93	1	*brs*	H-2´
6.78	2	*brs*	H-6´, H-5´
6.23	1	*brs*	H-6
6.19	1	*brs*	H-8
5.33	1	*dd* (J=2.9, 12.4)	H-2
5.00	1	*d* (J=6.8)	H-1´´
3.78	1	*d* (J =3.8)	H-5´´
3.48-3.51	3	*m*	H-2´´, H-3´´, H-4´´
3.12	1	*dd* (J=12.4, 17.1)	H-3ax
2.76	1	*dd* (J=2.9, 17.1)	H-3eq

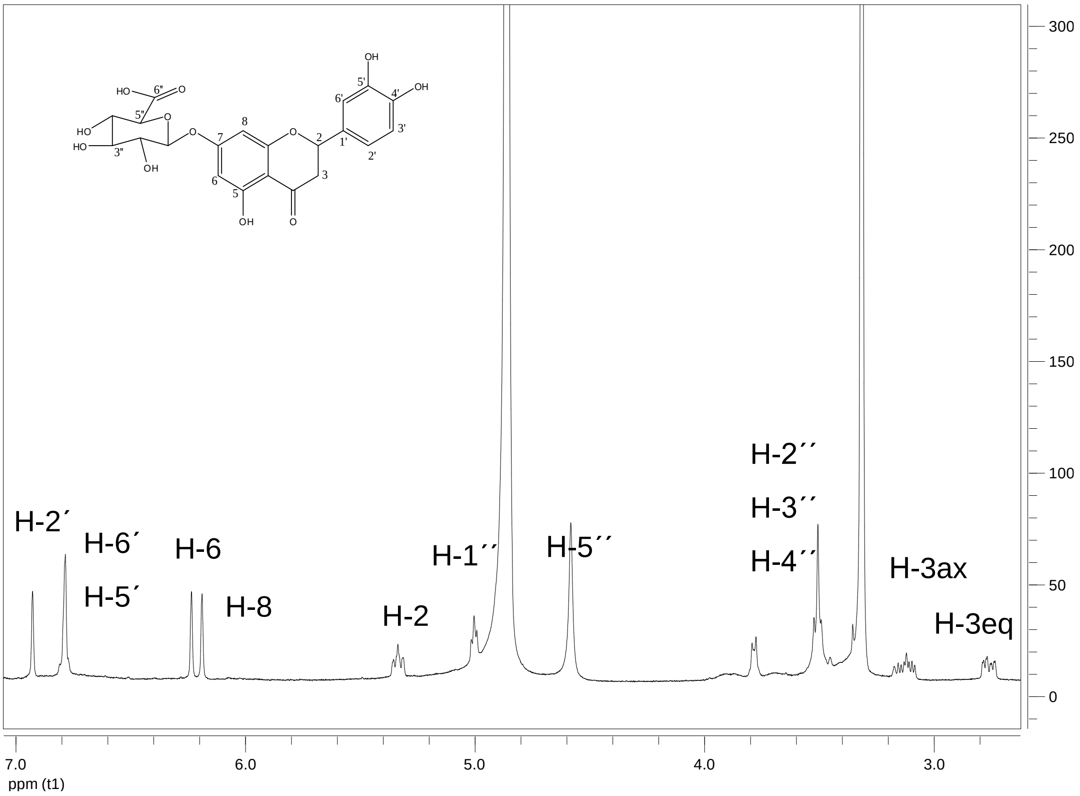

Figure S 81. ^{1}H-NMR spectrum of compound **19** (CD_3OD, 500MHz).

Table S23: ^{1}H NMR of compound **20** (CD_3OD, 300MHz).

δ (ppm)	nºH	Multiplicity J (Hz)	Position
7.43	1	*brs*	H-2
7.41	1	*brd* (J=9.0)	H-6
6.78	1	*d* (J=8.1)	H-5

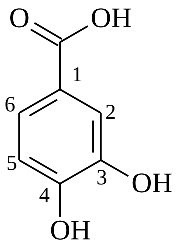

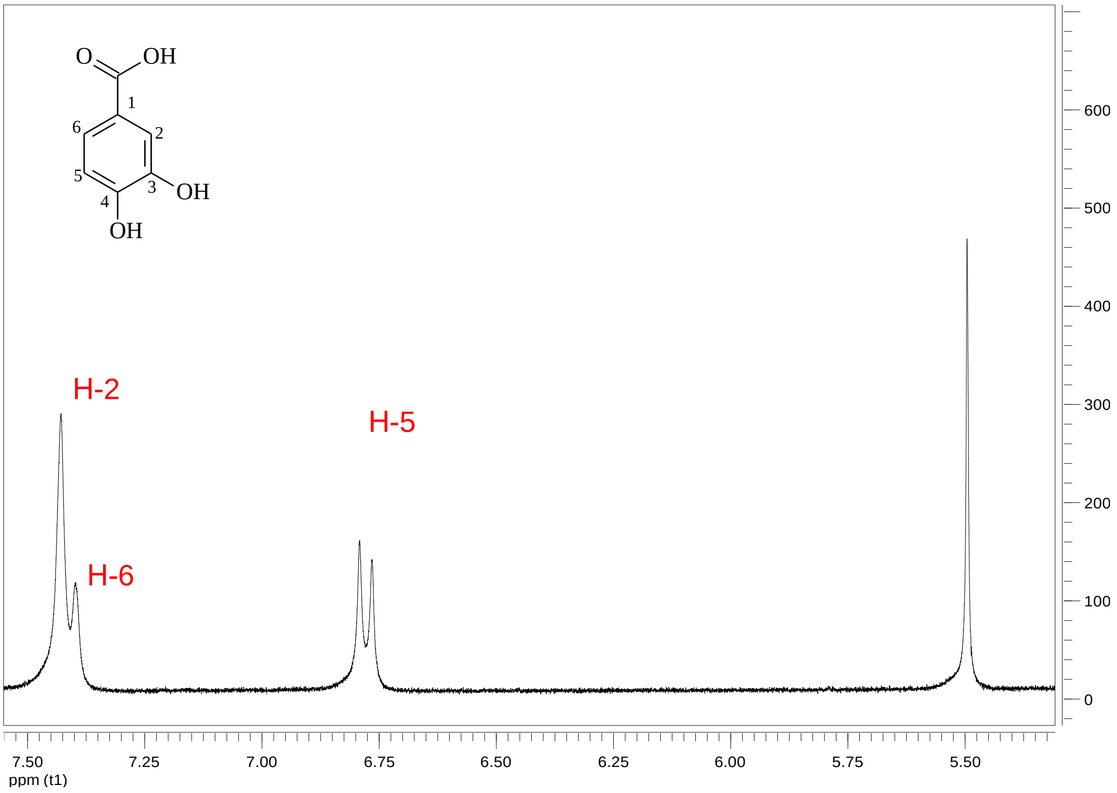

Figure S 82. ^{1}H-NMR spectrum of compound **20** (CD_3OD, 300MHz).

Table S24: ^{1}H NMR of compound **21** (CD_3OD, 600MHz).

δ (ppm)	nºH	Multiplicity, J(Hz)	Position
6.96	2	*d* (J=9.0)	H-2, H-6
6.69	2	*d* (J=9.0)	H-3, H-5
4.73	1	*d* (J=7.5)	H-1′
3.88	1	*brd* (J=12.0)	H-6′a
3.70	1	*dd* (J=4.2, 12.0)	H-6′b
3.44-3.37	4	*m*	H-2′, H-3′, H-4′, H-5′

Table S25: ^{13}C NMR of compound **21** (from HMQC and HMBC spectra).

δ (ppm)	Position
152.3	C-4
150.9	C-1
119.4	C-6
119.2	C-2
116.6	C-3
116.6	C-5
103.3	C-1′
77.7	C-3′
77.7	C-5′
74.7	C-2′
71.4	C-4′
62.5	C-6′

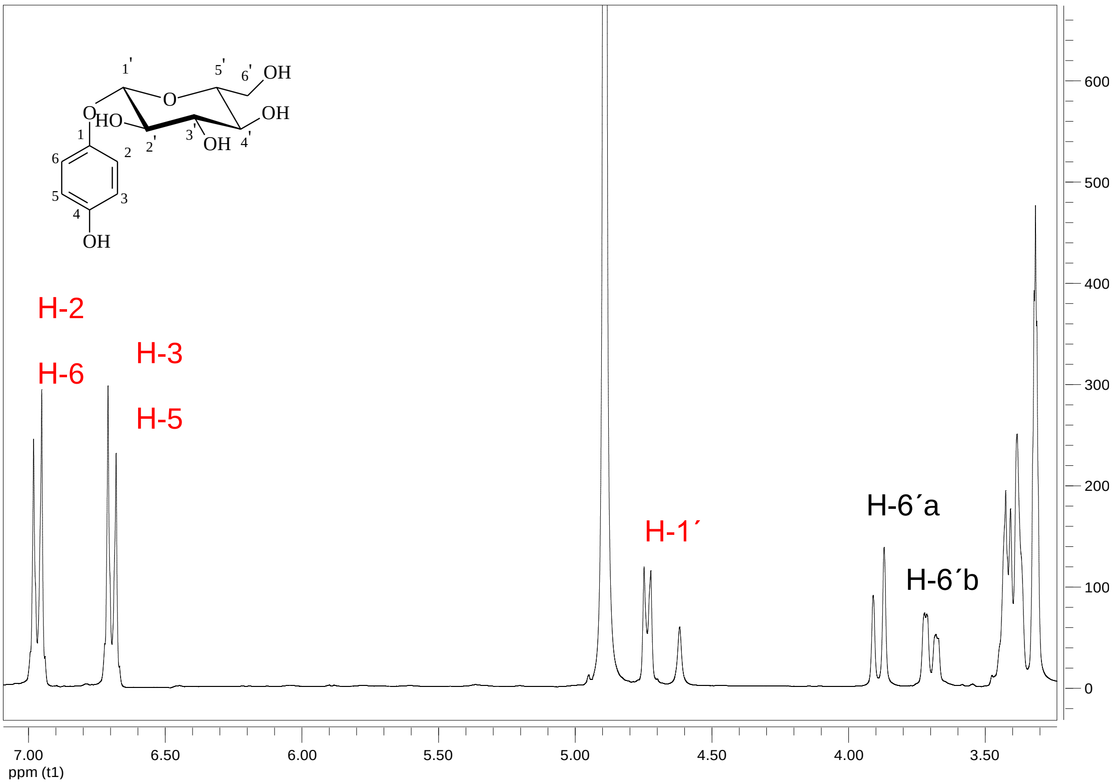

Figure S 83. ^{1}H-NMR spectrum of compound **21** (CD_3OD, 600MHz).

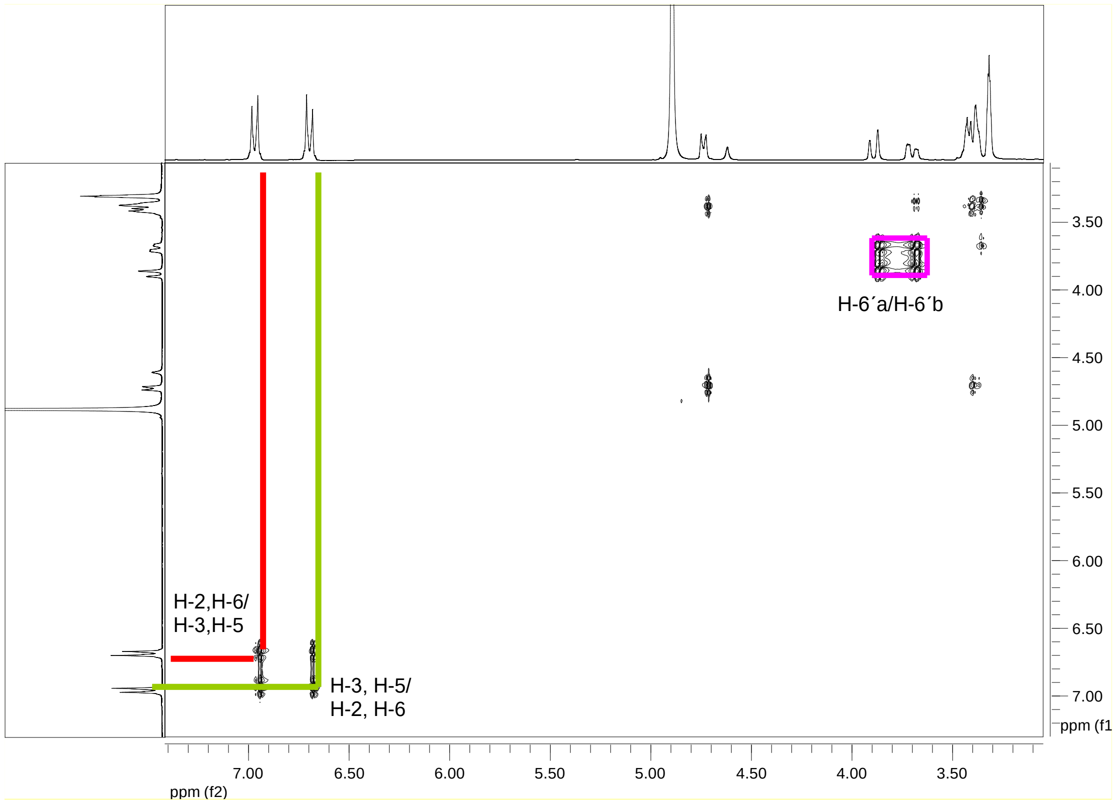

Figure S 84. COSY-NMR spectrum of compound **21** (CD_3OD, 600MHz).

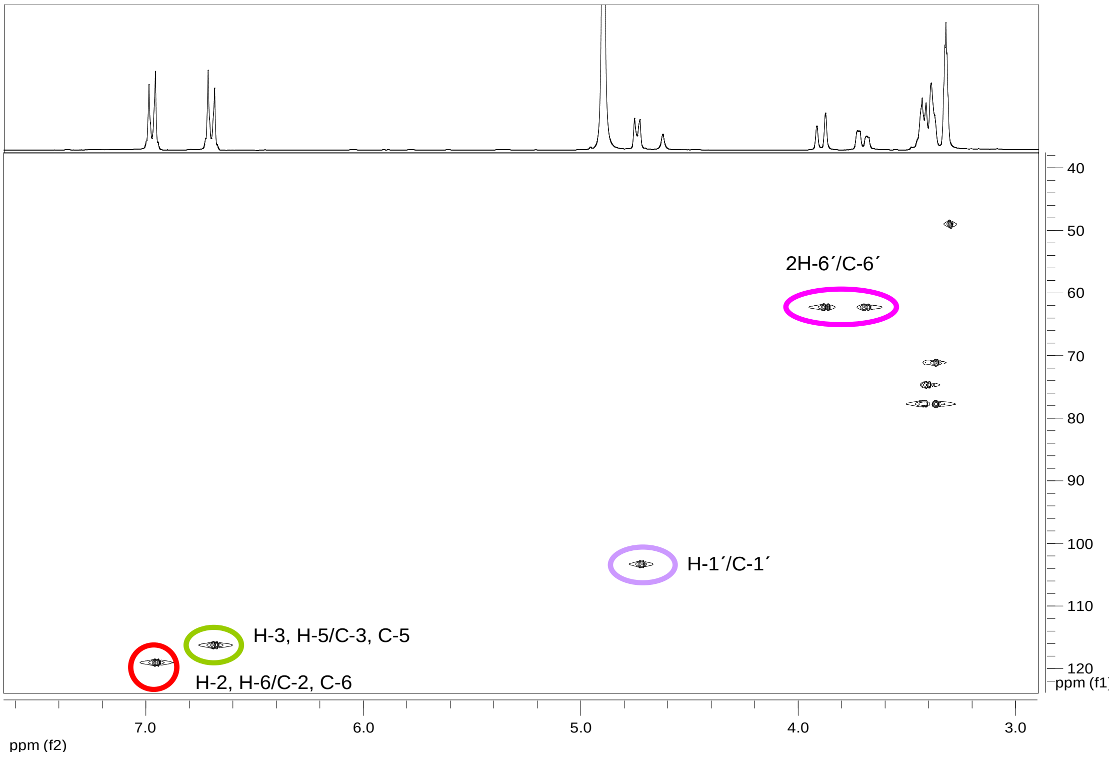

Figure S 85. HMQC-NMR spectrum of compound **21** (CD_3OD, 600MHz).

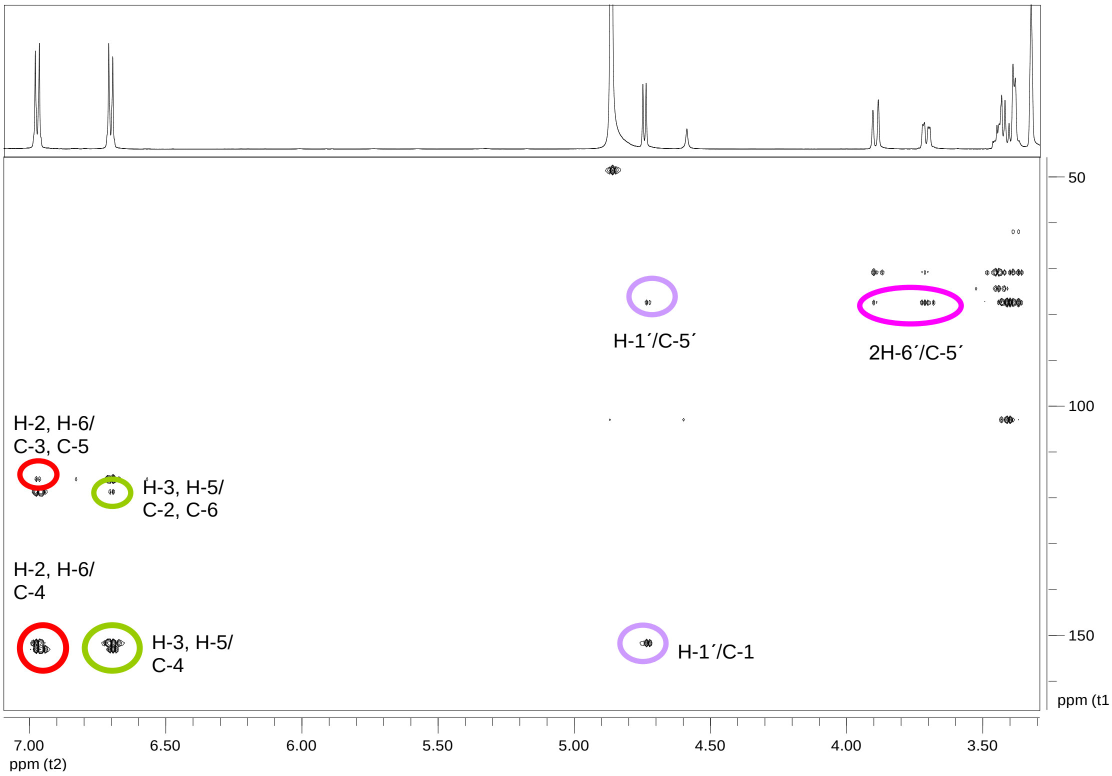

Figure S 86. HMBC-NMR spectrum of compound **21** (CD_3OD, 600MHz).

Table S26: ^{1}H NMR of compound **22** (CD_3OD, 500MHz).

δ (ppm)	nºH	Multiplicity, J (Hz)	Position
6.84	1	*d*(J=3.0)	H-2
6.78	1	*dd* (J=3.0, 9.0)	H-6
6.65	1	*d* (J=9.0)	H-5
5.30	1	*tt* (J=1.5, 7.5)	H-2´
4.71	1	*d* (J=7.5)	H-1´´
3.87	1	*dd* (J=2.0, 12.0)	H-6´´a
3.70	1	*dd* (J=5.0, 12.0)	H-6´´b
3.38-3.42	4	*m*	H-2´´, 3´´, 4´´, 5´´
3.24	2	*d* (J=7.0)	H-1´
1.73	3	*s*	H-5´
1.70	3	*s*	H-4´

Table S27: ^{13}C NMR of compound **22** (CD_3OD,75MHz).

δ (ppm)	Position
151.3	C-1
150.4	C-4
132.2	C-3´
129.1	C-3
122.9	C-2´
118.9	C-2
115.2	C-5
115.0	C-6
102.4	C-1´´
76.9	C-3´´
74.1	C-5´´
73.9	C-2´´
70.4	C-4´´
61.5	C-6´´
28.5	C-1´
24.9	C-5´
16.8	C-4´

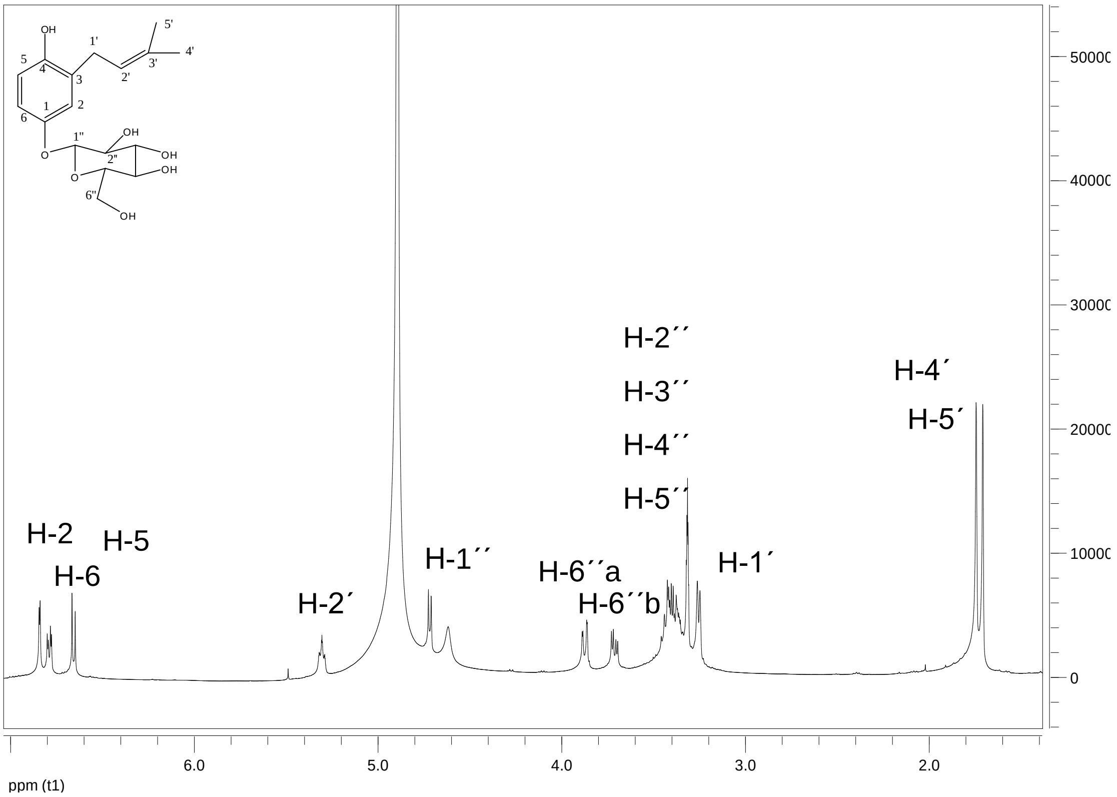

Figure S 87. ^{1}H-NMR spectrum of compound **22** (CD_3OD, 500MHz).

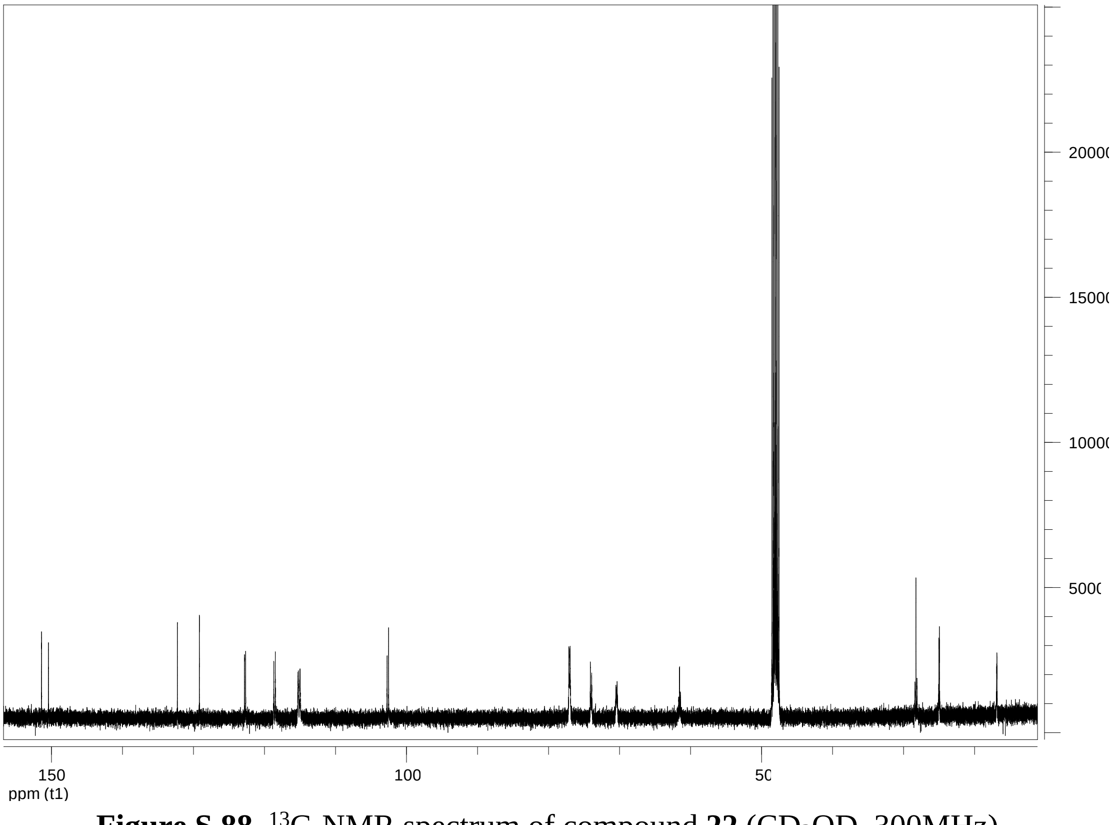

Figure S 88. ^{13}C-NMR spectrum of compound **22** (CD_3OD, 300MHz).

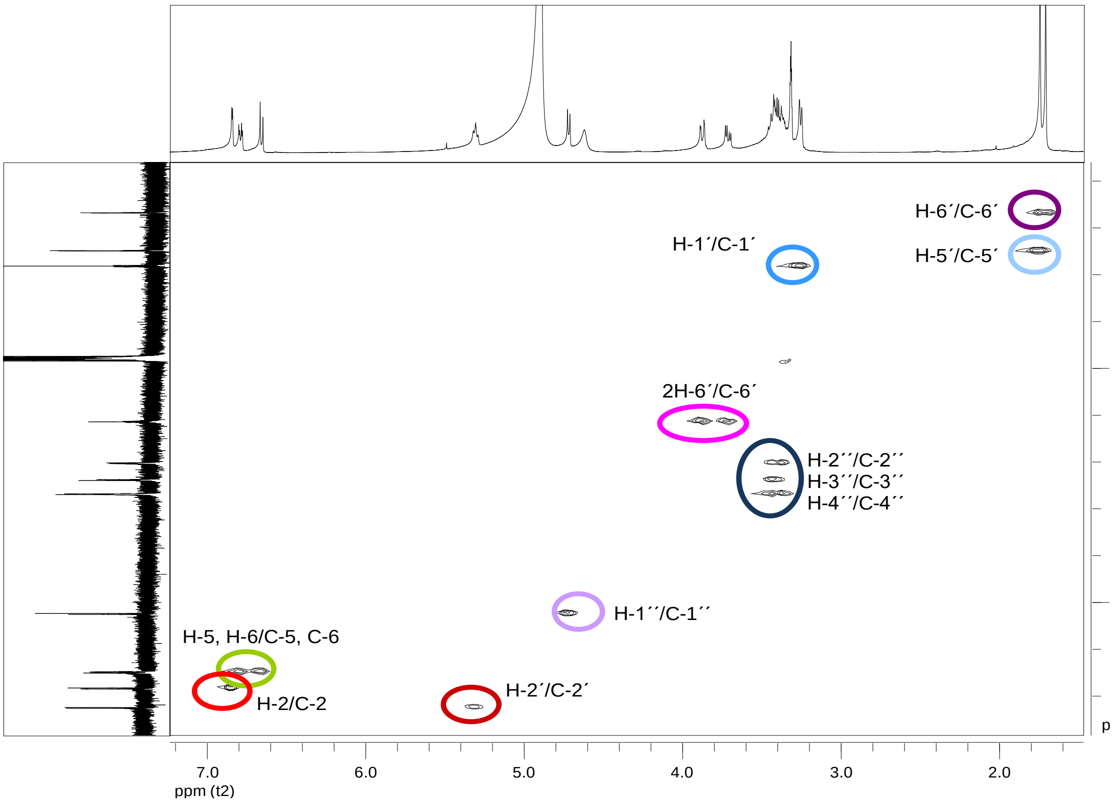

Figure S 89. HMQC-NMR spectrum of compound **22** (CD_3OD, 500MHz).

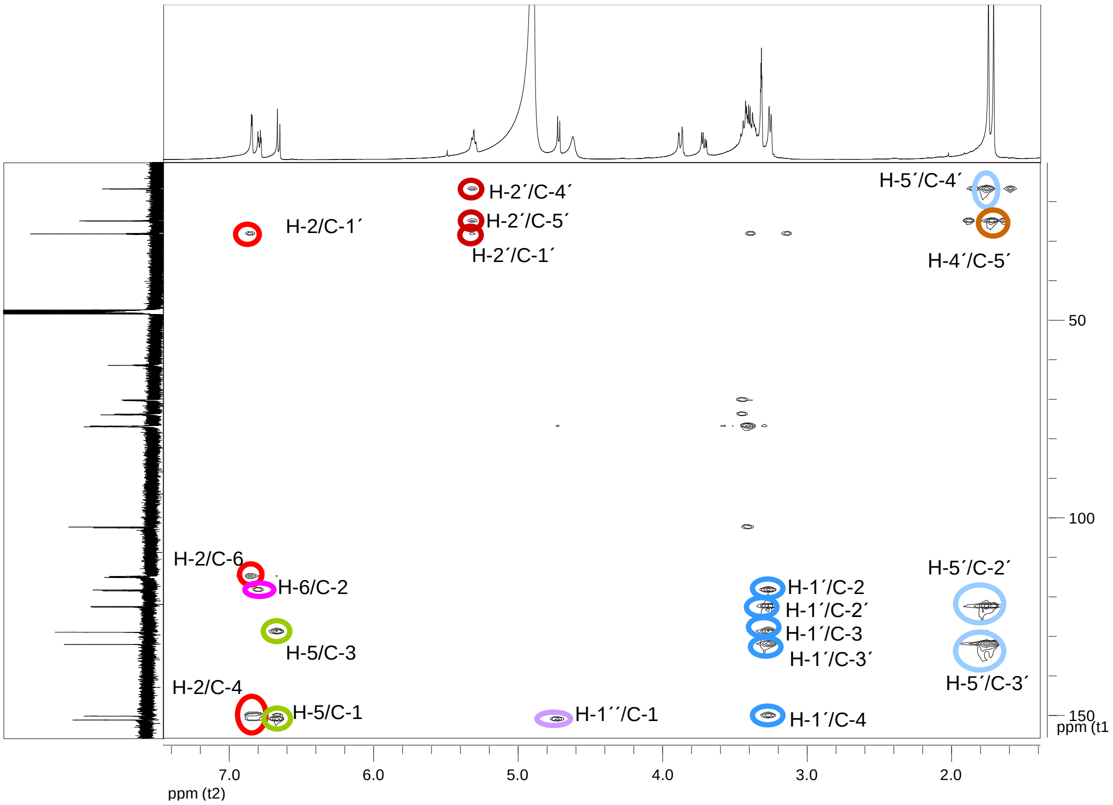

Figure S 90. HMBC-NMR spectrum of compound **22** (CD_3OD, 500MHz).

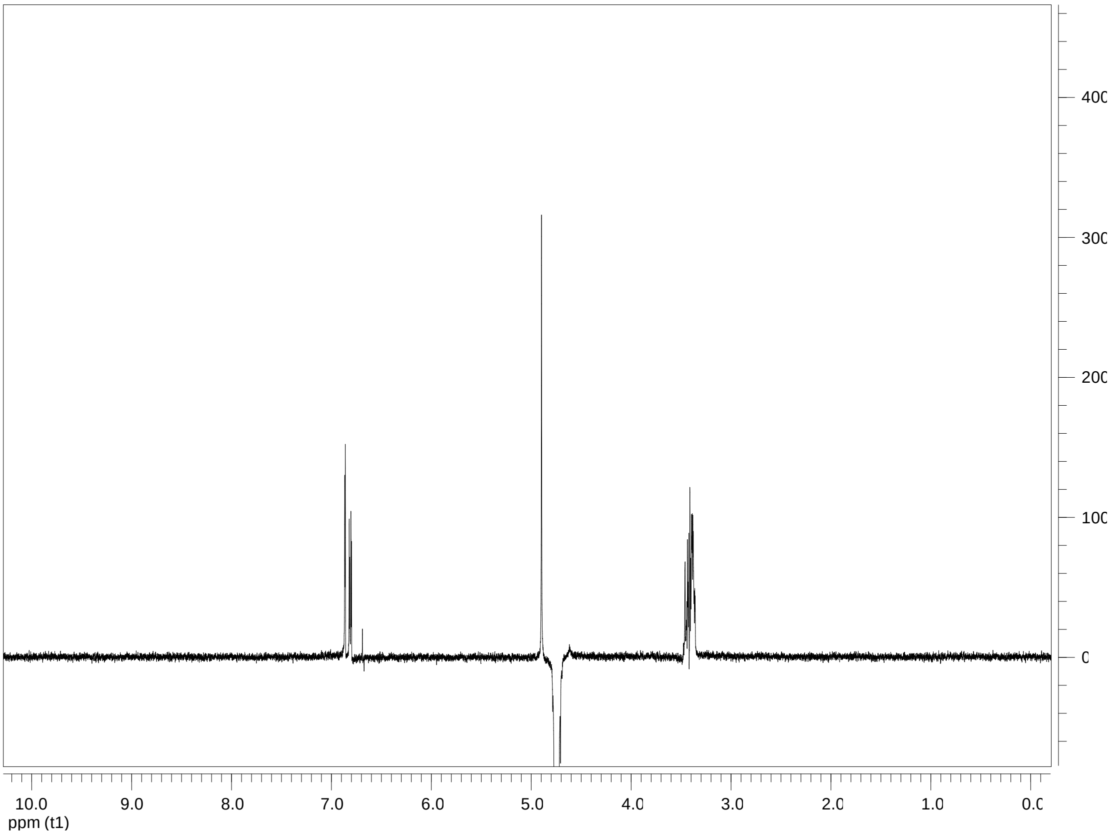

Figure S 91. NOE-NMR spectrum of compound **22** (CD_3OD, 500MHz).

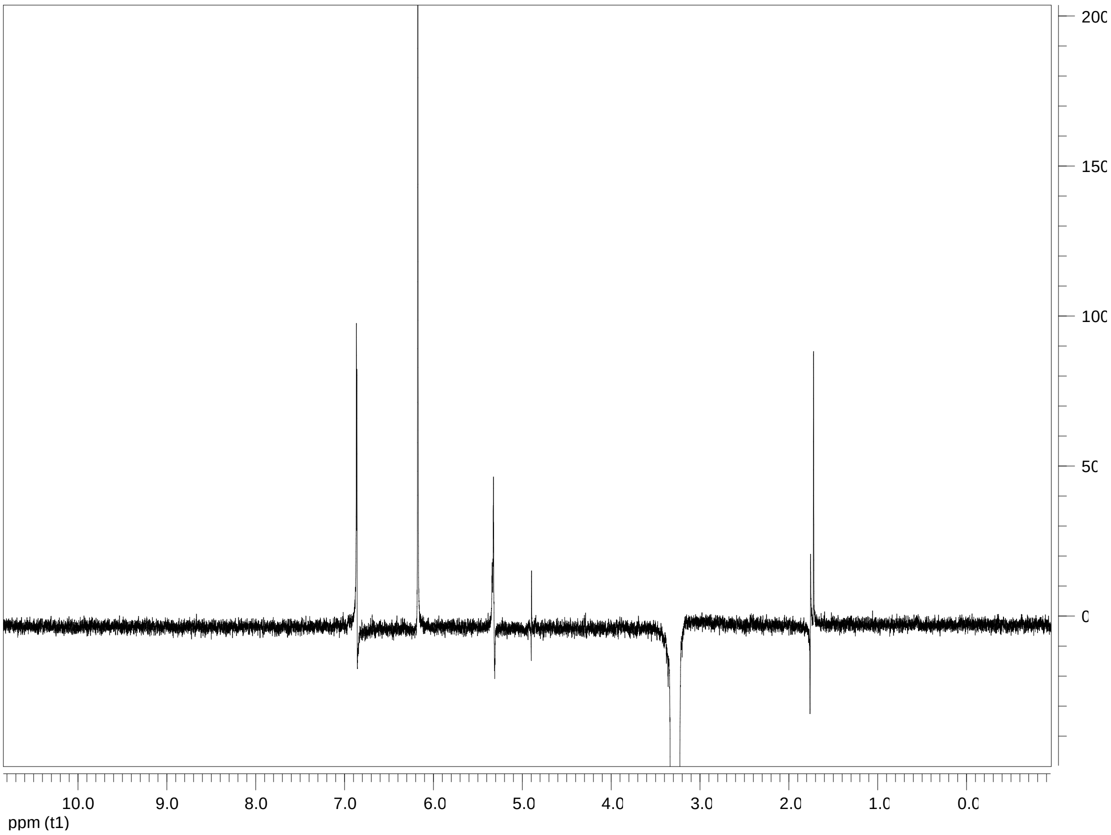

Figure S 92. NOE-NMR spectrum of compound **22** (CD_3OD, 500MHz).
